# Highly selective synthesis and near-infrared photothermal conversion of metalla-Borromean ring and [2]catenane assemblies†

**DOI:** 10.1039/d2sc00437b

**Published:** 2022-04-05

**Authors:** Li-Long Dang, Ting-Ting Li, Ting-Ting Zhang, Ying Zhao, Tian Chen, Xiang Gao, Lu-Fang Ma, Guo-Xin Jin

**Affiliations:** College of Chemistry and Chemical Engineering, Luoyang Normal University, Henan Province Function-Oriented Porous Materials Key Laboratory Luoyang 471934 P. R. China; Shanghai Key Laboratory of Molecular Catalysis and Innovative Materials; State Key Laboratory of Molecular Engineering of Polymers, Department of Chemistry, Fudan University Shanghai 200438 P. R. China gxjin@fudan.edu.cn; College of Chemistry and Bioengineering (Guangxi Key Laboratory of Electrochemical and Magnetochemical Functional Materials), Guilin University of Technology Guilin 541004 P. R. China

## Abstract

Although the selective synthesis of complicated supramolecular architectures has seen significant progress in recent years, the exploration of the properties of these complexes remains a fascinating challenge. Herein, a series of new supramolecular topologies, metalla[2]catenanes and Borromean ring assemblies, were constructed based on appropriate Cp*Rh building blocks and two rigid alkynyl pyridine ligands (L1, L2) *via* coordination-driven self-assembly. Interestingly, minor differences between the two rigid alkynyl pyridine ligands with/without organic substituents led to products with dramatically different topologies. Careful structural analysis showed that π–π stacking interactions play a crucial role in stabilizing these [2]catenanes and Borromean ring assemblies, while also promoting nonradiative transitions and triggering photothermal conversion in both the solution and the solid states. These results were showcased through comparative studies of the NIR photothermal conversion efficiencies of the Borromean ring assemblies, [2]catenanes and metallarectangles, which exhibited a wide range of photothermal conversion efficiencies (12.64–72.21%). The influence of the different Cp*Rh building blocks on the NIR photothermal conversion efficiencies of their assemblies was investigated. Good photothermal conversion properties of the assemblies were also found in the solid state. This study provides a new strategy to construct valuable half-sandwich-based NIR photothermal conversion materials while also providing promising candidates for the further development of materials science.

## Abstract 2

The selective synthesis of three kinds of supermolecular topologies, molecular Borromean ring, [2]catenane and metallarectangle based on two alkynyl ligands is presented. Remarkably, the NIR photothermal conversion efficiency was found to improve as the π–π stacking increases.

## Graphical abstract

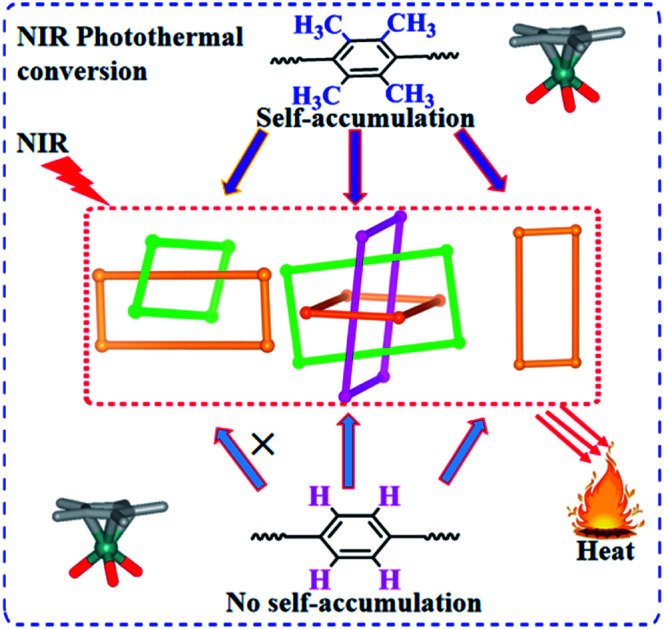

## Introduction

In recent decades, supramolecular coordination chemistry has made immense progress in the synthesis of highly complex structures and their potential prospects in applications, such as molecular machines, biomimetic materials, photothermal effects, among others.^1–5^ Thereby, a series of complicated coordination supramolecular topologies, such as metalla[2–4]catenanes,^6–12^ rotaxanes,^13–17^ molecular cages,^18–23^ and various knots,^24–31^ have been successfully prepared according to a range of construction strategies by an ever-growing group of scientists. These synthetic strategies include hydrogen bonding induction,^32–35^ metal-ion-templated self-assembly and template-free self-assembly methods, with the latter two representing the most commonly used synthetic methods.^24^ The impressive syntheses of a number of intricate topologies, such as [2, 3, 4]catenanes,^36,37^ 8_19_,^38^ +3_1_#+3_1_#+3_1_,^39^ granny,^40^ 5_1_,^41^ 5_2_,^42^ and 7_4_ knots,^43^ underlines the importance of metal-ion-directed self-assembly, and has led to a boom in the development of supramolecular chemistry.

Alternatively, template-free self-assembly strategies have shown significant advantages in the synthesis and structural transformation of aesthetically pleasing supramolecular structures, thanks mainly to the dynamic and reversible nature of metal–ligand coordination. Using this strategy, a series of higher-order topologies and their structural responses have now been explored. Fujita and coworkers reported a number of catenanes and a 7_1_ knot through the coordination of Ag^+^ ions and pyridine peptides, but their structural transformations were not studied.^34,35^ The group of Jin has reported the selective construction of a series of intricate structures, such as molecular Borromean ring assemblies,^44–51^ Solomon links,^52–54^ 3_1_,^55^ double trefoil,^56^ and 4_1_ knots,^57^ and [2, 3, 4]catenanes.^24,58^ The topological transformations between these structures were explored in detail through concentration-, solvent- and host–guest chemistry effects or *via* the chemical reactivity of the metal ions and organic ligands.^47,59^ After careful analysis of the structural characteristics of these supramolecular topologies, we have found that although a number of different forces, such as hydrogen bonding interactions,^57^ hydrophobic effects, and others,^49^ play diverse roles in the formation of these complexes, the decisive factor in the generation of these structures is generally π–π stacking interactions. These topological transformations are mainly induced through the reduction or enhancement of these accumulated interactions. Therefore, detailed studies of the π–π stacking interactions in these assemblies are critical to the advancement of their supramolecular chemistry.

It has been established that three conditions need to be satisfied for the formation of π–π stacking interactions: (i) two strong electron-rich conjugated planes or alternating electron-donor and electron-acceptor conjugated planes; (ii) a suitable parallel distance between the planes (3.4–4.2 Å); and (iii) external forces are best removed to avoid the interference of steric hindrance. If these three conditions are met simultaneously, π–π stacking interactions can be realized and complicated topologies might be formed. Previous reports of supramolecular topologies have also revealed that π–π stacking can promote nonradiative transitions and trigger photothermal conversion in both the solution and the solid state,^60–62^ which inspired us to explore the photothermal conversion abilities of the complicated topologies prepared in our laboratories.

**Fig. 1:**
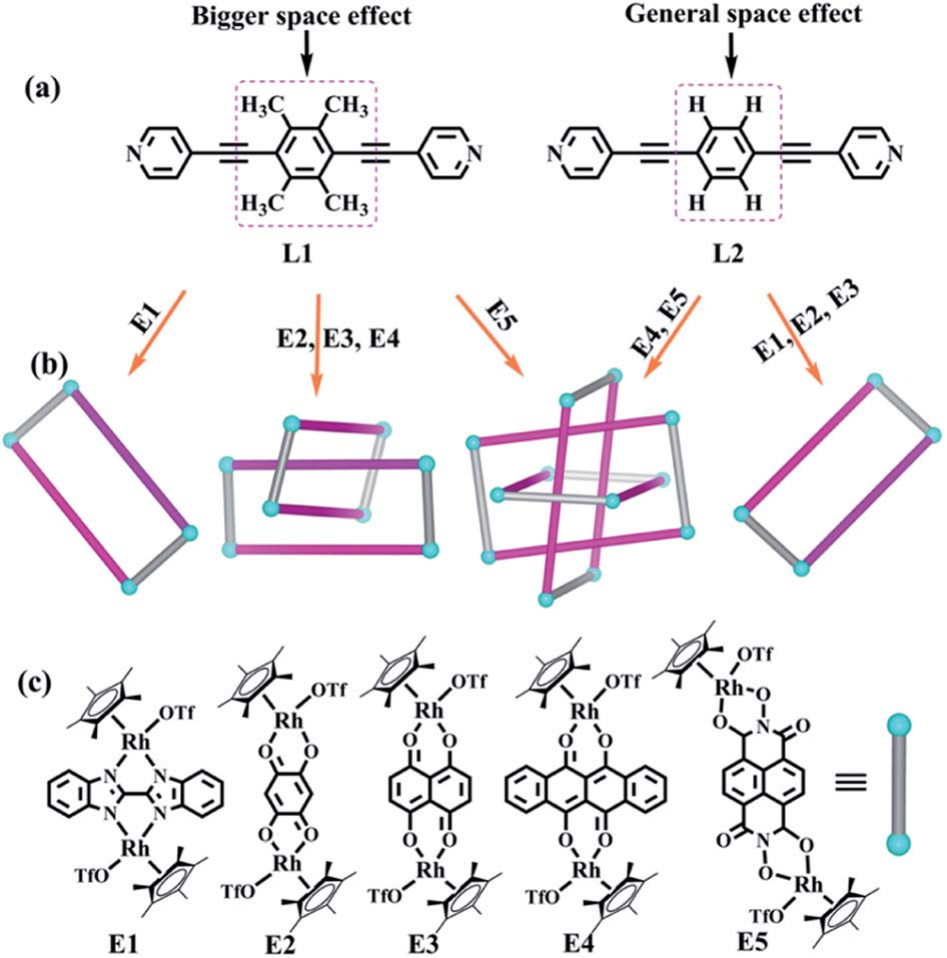
Features of ligands used in this work for synthesizing interlocked topologies; (b) topological representation of interlocked complexes prepared in this work; (c) dinuclear building blocks applied frequently by our research group.

In view of the requirements for the formation of π–π stacking interactions, two new rigid ligands were selected: 4,4′-((2,3,5,6-tetramethyl-1,4-phenylene)bis(ethyne-2,1-diyl))dipyridine (L1, strong electron-rich center and larger steric hindrance) and 1,4-bis(pyridin-4-ylethynyl)benzene (L2, weak electron-rich center and little steric hindrance) (Fig. 1). By comparing the complexes built from ligands L1 and L2, we found that ligand L1 could result in strong accumulation effects and generate more intricate structures, promoting nonradiative transitions and triggering efficient photothermal conversion.

The two similar, rigid alkynyl pyridine ligands (L1, L2) were subsequently utilized to prepare metal-directed assemblies. The ligands differ only in their central phenylene group: L2 has a simple C_6_H_4_ group at its centre, while L1 contains a tetramethylphenylene group (C_6_Me_4_). By combining these two ligands with the appropriate Cp*Rh building blocks (E1–E5), three kinds of novel supramolecular topologies were constructed: metallarectangles, [2]catenanes, and Borromean ring assemblies. Single-crystal X-ray diffraction analysis showed that the tetramethylphenylene group of L1 allows self-accumulation, resulting in the formation of [2]catenanes by combination with E2 or E3. Meanwhile, the assembly of L2 with E2 or E3 under the same conditions resulted in simple metallarectangles in both cases. The building block E4, which has a larger conjugated plane, leads to a Borromean ring assembly with L2, while with the larger L1 a [2]catenane was obtained. When the larger building block E5 was used, two new Borromean rings were obtained based on ligands L1 and L2, which might be due to the Rh–Rh nonbonding distance (11.8 Å) being large enough to avoid the steric hindrance from the methyl groups of L1, thereby allowing the formation of π–π stacking between central phenyl unit of L1 and E5. In addition, good photothermal conversion properties of the assemblies were found in both the solution and the solid state. Remarkably, the NIR photothermal conversion efficiency increased as the π–π stacking increased in the solution state, and a wide range of photothermal conversion efficiencies (12.64–72.21%) were observed. This work demonstrates that by altering the substituents at the backbone of the bridge ligands, the electron density and the steric hindrance of the central arene ring unit is varied, allowing a number of distinct assembly processes.

## Results and discussion

### Self-assembly of tetranuclear macrocycles 1 and 2 based on E1

**Fig. 2:**
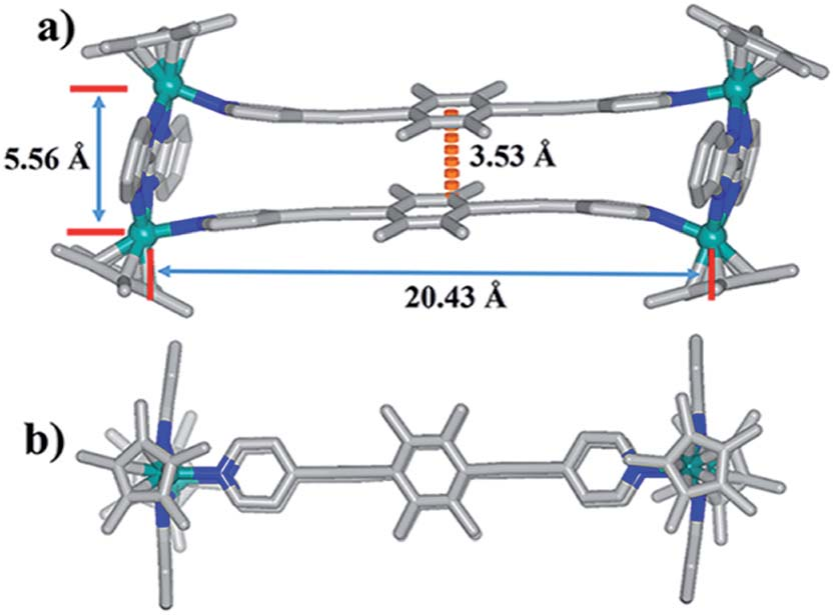
Molecular structure of complex 1: (a) top view, (b) side view. Most hydrogen atoms, anions, solvent molecules and disordered elements are omitted for clarity (N, blue; O, red; C, gray; Rh, aqua).

Under dark conditions, two experiments were carried out whereby [Cp*_2_Rh_2_(BiBzlm)](Cl)_2_ (E1) with AgOTf (2.0 equiv.) were in a mixed CH_3_OH/DMF solution (5 : 1 v/v), followed by the addition of ligand L1 or L2, resulting in the tetranuclear metallarectangles 1 and 2 (yield: 88.2% for 1 and 83.4% for 2; Scheme S1†), respectively. The structures of 1 and 2 were demonstrated clearly by NMR spectroscopy and/or X-ray crystallographic analysis (Fig. 2).

The ^1^H NMR spectrum of 1 in CD_3_OD showed doublets at δ 7.76 and 6.99 ppm and a singlet at *δ* 2.14 ppm corresponding to the protons of L1, while the protons of BiBzlm appeared as two multiplet peaks at *δ* 8.10–8.08 and 7.56–7.55 ppm (Fig. S3, ESI†). The ^1^H DOSY NMR spectrum of 1 showed a single diffusion coefficient (*D* = 2.81 × 10^−10^ m^2^ s^−1^), suggesting that only one stoichiometry of assembly was generated (Fig. S5, ESI†).

Single crystals of complex 1 were acquired by slow diffusion of diethyl ether into the methanol mother liquor. 1 was determined to be a tetranuclear metallarectangle by single-crystal X-ray diffraction analysis (Fig. 2). The two binuclear edge units (E1) are linked by two dipyridyl ligands L1, accompanied by short Rh–Rh nonbonding distances (5.56 Å) and long Rh–Rh nonbonding distances (20.43 Å). Moreover, the shortest distance between two phenyl units of ligands L1 is *ca.* 3.53 Å. This small internal space in 1 likely hinders the generation of interlocked metallarectangles or other more complicated species. The same principle could also be applied to complex 2, the NMR spectrum of which reflected the formation of a tetranuclear metallarectangle (Fig. S6–S8†).

### The selective construction of [2]catenanes based on E2, E3

**Scheme 1:**
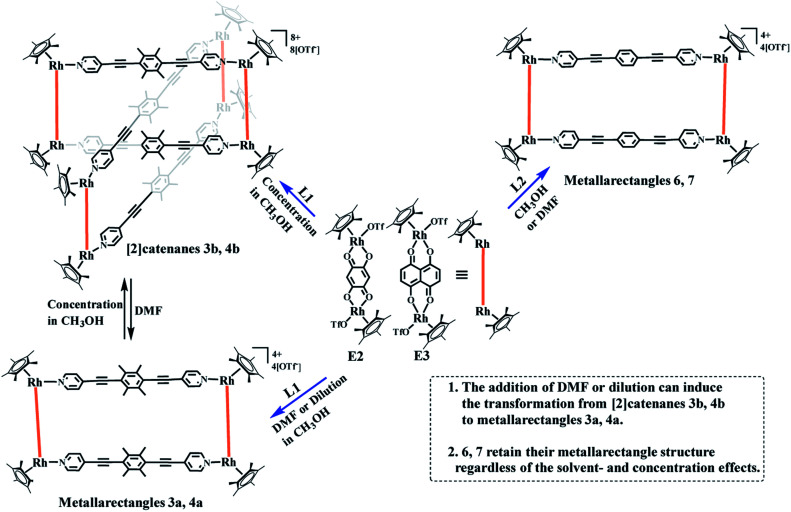
Synthesis of metallarectangles 3a, 4a, 6, 7, [2]catenanes 3b, 4b and the transformations between 3a and 3b, 4a and 4b.

The presence of four electron-donating methyl units in L1 was predicted to enhance the electron density of benzene unit, thus promoting the formation of π–π stacking interactions more than in the phenylene-containing ligand L2 and resulting in the generation of a more complicated topology. Two longer building blocks, E2, E3 (Rh–Rh nonbonding distances: 7.93 Å, 8.36 Å, respectively), were chosen to react with ligand L1 in methanol. Each mixture was stirred for 8 h, resulting in brown and dark brown solutions, respectively. A 1 : 1 mixture of methanol and isopropyl ether as transition layer solution was gradually added to the above solutions until the transition layer became colorless (Scheme 1), at which point an equivalent volume of isopropyl ether was added. Fortuitously, a series of crystalline complexes based on E2 were acquired in high yields (88.5%).

The crystals were initially added to CD_3_OD to observe their solution behavior. At low concentrations (*ca.* 0.5 mM), the ^1^H NMR spectrum indicated a simple set of signals. Similar to compound 1, there are two doublet signals at 8.29, 7.59 ppm and a singlet at 5.67 pm in the aromatic region, corresponding to the signals of the L1 pyridine and E2 phenylene ring protons. The signals for the central –CH_3_ groups of L1 and the Cp* protons were found at 2.34 and 1.70 ppm, reflecting the formation of the monorectangle complex 3a (Fig. S9–S11†). The concentration was then gradually increased and changes in the NMR signals were observed. The initial signals in the ^1^H NMR spectrum slowly decreased in intensity, while a set of new signals appeared and rose with increasing concentration, suggesting the transformation from a simple monorectangle to an intricate complex 3b (Fig. 4). Finally, the initial signals almost disappeared completely, suggesting transformation of the monorectangle complex into the intricate structure 3b, which was analysized by the ^1^H–^1^H COSY and ^1^H–^1^H DOSY NMR spectroscopy (Fig. S18†). All proton resonances were found to be split into two signals rather than the simple resonances previously observed for 3a. The resonances belonging to the pyridinyl protons were recorded as four doublets at 8.42, 8.35, 7.63, and 7.59 ppm with identical intensity. Besides, two sets of signals were observed for the Cp* protons at 1.74 and 1.69 ppm in an intensity ratio of 1 : 1. All ^1^H NMR signal assignments were supported by ^1^H–^1^H COSY NMR data (Fig. S15, S16†). Furthermore, the ^1^H–^1^H DOSY NMR spectrum of 3b (Fig. S17†) confirmed that all aromatic and Cp* proton signals featured a single diffusion constant (*D* = 2.94 × 10^−10^ m^2^ s^−1^), suggesting that all resonances belong to just one assembly. The results are reasonable according to Le Chatelier's principle, *i.e.* changes in concentration should promote a reversible conversion between the catenane structure and the corresponding metallarectangle due to the dynamic metal–ligand bonds and weak interactions between the subunits. In general, coordination-driven self-assembly favors the formation of interlocked metallasupramolecules at high concentrations, while disfavoring interlocked structures at lower concentrations. This assumption was confirmed by an X-ray diffraction study with crystals of 3b, confirming the formation of a metalla[2]catenane (Fig. 5).

**Fig. 3:**
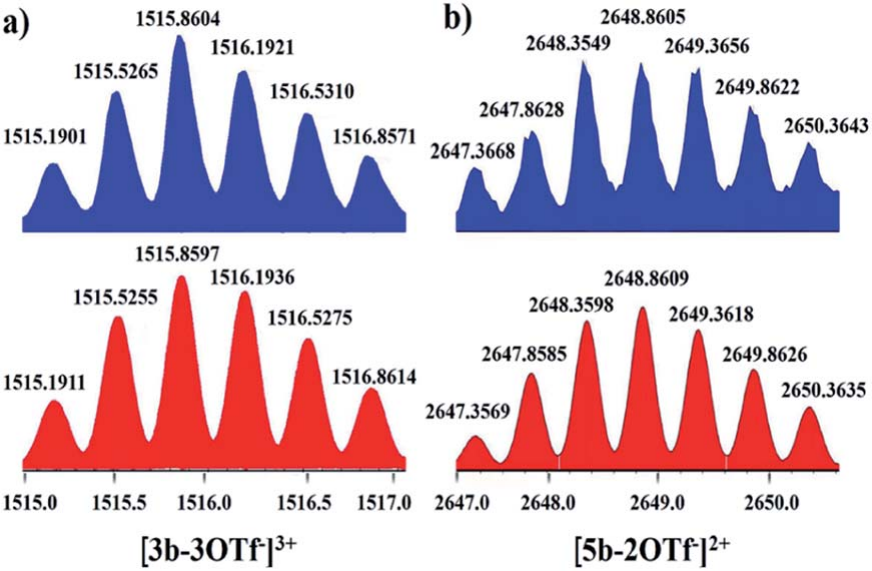
Calculated (bottom, red) and experimental (top, blue) ESI-MS spectra of the 3+ ion of [2]catenane 3b (a) and the 2+ ion of [2]catenane 5b (b).

Nevertheless, single crystals of 3b suitable for X-ray diffraction analysis were obtained at high concentration (20.0 mM in methanol) by slow vapor diffusion of diethyl ether into a saturated methanolic solution of 3b. The molecular structure determination confirmed the formation of the interlocked metalla[2]catenane. Complex 3b is stabilized by strong sandwich-type π–π stacking interactions between the four phenyl moieties of L1 from two metallarectangles, with distances between the benzene ring planes of 3.72, 3.59, and 3.63 Å, respectively (Fig. 5c). Along with the NMR spectroscopic data, ESI-MS also indicated the presence of complex 3b in solution: [3b-3OTf^−^]^3+^ (*m*/*z* = 1915.19) (Fig. 3a, S61†).

**Fig. 4:**
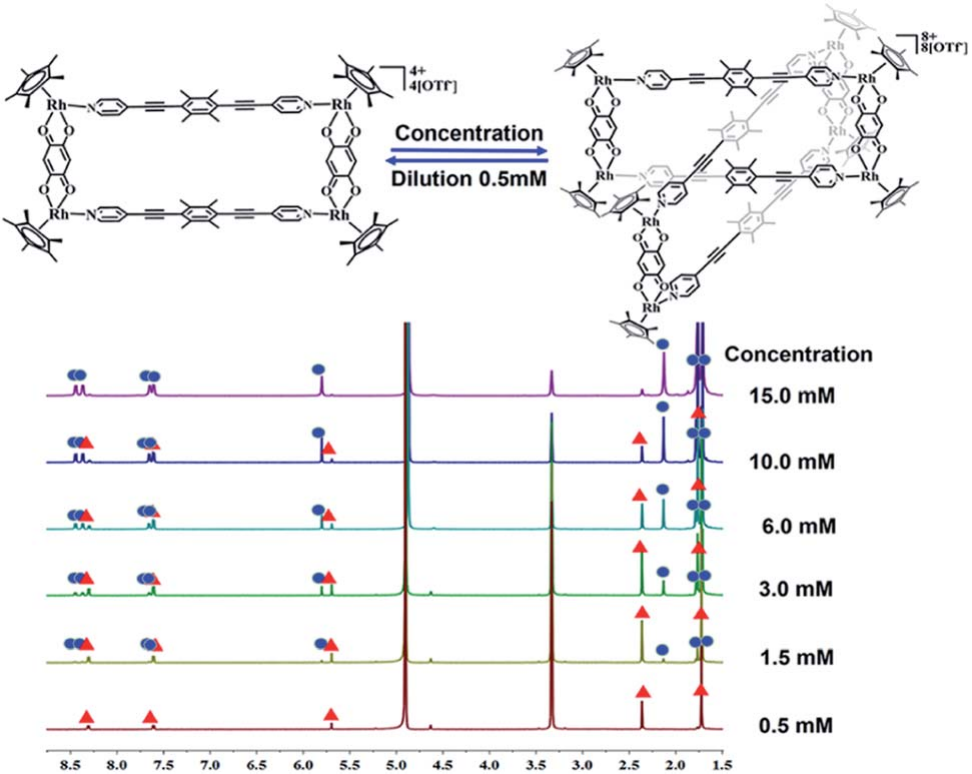
The ^1^H NMR spectra of metallamacrocycle 3a (500 MHz, ppm) in CD_3_OD (0.5 mmol), showing the transformation of metallamacrocycle 3a into metalla[2]catenane 3b with increasing concentration (0.5–15.0 mM).

**Fig. 5:**
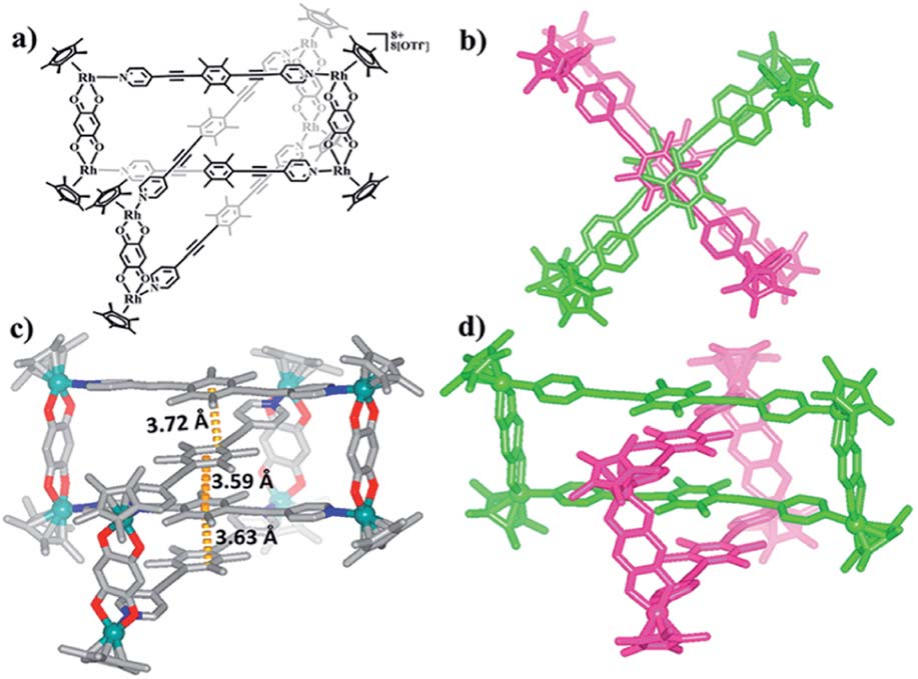
(a) Chemical structure of [2]catenane 3b; (b) top view of the structure of 3b; (c) solid-state structure of 3b, showing the π–π stacking interactions. (d) Side view of the structure of 3b.

**Fig. 6:**
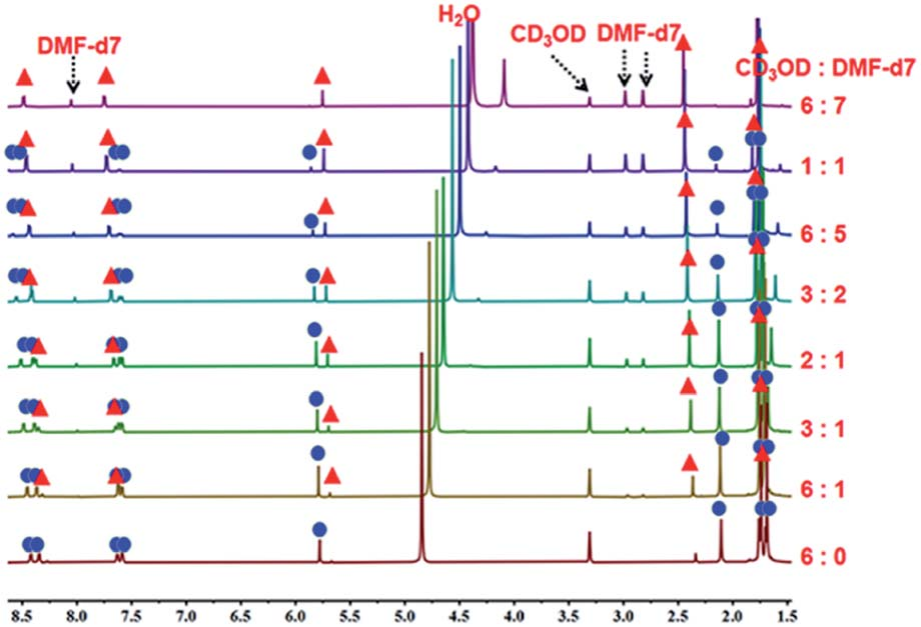
The ^1^H NMR spectra of metalla[2]catenane 3b (500 MHz, ppm) in CD_3_OD (20.0 mmol), showing the transformation of 3b into the metallamacrocycle 3a upon addition of DMF-d_7_.

Recently, a number of experimental reports on self-assembled architectures have indicated that the relatively rigid yet dynamic nature of coordination bonds in supermolecular structures provide distinct advantages for the study of supramolecular transformations. A wide range of external stimuli, such as solvent, concentration, and guest templates, have been shown to trigger transformations within metallosupramolecular architectures. We have also reported solvent-induced supramolecular transformations between metalla[2]catenanes and the corresponding simple complexes (Fig. 6).^8,24^

A ^1^H NMR titration experiment was carried out to further characterize the transformation process from 3b to 3a by adjusting the proportion of DMF-d_7_ and CD_3_OD in the solution. Firstly, a 20.00 mM solution of 3b in pure CD_3_OD was prepared, and then DMF-d_7_ solution was added gradually (CD_3_OD/DMF-d_7_ ratio from 6 : 0 to 6 : 7, v/v). The ^1^H NMR spectra (Fig. 6, S19, S20†) clearly showed the replacement of the proton resonances of 3b by a set of new, simple signals for 3a upon addition of DMF-d_7_, ultimately indicating that 3b had almost completely disappeared and only monorectangle 3a was present in the solution. This transformation was ascribed to a weakening of the π–π stacking interactions by the DMF molecules.

NMR spectroscopic studies of the concentration effects with the complex based on E3 were also carried out. At low concentration, the ^1^H NMR spectra show a set of simple signals. The two doublets at 8.40, 7.52 ppm and a singlet at 2.32 ppm derive from the pyridyl and methyl groups of ligand L1, and the two singlets at 7.26, 1.62 ppm are attributed to the phenylene ring and Cp* protons of E3 (Fig. S21–S23†). Moreover, the NMR spectra also clearly displayed the disappearance of the simple signals and the appearance of another set of signals, suggesting the structural transformation from simple metallarectangle 4a to [2]catenane 4b based on concentration effects (Fig. S24, S25†).

Meanwhile, the respective reactions of E2 and E3 with L2 were performed in methanol solution, and after stiring for 8 h, two complexes (6, yield: 85.3%; 7, yield: 82.4%) were obtained. Single-crystal X-ray diffraction and NMR spectroscopic studies were carried out to determine both structures.

**Fig. 7:**
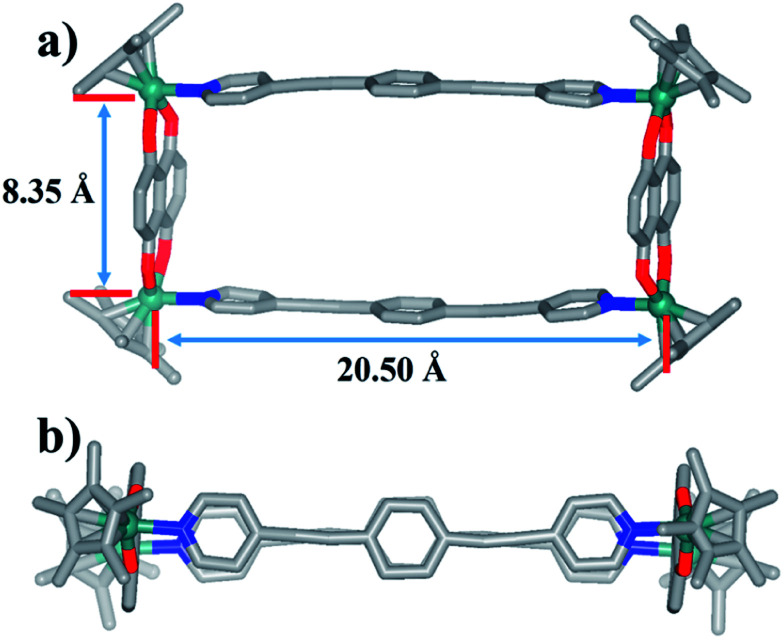
Molecular structure of complex 7. (a) Side view, (b) top view. Most hydrogen atoms, anions, solvent molecules and disordered elements are omitted for clarity (N, blue; O, red; C, gray; Rh, aqua).

The ^1^H NMR spectra of 6 and 7 show simple signals regardless of concentration and solvent changes, suggesting the formation of two simple metallarectangles (Fig. S36, S39, S40, S41†), in contrast to compounds 3b (Fig. S15†) and 4b (Fig. S25†). Single crystals of 7 suitable for X-ray diffraction analysis were acquired by slow diffusion of isopropyl ether into its methanol mother liquor. Unlike complexes 3b and 4b, 7 was determined to be a stable tetranuclear metallarectangle (Fig. 7) comprising two dinuclear edge units (E3) linked by two dipyridyl ligands L2. The structural analysis showed that the Rh–Rh nonbonding distances were 8.35 Å (short) and 20.50 Å (long). From the perspective of size, the cavity is large enough to allow the formation of π–π interactions between two phenylene groups of L2 or between the phenylene groups of L2 and the edge unit E3, if another ring was to be threaded inside the rectangle. However, the absence of complicated threaded structures might reflect that the weakly-donating phenylene conjugated unit (*i.e.* without methyl groups) is not conducive to the formation of accumulation interactions. The same principle could also be applied to complex 6. These results reflect that the tetramethylphenylene unit in L1 has a stronger donating effect, and is thus more conducive to the formation of π–π stacking interactions, than the simple phenylene group in L2.

### The selective construction of molecular [2]catenane and Borromean ring based on E4

**Scheme 2:**
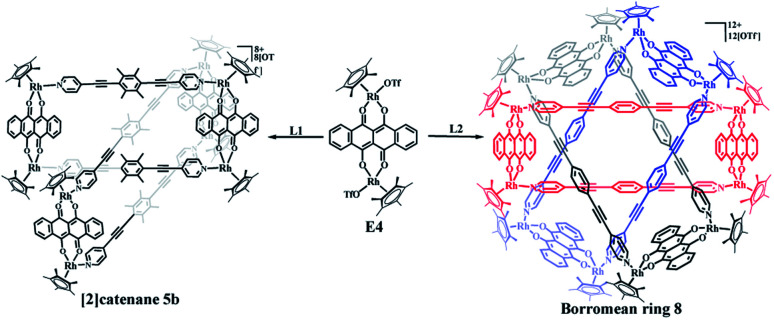
Directed synthesis of [2]catenane 5b and Borromean rings assembly 8.

The building blocks E3 and E4 are significantly wider than E2, but have similar Rh–Rh distances (8.35 Å for E3 and 8.46 Å for E4). The building block E4 thus has enough space to allow one L1 ligand to thread another identical metallarectangle, possibly forming a catenane. Therefore, E4 was chosen to target a [2]catenane by self-assembly with ligand L1 in a 1 : 1 molar ratio in methanol solution, providing a brown complex (yield: 90.0%) (Scheme 2). Fortuitously, single crystals suitable for X-ray diffraction were obtained and the structure of this complex was confirmed by NMR spectroscopy, ESI-MS, and single-crystal X-ray diffraction analysis.

Similar to compound 3a, the initial ^1^H NMR spectroscopic signals of this complex were very simple at low concentration (*ca.* 0.5 mM). The ^1^H NMR spectrum showed doublets at *δ* 8.47 and 7.39 ppm corresponding to the pyridinyl protons of L1, while the two multiplets at 8.75–8.73 ppm and 7.97–7.95 ppm correspond to the arene protons of E4. The protons of the methyl groups of L1 and Cp* were located at 2.19 and 1.75 ppm, respectively, in line with the formation of the simple rectangle 5a (Fig. S28–S30†). Moreover, the ^1^H NMR spectrum also showed the presence and enhancement of new signals and the weakening of the initial signals with increasing concentration (Fig. S31, S32†). However, unlike the transformation from 3a to 3b, in which all proton resonances were observed to split into two sets of signals, most of the resonances were found to transform into a new set of simple signals, similar to the transformation between 4a and 4b. Thus, for a CD_3_OD solution of 5b, the two pyridinyl doublets of L1 were observed at 8.56 and 7.31 ppm, showing a slight upfield shift relative to that of 5a (8.47 and 7.39 ppm). In addition, the two multiplets of E4 were located at 8.82–8.80 ppm and 8.03–8.01 ppm, while the signal for the Cp* protons was located at 1.76 ppm. However, the resonance for the –CH_3_ protons of L1 were found to be split into two signals at 1.96 and 1.31 ppm, which was further demonstrated by ^1^H–^1^H DOSY spectroscopy (Fig. S33, S35†). Thus, overall, it was very difficult to determine the topology of 5b merely by ^1^H NMR spectroscopy.

**Fig. 8:**
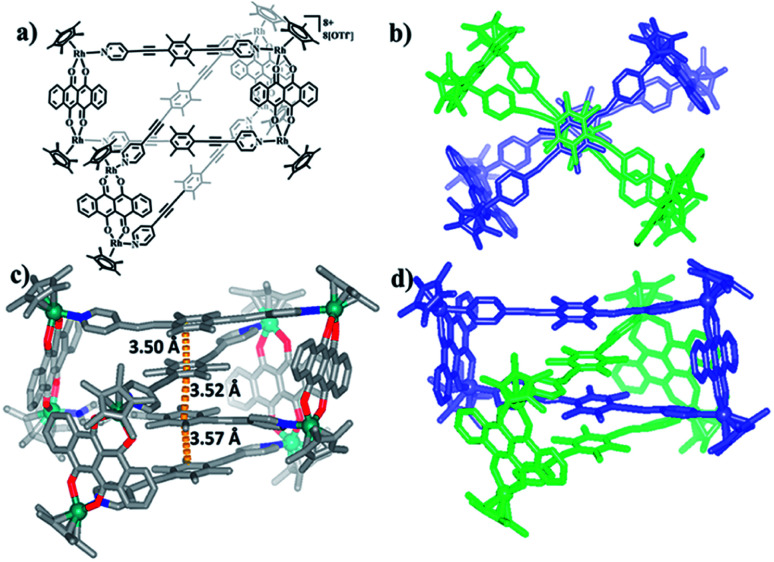
(a) Chemical structure of [2]catenane 5b; (b) top view of the structure of 5b; (c) solid-state structure of 5b showing the π–π stacking interaction; (d) side view of structure of 5b.

Single crystals of 5b were acquired by slow diffusion of diethyl ether vapor into a solution of 5b in methanol and the solid-state structure was demonstrated by X-ray diffraction analysis. The crystal structure of 5b was refined in the *Pna*/21 space group. The crystal structure unequivocally confirmed the topology of a [2]catenane. Obvious triple π–π stacking interactions were observed in the structure of 5b, with separations between central units of 3.50, 3.52 and 3.57 Å, respectively. These are smaller than those of compound 3b (3.72, 3.59 and 3.63 Å), likely reflecting a stronger accumulation effect in the solid state (Fig. 8). ESI-MS data for 5b indicated that the [2]catenane structure of the complex is retained in solution: [5b-2OTf^−^]^2+^ (*m*/*z* = 2647.36) (Fig. 3b, S62†).

**Fig. 9:**
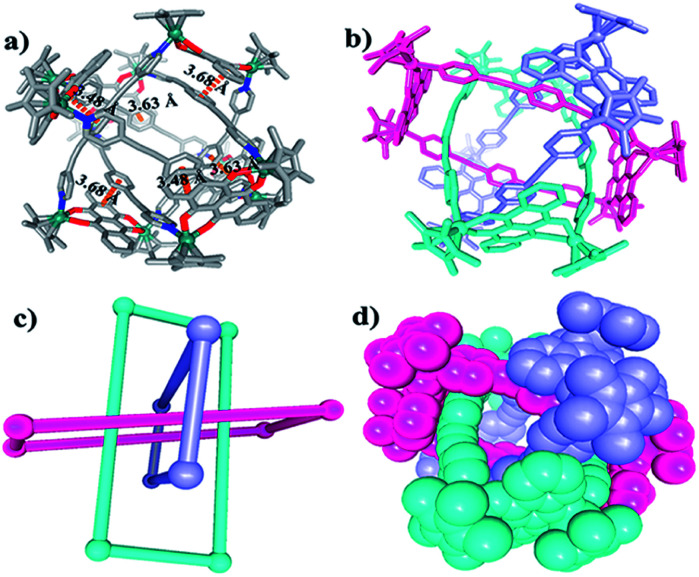
Molecular structure of complex 8. (a) and (b) Representations showing π–π interactions between L1 and the NDI groups of E4; (c) and (d) simplified and space-filling representations; most hydrogen atoms, anions, solvent molecules and disordered elements are omitted for clarity (N, blue; O, red; C, gray; Rh, aqua).

In the ligand L2, the central phenylene unit provides little steric hindrance and only a weakly-donating conjugated plane. The single crystal structures of complexes 6, 7 demonstrate that this ligand is unlikely to allow formation of self-accumulation interactions. However, stacking interactions might be achieved if a strong electron-acceptor unit can pair with the central phenylene group of L2. Therefore, L2 and the dinuclear building block E4, which bears a larger conjugative plane, were combined in a 1 : 1 molar ratio in a methanol solution with the addition of a few drops of DMSO, resulting in a brown complex, 8 (yield: 80.5%) (Scheme 2). The structure of 8 was determined by single-crystal X-ray diffraction and NMR spectroscopy. The ^1^H NMR spectrum showed a lone set of signals, which could be analysed by the combination of ^1^H–^1^H COSY and ^1^H–^1^H DOSY spectroscopy (Fig. S44–S46†). Changing the concentration did not cause the appearance of new signals or the weakening of the original signals, demonstrating the good stability of complex 8 in methanol. Single crystals 8 suitable for single X-ray determination were obtained and the structure of 8 was confirmed to be Borromean ring complex comprising three mechanically interpenetrating but chemically equivalent rings, each of which adopts a rectangle-like conformation with an average length and width of 20.13 and 8.28 Å, respectively. The three distorted rectangles are held together by donor–acceptor stacking interactions (*ca.* 3.5 Å) between the phenyl unit of L2 and the electron-deficient E4 moiety, suggesting that the central phenylene unit of L2 can form π–π stacking interactions, but requires pairing with strong electron-deficient units, which is consistent with our speculation (Fig. 9).

### Self-assembly and response exploration of molecular Borromean rings 9b and 10 based on E5

The structures of complexes 3b, 4b and 5b indicate that π–π stacking interactions play a vital role in the formation of [2]catenanes. Furthermore, stacking interactions might also form between the edge units E1, E2, E3 or E4 and the central phenylene groups of L1 due to their conjugated planes. However, the small sizes of E1, E2, E3 and E4 (with Rh–Rh separations of 5.56, 7.80, 8.35, 8.46 Å, respectively) were insufficient to avoid the steric hindrance of the four –CH_3_ groups in the central phenylene unit of L1. Thus, a larger conjugated unit was chosen, E5, which bears a longer Rh–Rh nonbonding distance (11.8 Å) and potentially provides enough space to avoid the steric hindrance of the –CH_3_ protons, possibly resulting in a more complicated supramolecular structure such as a Borromean ring assembly. In addition, the larger conjugated unit E5 might also form π–π stacking interactions with the ligand L2, thus inducing the formation of a new topologically intricate complex.

**Scheme 3:**
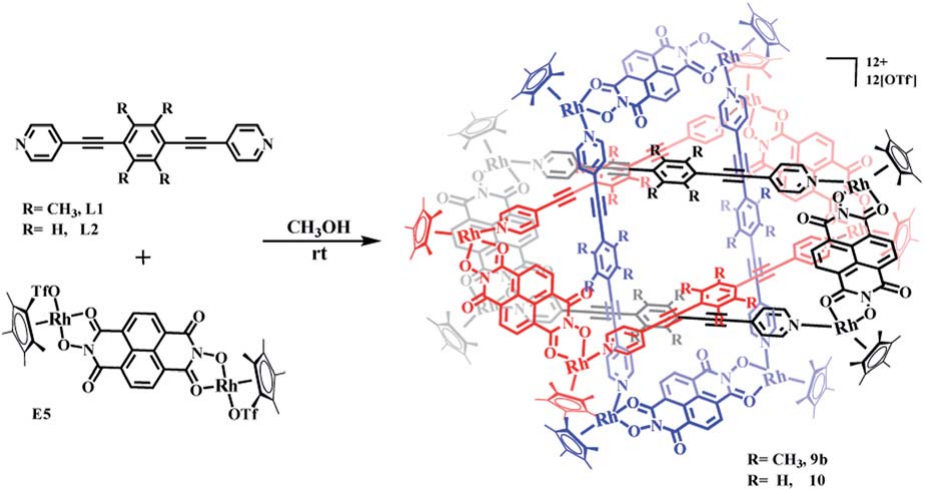
The synthesis of molecular Borromean ring assemblies 9b and 10.

Thereby, the edge unit E5 was separately combined with ligands L1 and L2 in stirred methanol solutions for 8 h at room temperature, at which point evaporation of solvent and recrystallization provided two species as brown solids (yield: 89.5% for 9b and 83.6% for 10) (Scheme 3). The structures of the complexes were confirmed by NMR spectroscopy, elemental analysis and single-crystal X-ray diffraction analysis.

NMR spectroscopic experiments were initially performed to study the solution behavior of 9b, in particular to test whether concentration effects might bring about a structural transformation of 9b. Thus, at a very low concentration in CD_3_OD (0.4 mM), the ^1^H NMR spectrum of 9b showed a pair of doublets and a singlet at 8.66, 7.63 and 2.24 ppm, respectively, attributed to the pyridinyl and methyl group protons of the ligand L1, which was further confirmed by ^1^H–^1^H COSY NMR spectroscopy (Fig. S48†). The resonances for the naphthalenediimide (NDI) and Cp* protons were located at 8.94 and 1.77 ppm. These data suggested the formation of a simple metallarectangle 9a (Fig. S49, S50†). The emergence of new NMR signals and the weakening of the original signals was observed at higher concentrations(0.5–22.0 mM), which is consistent with the aforementioned transformations between metallarectangles 3a, 4a, 5a and 3b, 4b, 5b. At high concentration (22.0 mM), the NMR data suggested formation of a topologically complicated complex (9b), whereby the resonances for the pyridinyl protons of L1 ligand were consistent with a metallarectangle but with slight shifts (8.92, 7.74 ppm). Meanwhile, an obvious shift towards high field and splitting of signals occurred for the methyl protons of L1 ligand at 0.89 and 0.68 ppm. In addition, the NDI proton signal also underwent a significant shift to 8.67 ppm (Fig. S51†). The ^1^H–^1^H DOSY NMR spectrum of 9b showed that the aromatic and Cp* signals are associated with a single diffusion constant (*D* = 7.94 × 10^−11^ m^2^ s^−1^), suggesting that only one stoichiometry of assembly was generated (Fig. S52†). These results clearly suggest the formation of a complicated complex at high concentrations in methanol.

Single crystals of 9b were grown by slow diffusion of diethyl ether vapor into a solution of 9b in methanol and a solid-state structure was derived from X-ray diffraction analysis. Despite many attempts, the crystal data of 9b remained poor, but nevertheless confirm its Borromean ring topology rather than that of a [2]- or [3]catenane (Fig. S1†). The three chemically independent and equivalent rings are locked in such a way that no two of the three rings are linked with each other, each of them adopting a distorted rectangle-like conformation. This result confirmed our hypothesis that the space provided by the constituent units is large enough to avoid the van der Waals interactions and allow π–π stacking interactions to form between the central unit of L1 ligand and the NDI unit of E5 (Fig. S1†).

Changing the concentration or ratio of methanol to DMF has been shown to induce reversible supramolecular transformations between metalla[2]catenanes and the corresponding monorectangles.^53,54^ This structural interconversion might be due to a weakening of the π–π stacking interactions by changing the concentration or the addition of DMF molecules. This inspired us to explore whether the transformation of metalla-Borromean rings could also occur, given the existence of multiple π–π stacking interactions between E5 and central unit of L1. As expected, the metalla-Borromean ring structure 9b can be transformed to the corresponding monorectangle 9a by the dropwise addition of DMF. The ^1^H NMR spectral patterns of 9b in CD_3_OD solution revealed obvious changes as DMF-d_7_ was added gradually. When the ratio of CD_3_OD to DMF-d_7_ reached 5 : 6 (v/v), 9b was nearly completely transformed into 9a. These results further demonstrated that the structural interconversion came from a weakening of the π–π stacking interactions by the DMF molecules (Fig. S54†). Similar phenomena have been described recently.^24,35^ In addition, structural changes induced by concentration effects were also observed (Fig. S53†).

**Fig. 10:**
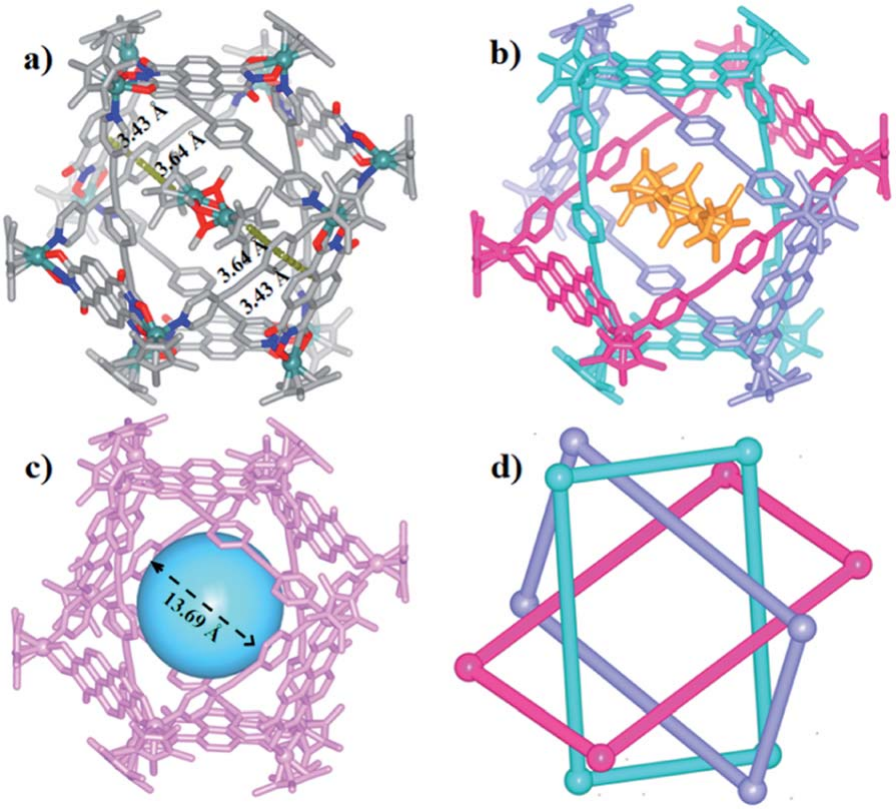
Molecular structure of complex 10. (a) and (b) Representation showing host-guest chemistry and π–π interactions between L2 and the NDI groups of E5; (c) the cavity inside 10; (d) simplified representation; most hydrogen atoms, anions, solvent molecules and disordered elements are omitted for clarity (N, blue; O, red; C, gray; Rh, aqua).

The Borromean ring topology of complex 10 was found to be similar to those of compounds 8 and 9b as judged by single-crystal X-ray diffraction analysis, NMR spectroscopy and elemental analysis (Fig. 10). Each of the three equivalent rings adopted a configuration with dimensions of 11.81 and 20.53 Å (Rh–Rh nonbonding distances). As expected, 10 is stabilized by strong π–π stacking interactions (3.43, 3.52, 3.61 Å) between the phenylene rings of L2 and the NDI unit of E5. Interestingly, the internal cavity of Borromean ring complex 10 contained a dinuclear Rh-based half-sandwich complex, in which the two rhodium atoms are bridged through four methanol oxygen atoms. This structure demonstrated that the two Cp* groups of this guest molecule form stable π–π stacking interactions (3.64 Å) with opposite phenylene groups of the L2 units, respectively, resulting in a stable host–guest complex (Fig. 10a).

The solution behavior of 10 was explored in CD_3_OD solution. Similar to Borromean ring complexes 8 and 9b, the ^1^H NMR spectrum of 10 showed a single, simple set of signals, and these were assigned with help from ^1^H–^1^H COSY and ^1^H–^1^H DOSY spectroscopy (Fig. S55–S57†). No new signals were observed when the concentration in methanol changed from 2.0 mM to 15.0 mM, reflecting the good stability of the Borromean ring complex 10 in methanol, in contrast to 9b.

A careful structural analysis and ^1^H NMR spectroscopic experiment of Borromean assembly 10 reflected that the templating effect played an important role in the formation and stability of 10. From the single crystal structure of 10, two sets of obvious π–π stacking interactions between Cp* group of Cp*Rh dimer and opposite benzene group of ligand L2 could be observed clearly (3.64 Å), possibly stabilizing the existence of this structure better. And the ^1^H NMR spectrum of 10 showed a single, simple set of signals, and no new signals were observed when the concentration in methanol changed from 2.0 mM to 15.0 mM, also reflecting the good stability of the Borromean ring 10 in methanol. Meanwhile, the addition of polar solvents DMF-d_7_ molecules into a CD_3_OD solution of Borromean ring 10 also did not cause Borromean structural change (Fig. S59, S60†). These results demonstrated amply the stability of Borromean ring 10. However, for the Borromean assembly 9b, no Cp*Rh unit was not included in its cavity, possibly resulting from steric hindrance effect of methyl groups, displaying no template effect in the Borromean ring 9b. The ^1^H NMR spectroscopic experiment reflected that the concentration and solvent effects both can induce the structural transformation from simple metallarectangle to the Borromean assembly, showing the weaker structural stability of Borromean ring 9b. These results demonstrated that Cp*Rh dimer played an important role as a template to stabilize the Borromean structure 10.

### Near-infrared photothermal conversion studies

**Fig. 11:**
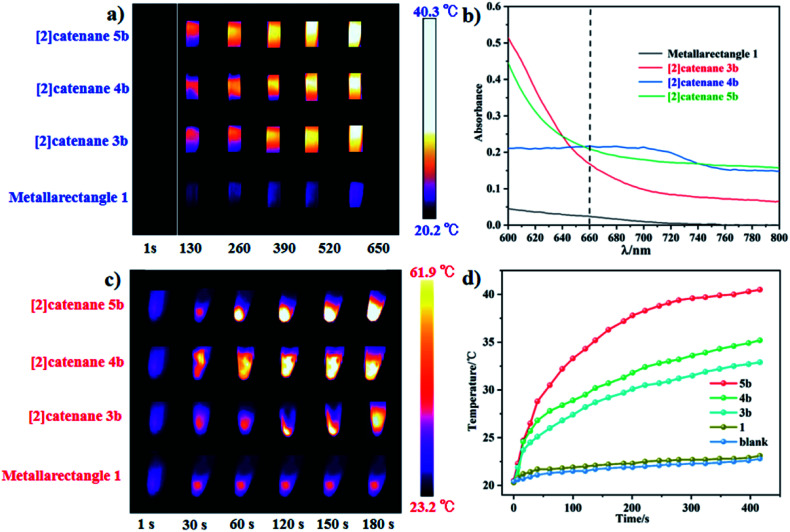
(a) NIR thermal images of four topologies in the spectrophotometer cell (1 × 1 × 5 cm) under 660 nm laser irradiation; (b) absorption in the near-infrared region of the four topologies (800 to 600 nm); (c) NIR photothermal conversion images of crystalline samples of 1, 3b, 4b, 5b under 0.2 W cm^−2^ laser irradiation; (d) photothermal conversion curves of the four topologies.

In our extensive experience with the synthesis of complicated supramolecular topologies, π–π stacking interactions have proven to be a very important factor. These interactions have been suggested to promote nonradiative transitions and to trigger efficient photothermal conversion.^60^ To gain a reliable measure of the influence of π–π stacking in promoting photothermal conversion, we sought to compare compounds of similar compositions but with different extents of π–π interaction. Thereby, the NIR photothermal conversion properties of four topologies based on the L1 ligand were investigated, including tetranuclear metallarectangle 1 (with only a single π–π stacking interaction in the solid state, as shown in Fig. 2) and [2]catenanes 3b, 4b and 5b (with treble π–π stacking interactions, as shown in Fig. 5 and 8). To ensure the same quantity of conjugated phenylene groups, the applied molar ratio of the topologies 1/3b/4b/5b was 2 : 1 : 1 : 1. The resulting near-infrared photothermal conversion images are shown in Fig. 11.

**Fig. 12:**
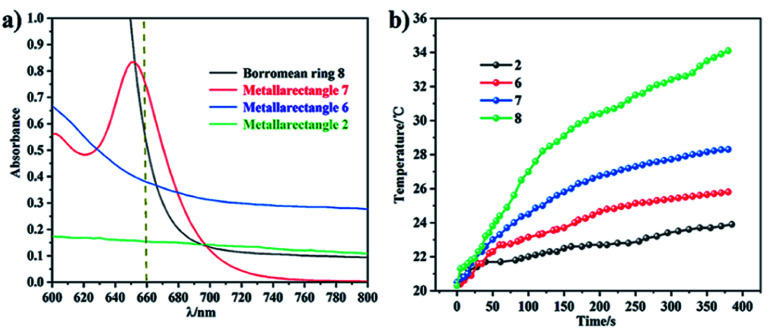
(a) Absorption in the near-infrared region of the four topologies (800 to 600 nm); (b) photothermal conversion curves of the four complexes.

The temperature of a solution of tetranuclear metallarectangle 1 (0.67 mM in CH_3_OH solvent) increased only 2.3 °C (from 20.3 to 23.0 °C, Fig. 12a) under 0.6 W cm^−2^ laser irradiation at a 660 nm wavelength, which was almost equal to the increase of a blank sample (pure CH_3_OH solvent without solute, from 20.4 to 22.9 °C, Fig. 8c). Although a single π–π stacking interaction was found in the solid-state structure of 1, the interaction might not be present in solution due to a lack of sufficient interactions. In addition, for the three [2]catenanes, although they have the same amount of π–π stacking interactions, they have distinct conjugated planes, possibly leading to some differences in photothermal conversion efficiency. As expected, both [2]catenanes 3b and 4b showed a significant increase (from 20.5 to 32.5 °C for 3b, from 20.3 to 33.6 °C for 4b. Fig. 12a, b, d), corresponding to distinct photothermal conversion efficiencies (48.28% for 3b, 60.62% for 4b) (Fig. S63, S64†). A greater temperature difference (20.5 °C) was observed with a solution of [2]catenane 5b which might be due to the stronger conjugated effects of the E4 unit in 5b. According to the equations shown below, a 72.21% efficiency was calculated for 5b (Fig. S65†). From this we can derive a rule of thumb that states that the NIR photothermal conversion efficiency is related not only to the amount of π–π stacking, but also to the conjugated effects of the half-sandwich-based building block units. Firstly, the presence of plentiful π–π stacking interactions promotes nonradiative transitions and triggers photothermal conversion. Secondly, no fluorescence was observed in these compounds when irradiated with 660 nm wavelength light, suggesting very weak radiative transitions. Thus, nonradiative transitions (photothermal conversion) becomes the predominant method of energy release.

Solid photothermal conversion materials have garnered much interest in recent decades, and a range of such solid materials have been studied. Thus, the half-sandwich-based compounds prepared in this work have promise as a new type of advanced materials. The temperature increases of [2]catenanes 3b, 4b, 5b were 25.1 °C (from 22.8 to 47.9 °C), 30.1 °C (from 23.0 to 53.1 °C), and 38.7 °C (from 23.2 to 61.9 °C), respectively, in 180 s under low-power irradiation (0.4 W cm^−2^, Fig. 11c). These results are comparable with other top-performing materials reported elsewhere.^60,61^ This research reflects that our half-sandwich complexes have a more efficient photothermal conversion effect in the solid state than in solution.

In addition, the NIR photothermal conversion of 2, 6, 7 and 8 was explored. When the respective solution was subjected to laser irradiation (0.6 W cm^−2^), 2 and 6 (tetranuclear metallarectangles) showed very weak temperature changes (2.5 °C, 3.1 °C), which are almost indistinguishable from a blank solution without solute (2.2 °C). These results are in line with their structures due to the absence of π–π stacking interactions. In contrast, tetranuclear metallarectangle 7 showed a significant temperature change (10.5 °C), corresponding to a photothermal conversion efficiency of 12.64%, which is ascribed to the large conjugated effect of the building block E3 in 7 (Fig. S66†). Borromean ring assembly 8 showed a large temperature increase (15.5 °C; from 20.5 to 34.1 °C, Fig. 12) under 0.6 W cm^−2^ laser irradiation at 660 nm, corresponding to a 30.53% efficiency and reflecting a strong photothermal conversion effect (Fig. S67†). This result further demonstrates that the strong π–π stacking interaction of the Borromean ring assembly (six sets of π–π stacking interactions) promote nonradiative transitions and trigger efficient photothermal conversion (Fig. 12). These results regarding the photothermal conversion of Borromean ring assemblies are very encouraging for the further applications of complicated topologies and provide a clear rule of thumb whereby the photothermal conversion efficiency is related to the number of π–π stacking interactions and the type of dinuclear building block units.

## Conclusions

In summary, we have demonstrated the selective synthesis and reversible transformation of three kinds of supermolecular topologies, molecular Borromean ring assemblies, [2]catenanes and metallarectangles, based on two alkynyl ligands with/without methyl groups. The results showed that the introduction of methyl groups into the ligand enhanced the π-donor effects but also increase its size, ultimately leading to two distinct topologies: Borromean ring assemblies and [2]catenanes. In addition, π–π stacking interactions play a crucial role in stabilizing the topologies, while also promoting nonradiative transitions and triggering efficient photothermal conversion. Along these lines, the NIR photothermal conversion of these assemblies was studied both in solution and in the solid state. The conversion efficiencies of metallarectangle 7, [2]catenanes 3b, 4b, 5b and Borromean ring assembly 8 were determined to be 12.64%, 48.28%, 60.62%, 72.21% and 30.53%, respectively, in methanol. These data clearly reflect that the photothermal conversion efficiency improves as the number of π–π stacking interactions increases, while it is also dependent on the structure of the half-sandwich building block in the solution state. This research highlights that the introduction of electron-donating groups into the conjugated center has a significant impact on the formation of π–π stacking interactions, leading to the construction of numerous novel and intricate topologies. These results will further inspire the strategic design of topologically complex molecular architectures that were previously inaccessible, and provide promising candidates for materials science applications.

## Data availability

Data for this work, including general experimental procedures, characterization data (NMR and ESI-MS spectra) for all new compounds, X-ray data and near-infrared photothermal conversion research are provided in the ESI.†

## Author contributions

All authors conceptualized the research. L. L. Dang and G. X. Jin conceived the idea. T. T. Li, T. T. Zhang and Y. Zhao executed the work and analysed the data along with L. L. Dang and G. X. Jin. The manuscript was written through contributions of all authors. All authors have given approval to the final version of the manuscript.

## Conflicts of interest

There are no conflicts to declare.

## Supplementary Material

SC-013-D2SC00437B-s001

SC-013-D2SC00437B-s002
