# Supplementary material for: Highly selective synthesis and near-infrared photothermal conversion of metalla-Borromean ring and [2]catenane assemblies
Source: Chem Sci. 2022 Apr 5;13(18):5130–40. doi: 10.1039/d2sc00437b (PMC9093202; doi:10.1039/d2sc00437b)
Supplement: SC-013-D2SC00437B-s001 [file SC-013-D2SC00437B-s001.pdf]

Electronic Supplementary Material (ESI) for Chemical Science.
This journal is © The Royal Society of Chemistry 2022

Supporting information for

Highly selective synthesis and near-infrared photothermal conversion of metalla-Borromean ring and [2]catenane assemblies

Li-Long Dang,*‡[ab] Ting-Ting Li,‡[ac] Ting-Ting Zhang,[a] Ying Zhao,[a] Tian Chen,[a] Xiang Gao,[b] Lu-Fang Ma,[a] and Guo-Xin Jin*[b]

a. College of Chemistry and Chemical Engineering, Henan Province Function-Oriented Porous Materials Key Laboratory, Luoyang Normal University, Luoyang 471934 (P. R. China).

b. Shanghai Key Laboratory of Molecular Catalysis and Innovative Materials, State Key Laboratory of Molecular Engineering of Polymers, Department of Chemistry, Fudan University, Shanghai 200438, P. R. China

c. College of Chemistry and Bioengineering, Guilin University of Technology, Guangxi Key Laboratory of Electrochemical and Magnetochemical Functional Materials, Guilin 541004 (P. R. China).

*E-mail: gxjin@fudan.edu.cn; danglilong8@163.com

Contents

1. **General considerations** 2

2. **Synthesis of complex 1, 2, 3a, 3b, 4a, 4b, 5a, 5b, 6, 7, 8, 9a, 9b and 10** 5

3. **Single-crystal X-ray structures of 9b and 10** 6

4. **NMR spectra** 34

5. **ESI-MS spectra** 36

6. **Near-infrared photothermal conversion research** 39

7. **X-ray crystallography details** 45

8. **References** 46

1. General considerations

All reagents and solvents were purchased from commercial sources and used as supplied unless otherwise mentioned. The starting materials $[Cp^*RhCl_2]_2$ (Cp*=η^5-pentamethylcyclopentadienyl)[1], BiBzIm (BiBzIm = 2, 2′-bisbenzimidazole)[2] were prepared by literature methods. NMR spectra were recorded on Bruker AVANCE I 400 spectrometers at room temperature and referenced to the residual protonated solvent. Proton chemical shifts are reported relative to the solvent residual peak (δ H = 3.31 (CD_3OD), 2.50 (DMSO-D6), 2.75, 2.92 (DMF)) and δC = 49.00 (CD_3OD), 29.76, 34.89 (DMF)). Coupling constants are expressed in Hertz. Elemental analyses were performed on an Elementar Vario EL III analyzer. ESI-MS spectra were recorded on a Micro TOF II mass spectrometer.

2. Synthesis of complex 1, 2, 3a, 3b, 4a, 4b, 5a, 5b, 6, 7, 8, 9a, 9b and 10

Preparation of complex 1

AgOTf (123.2 mg, 0.48 mmol) was added to a solution of $[Cp^*RhCl_2]_2$ (74.4 mg, 0.12 mmol) in a CH_3OH (8 mL) at room temperature. The reaction mixture was stirred in the dark for 24 h and then filtered. BiBzIm (28.1 mg, 0.12 mmol) was added to the filtrate. The mixture was stirred at room temperature for 12 h to give a yellow solution. **L1** (40.4 mg, 0.12 mmol) was then added. The mixture was stirred at room temperature for another 12 h to give a yellow solution. Upon the addition of diethyl ether, a yellow solid was precipitated and collected. The product was recrystallized from a CH_3OH/diethyl ether mixture to afford yellow block-shaped crystals (**1**). 141.83 mg, yield 88.2%. Anal. Calcd for $C_{120}H_{116}N_{12}O_{12}F_{12}S_4Rh_4$ (M = 2686.16): C, 53.66; H, 4.35; N, 6.26. Found: C, 53.46; H, 4.30; N, 6.28. ^1H NMR (400 MHz, CD_3CD, ppm, with respect to Cp*Rh): δ = 8.09 (m, 4H, BiBzIm-aH), δ = 7.76 (d, J = 5.6Hz, 4H, pyridyl-cH), δ= 7.55 (m, 4H, BiBzIm-bH), δ = 6.98 (d, J = 5.6Hz, 4H, pyridyl-dH), δ = 2.14 (s, 12H, $-CH_3$), δ = 1.91 (s, 30H, Cp*-H).

Preparation of complex 2

AgOTf (123.2 mg, 0.48 mmol) was added to a solution of $[Cp^*RhCl_2]_2$ (74.4 mg, 0.12 mmol) in CH_3OH (8 mL) at room temperature. The reaction mixture was stirred in the dark for 24 h and then filtered. BiBzIm (28.1 mg, 0.12mmol) was added to the filtrate. The mixture was stirred at room temperature for 12 h to give a yellow solution. **L2** (33.64 mg, 0.12 mmol) was then added. The mixture was stirred at room temperature for another 12 h to give a yellow solution. Upon the addition of diethyl ether, a yellow solid was precipitated and collected. The product was recrystallized from a CH_3OH/diethyl ether mixture to afford block-shaped crystals (**2**). 128.18 mg, yield: 83.4%. Anal. Calcd for $C_{112}H_{100}N_{12}O_{12}F_{12}S_4Rh_4$ (M = 2573.95): C, 52.26; H, 3.92; N, 6.53. Found: C, 52.21; H, 3.89; N, 6.55. ^1H NMR (400 MHz, CD_3CD, ppm, with respect to Cp*Rh): δ = 8.09 (m, 4H, BiBzIm-aH), δ = 7.77 (d, J = 5.6Hz, 4H, pyridyl-cH), δ= 7.57 (m, 4H, BiBzIm-bH), δ = 6.97 (d, J = 6.0 Hz, 4H, pyridyl-dH), δ = 1.90 (s, 30H, Cp*-H).

Preparation of complex 3a

AgOTf (123.2 mg, 0.48 mmol) was added to a solution of $[Cp^*RhCl_2]_2$ (74.4 mg, 0.12 mmol) in the mixture solution of CH_3OH (4 mL) and DMF (16 mL) at room temperature. The reaction mixture was stirred in the dark for 12 h and then filtered. 2,5-Dihydroxy-1,4-benzoquinone (16.8 mg, 0.12 mmol) and NaOH (9.6 mg, 0.24 mmol) was added to the filtrate. The mixture was stirred at room temperature for 12 h to give a dark brown solution. **L1** (40.4 mg, 0.12 mmol) was then added. The mixture was stirred at room temperature for another 12 h to give a dark brown solution. The solvent was concentrated to about 8 mL. Upon addition of diethyl ether, a dark brown solid was precipitated and collected. The product was recrystallized from a methanol/diethyl ether mixture to afford block-shaped crystals (**3a**). 132.63 mg, yield: 88.5%. Anal. Calcd for

$C_{104}H_{104}F_{12}N_4O_{20}Rh_4S_4$ (M = 2497.83): C, 50.01; H, 4.20; N, 2.24. Found: C, 50.05; H, 4.17; N, 2.27. ^{1}H NMR (400 MHz, CD_3CD, ppm, with respect to Cp*Rh): δ = 8.28 (d, J = 5.2Hz, 4H, pyridyl-aH), δ = 7.59 (d, J = 5.2Hz, 4H, pyridyl-bH), δ = 5.67 (s, 2H, phenyl-H of E2), δ = 2.34 (s, 12H, $-CH_3$), δ = 1.70 (s, 30H, Cp*-H).

Preparation of complex 3b

AgOTf (123.2 mg, 0.48 mmol) was added to a solution of $[Cp*RhCl_2]_2$ (74.4 mg, 0.12 mmol) in CH_3OH (20 mL) at room temperature. The reaction mixture was stirred in the dark for 12 h and then filtered. 2,5-Dihydroxy-1,4-benzoquinone (16.8 mg, 0.12 mmol) and NaOH (9.6 mg, 0.24 mmol) was added to the filtrate. The mixture was stirred at room temperature for 12 h to give a dark brown solution. **L1** (40.4 mg, 0.12 mmol) was then added. The mixture was stirred at room temperature for another 12 h to give a dark brown solution. The solvent was concentrated to about 8 mL. Upon addition of diethyl ether, a dark brown solid was precipitated and collected. The product was recrystallized from a methanol/diethyl ether mixture to afford block-shaped crystals (**3b**). 135.63 mg, yield: 90.5%. Anal. Calcd for $C_{208}H_{208}F_{24}N_8O_{40}Rh_8S_8$ (M = 4995.65): C, 50.01; H, 4.20; N, 2.24. Found: C, 50.03; H, 4.24; N, 2.26. ^{1}H NMR (400 MHz, CD_3CD, ppm, with respect to Cp*Rh): δ = 8.42 (d, J = 4.8Hz, 8H, pyridyl-a1H), δ = 8.34 (d, J = 4.8Hz, 8H, pyridyl-b1H), δ = 7.62 (d, 8H, pyridyl-a2H), δ = 7.58 (d, J = 4.8Hz, 8H, pyridyl-b2H), δ = 5.78 (s, 8H, phenyl-H of E2), δ = 2.10 (s, 48H, -CH_3), δ = 1.74 (d, J = 4.4Hz, 60H, Cp*-H), δ = 1.69 (d, J = 6Hz, 60H, Cp*-H).

Preparation of complex 4a

AgOTf (123.2 mg, 0.48 mmol) was added to a solution of $[Cp*RhCl_2]_2$ (74.4 mg, 0.12 mmol) in the mixture solution of CH_3OH (4 mL) and DMF (16 mL) at room temperature. The reaction mixture was stirred in the dark for 12 h and then filtered. 5,8-Dihydroxy-1,4-naphthoquinone (22.8 mg, 0.12 mmol) and NaOH (9.6 mg, 0.24 mmol) was added to the filtrate. The mixture was stirred at room temperature for 12 h to give a dark green solution. **L1** (40.4 mg, 0.12 mmol) was then added. The mixture was stirred at room temperature for another 12 h to give a dark green solution. The solvent was concentrated to about 8 mL. Upon addition of diethyl ether, a dark green solid was precipitated and collected. The product was recrystallized from a methanol/diethyl ether mixture to afford block-shaped crystals (**4a**). 124.70 mg, yield: 80%. Anal. Calcd for $C_{112}H_{108}N_4O_{20}F_{12}S_4Rh_4$ (M = 2597.95): C, 51.78; H, 4.19; N, 2.16. Found: C, 51.73; H, 4.15; N, 2.11.

Preparation of complex 5a

AgOTf (123.2 mg, 0.48 mmol) was added to a solution of $[Cp*RhCl_2]_2$ (74.4 mg, 0.12 mmol) in the mixture solution of CH_3OH (4 mL) and DMF (16 mL) at room temperature. The reaction mixture was stirred in the dark for 12 h and then filtered. 6,11-Dihydroxy-5,12-naphthacene dione (34.8 mg, 0.12 mmol) and NaOH (9.6 mg, 0.24 mmol) was added to the filtrate. The mixture was stirred at room temperature for 12 h to give a dark red solution. **L1** (40.4 mg, 0.12 mmol) was then added. The mixture was stirred at room temperature for another 12 h to give a green red solution. The solvent was concentrated to about 8 mL. Upon addition of diethyl ether, a dark green solid was precipitated and collected. The product was recrystallized from a methanol/diethyl ether mixture to afford block-shaped crystals (**5a**). 146.07 mg, yield: 87%. Anal. Calcd for $C_{128}H_{116}N_4O_{20}F_{12}S_4Rh_4$ (M = 2798.19): C, 54.94; H, 4.18; N, 2.00. Found: C, 54.90; H, 4.15; N, 2.05. ^{1}H NMR (400 MHz, CD_3CD, ppm, with respect to Cp*Rh): δ = 8.74 (d, J = 7.2Hz, 8H, phenyl-cH of E4), δ = 8.46 (d, J = 5.2Hz, 8H, pyridyl-aH), δ = 7.96 (d, J = 7.6Hz, 8H, phenyl-dH of E4), δ = 7.38 (d, J = 2.8Hz, 8H, pyridyl-bH), δ = 2.19 (s, 24H, $-CH_3$), δ = 1.75 (s, 60H, Cp*-H).

Preparation of complex 5b

AgOTf (123.2 mg, 0.48 mmol) was added to a solution of $[Cp*RhCl_2]_2$ (74.4 mg, 0.12 mmol) in CH_3OH (20 mL) at room temperature. The reaction mixture was stirred in the dark for 12 h and then filtered. 6,11-Dihydroxy-5,12-naphthacene dione (34.8 mg, 0.12 mmol) and NaOH (9.6 mg, 0.24 mmol) was added to the

filtrate. The mixture was stirred at room temperature for 12 h to give a dark green solution. **L1** (40.4 mg, 0.12 mmol) was then added. The mixture was stirred at room temperature for another 12 h to give a dark green solution. The solvent was concentrated to about 8 mL. Upon addition of diethyl ether, a dark green solid was precipitated and collected. The product was recrystallized from a methanol/diethyl ether mixture to afford block-shaped crystals (**5b**). 151.10 mg, yield: 90.0%. Anal. Calcd for $C_{256}H_{232}N_8O_{40}F_{24}S_8Rh_8$ (M = 5596.37): C, 54.94; H, 4.18; N, 2.00. Found: C, 54.89; H, 4.13; N, 2.10. ^{1}H NMR (400 MHz, CD_3CD, ppm, with respect to Cp*Rh): δ = 8.81 (m, 16H, phenyl-cH of E4), δ = 8.55 (d, J = 4.8Hz, 16H, pyridyl-aH), δ = 8.01 (m, 16H, phenyl-dH of E4), δ = 7.38 (d, J = 4Hz, 16H, pyridyl-bH), δ = 1.96 (s, 24H, $-CH_3$), δ = 1.76 (s, 120H, Cp*-H).

Preparation of complex 6

AgOTf (123.2 mg, 0.48 mmol) was added to a solution of $[Cp^*RhCl_2]_2$ (74.4 mg, 0.12 mmol) in CH_3OH (10 mL) at room temperature. The reaction mixture was stirred in the dark for 12 h and then filtered. 2,5-Dihydroxy-1,4-benzoquinone (16.8 mg, 0.12 mmol) and NaOH (9.6 mg, 0.24 mmol) was added to the filtrate. The mixture was stirred at room temperature for 12 h to give a dark green solution. **L2** (33.64 mg, 0.12 mmol) was then added. The mixture was stirred at room temperature for another 12 h to give a dark green solution. The solvent was concentrated to about 6 mL. Upon addition of diethyl ether, a dark green solid was precipitated and collected. The product was recrystallized from a methanol/diethyl ether mixture to afford block-shaped crystals (**6**). 122.10 mg, yield: 85.3%. Anal. Calcd for $C_{96}H_{88}N_4O_{20}F_{12}S_4Rh_4$ (M = 2385.61): C, 48.33; H, 3.72; N, 2.35. Found: C, 48.25; H, 3.75; N, 2.33. ^{1}H NMR (400 MHz, CD_3CD, ppm, with respect to Cp*Rh): δ = 8.33 (d, J = 4.8Hz, 8H, pyridyl-aH), δ = 7.61 (d, J = 4.8Hz, 8H, pyridyl-bH), δ = 7.55 (d, J = 4.8Hz, 8H, -CH), δ = 5.68 (s, 4H, phenyl-cH of E2), δ = 1.68 (s, 60H, Cp*-H).

Preparation of complex 7

AgOTf (123.2 mg, 0.48 mmol) was added to a solution of $[Cp^*RhCl_2]_2$ (74.4 mg, 0.12 mmol) in CH_3OH (20 mL) at room temperature. The reaction mixture was stirred in the dark for 12 h and then filtered. 5,8-Dihydroxy-1,4-naphthoquinone (22.8 mg, 0.12 mmol) and NaOH (9.6 mg, 0.24 mmol) was added to the filtrate. The mixture was stirred at room temperature for 12 h to give a dark green solution. **L2** (33.64 mg, 0.12 mmol) was then added. The mixture was stirred at room temperature for another 12 h to give a dark green solution. The solvent was concentrated to about 6 mL. Upon addition of diethyl ether, a dark green solid was precipitated and collected. The product was recrystallized from a methanol/diethyl ether mixture to afford block-shaped crystals (**7**). 122.89 mg, yield: 82.4%. Anal. Calcd for $C_{104}H_{92}N_4O_{20}F_{12}S_4Rh_4$ (M = 2485.73): C, 50.25; H, 3.73; N, 2.25. Found: C, 50.15; H, 3.70; N, 2.30. ^{1}H NMR (400 MHz, CD_3CD, ppm, with respect to Cp*Rh): δ = 8.43 (d, J = 5.2Hz, 8H, pyridyl-aH), δ = 7.55 (d, J = 5.2Hz, 8H, pyridyl-bH), δ = 7.51 (s, 8H, -CH), δ = 7.21 (s, 8H, phenyl-H of E3), δ = 1.61 (s, 60H, Cp*-H).

Preparation of complex 8

AgOTf (123.2 mg, 0.48 mmol) was added to a solution of $[Cp^*RhCl_2]_2$ (74.4 mg, 0.12 mmol) in CH_3OH (20 mL) at room temperature. The reaction mixture was stirred in the dark for 12 h and then filtered. 6,11-Dihydroxy-5,12-naphthacene dione (34.8 mg, 0.12 mmol) and NaOH (9.6 mg, 0.24 mmol) was added to the filtrate. The mixture was stirred at room temperature for 12 h to give a dark red solution. **L2** (33.64 mg, 0.12 mmol) was then added. The mixture was stirred at room temperature for another 12 h to give a dark red solution. The solvent was concentrated to about 6 mL. Upon addition of diethyl ether, a dark red solid was precipitated and collected. The product was recrystallized from a methanol/diethyl ether mixture to afford block-shaped crystals (**8**). 129.73 mg, yield: 80.5%. Anal. Calcd for $C_{120}H_{100}N_4O_{20}F_{12}S_4Rh_4$ (M = 2685.97): C, 53.66; H, 3.75; N, 2.09. Found: C, 53.56; H, 3.70; N, 2.05. ^{1}H NMR (400 MHz, CD_3CD, ppm, with respect to Cp*Rh): δ = 8.95 (d, J = 5.2Hz, 8H, pyridyl-aH), δ = 8.60 (d, J = 4.8Hz, 4H, phenyl-cH of E4), δ = 7.62 (m,

4H, phenyl-dH of E4), δ = 7.52 (d, 8H, pyridyl-bH), δ = 4.96 (s, 8H, -CH), δ = 1.74 (s, 60H, Cp*-H).

Preparation of complex 9a

AgOTf (123.2 mg, 0.48 mmol) was added to a solution of $[Cp^*RhCl_2]_2$ (74.4 mg, 0.12 mmol) in the mixture solution of CH_3OH (4 mL) and DMF (16 mL) at room temperature. The reaction mixture was stirred in the dark for 12 h and then filtered. Naphthalenediimide (35.8 mg, 0.12 mmol) and NaOH (9.6 mg, 0.24 mmol) was added to the filtrate. The mixture was stirred at room temperature for 12 h to give a dark brown solution. **L1** (40.4 mg, 0.12 mmol) was then added. The mixture was stirred at room temperature for another 12 h to give a dark brown solution. The solvent was concentrated to about 6 mL. Upon addition of diethyl ether, a dark brown solid was precipitated and collected. The product was recrystallized from a methanol/diethyl ether mixture to afford block-shaped crystals (**9a**). 148.58 mg, yield: 88.6%. Anal. Calcd for $C_{120}H_{108}N_8O_{24}F_{12}S_4Rh_4$ (M = 2814.06): C, 51.22; H, 3.87; N, 3.98. Found: C, 51.18; H, 3.83; N, 3.95. ^1H NMR (400 MHz, CD_3CD, ppm, with respect to Cp*Rh): δ = 8.94 (4H, phenyl-c^{1}H of E5), δ = 8.68 (4H, pyridyl-aH), δ = 7.66 (4H, pyridyl-bH), δ = 2.26 (m, 12H, $-CH_3$), δ = 1.79 (s, 30H, Cp*-H).

Preparation of complex 9b

AgOTf (123.2 mg, 0.48 mmol) was added to a solution of $[Cp^*RhCl_2]_2$ (74.4 mg, 0.12 mmol) in CH_3OH (20 mL) at room temperature. The reaction mixture was stirred in the dark for 12 h and then filtered. Naphthalenediimide (35.8 mg, 0.12 mmol) and NaOH (9.6 mg, 0.24 mmol) was added to the filtrate. The mixture was stirred at room temperature for 12 h to give a dark brown solution. **L1** (40.4 mg, 0.12 mmol) was then added. The mixture was stirred at room temperature for another 12 h to give a dark brown solution. The solvent was concentrated to about 6 mL. Upon addition of diethyl ether, a dark brown solid was precipitated and collected. The product was recrystallized from a methanol/diethyl ether mixture to afford block-shaped crystals (**9b**). 151.12 mg, yield: 89.5%. Anal. Calcd for $C_{360}H_{324}N_{24}O_{72}F_{36}S_{12}Rh_{12}$ (M = 8442.18): C, 51.22; H, 3.87; N, 3.98. Found: C, 51.15; H, 3.85; N, 3.95. ^1H NMR (400 MHz, CD_3CD, ppm, with respect to Cp*Rh): δ = 8.92 (4H, pyridyl-aH), δ = 8.67 (4H, phenyl-c1H of E5), δ = 8.27 (4H, phenyl-c2H of E5), δ = 7.74 (4H, pyridyl-bH), δ = 0.80 (m, 12H, $-CH_3$), δ = 1.84 (s, 30H, Cp*-H).

Preparation of complex 10

AgOTf (123.2 mg, 0.48 mmol) was added to a solution of $[Cp^*RhCl_2]_2$ (86.8 mg, 0.14 mmol) in CH_3OH (20 mL) at room temperature. The reaction mixture was stirred in the dark for 12 h and then filtered. Naphthalenediimide (35.8 mg, 0.12 mmol) and NaOH (9.6 mg, 0.24 mmol) was added to the filtrate. The mixture was stirred at room temperature for 12 h to give a dark brown solution. **L2** (33.64 mg, 0.12 mmol) was then added. The mixture was stirred at room temperature for another 12 h to give a dark brown solution. The solvent was concentrated to about 6 mL. Upon addition of diethyl ether, a dark brown solid was precipitated and collected. The product was recrystallized from a methanol/diethyl ether mixture to afford block-shaped crystals (**10**). 146.73 mg, yield: 85.5%. Anal. Calcd for $C_{350}H_{318}N_{24}O_{76}F_{36}S_{12}Rh_{14}$ (M = 8580.46): C, 48.96; H, 3.73; N, 3.92. Found: C, 48.92; H, 3.79; N, 3.88. ^1H NMR (500 MHz, CD_3CD, ppm, with respect to Cp*Rh): δ = 8.76 (4H, phenyl-cH of E5), δ = 8.66 (d, 4H, J=6, pyridyl-aH), δ = 7.63 (d, 4H, J=6.5, pyridyl-bH), δ = 7.54 (s, 4H, phenyl-dH of L2), δ = 3.35 (m, 1H, $-CH_3$-gH of guest $-CH_3O$), δ = 1.76 (s, 30H, Cp*-eH), δ = 1.63 (s, 5H, Cp*-fH).

3. Single-crystal X-ray structures of 9b and 10

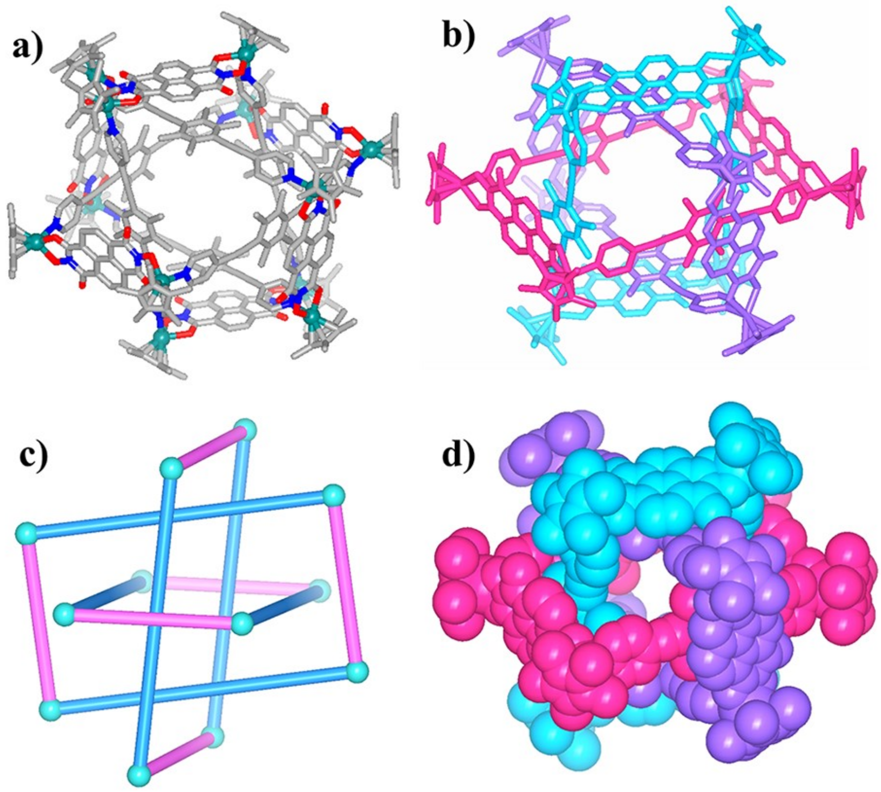

Fig. S1. Molecular structure of complex **9b**. (a) and (b) Representation showing π–π interactions between **L1** and the NDI groups of **E5**; (c) and (d) simplified representation and space-filling; most hydrogen atoms, anions, solvent molecules and disordered elements are omitted for clarity (N, blue; O, red; C, gray; Rh, Aqua).

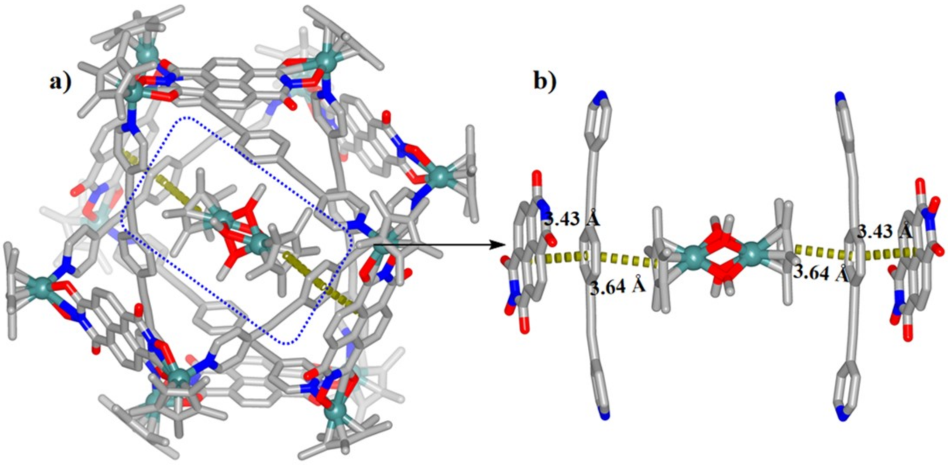

Fig. S2. Molecular structure of complex **10**. a) The host-guest chemistry of Borromean ring **10**; b) Representation showing π–π interactions among NDI group, **L1** and Cp* group of the guest molecule. Most hydrogen atoms, anions, solvent molecules and disordered elements are omitted for clarity (N, blue; O, red; C, gray; Rh, Aqua).

4. NMR Spectra

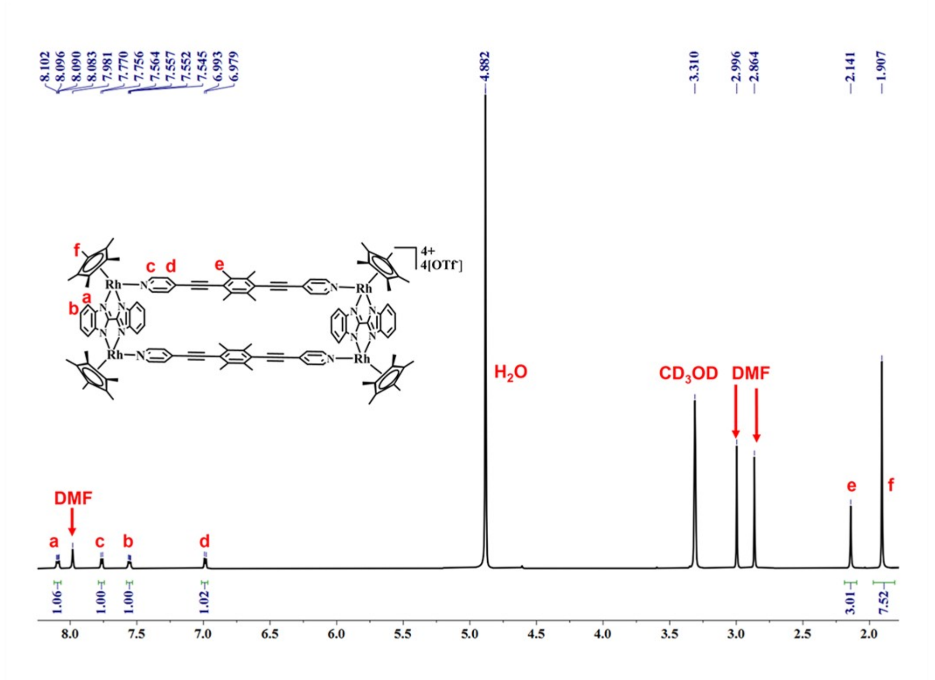

Fig. S3. The ^{1}H NMR (500 MHz, CD_3OD, ppm) for **1** (15.0 mM, with respect to Cp*Rh)

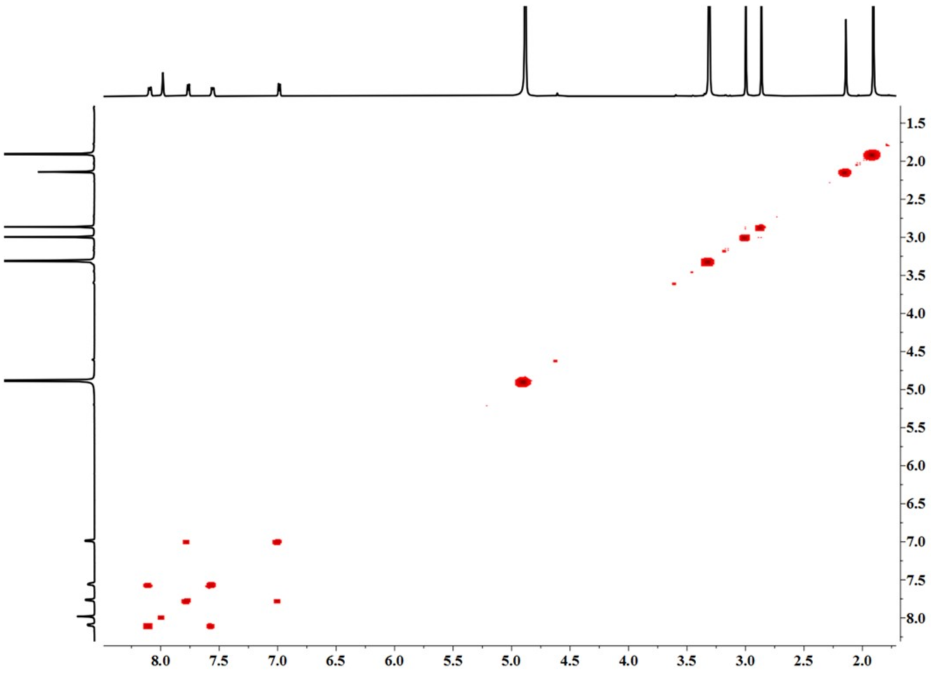

Fig. S4. The ^{1}H-^{1}H COSY NMR (500 MHz, CD_3OD, ppm) for **1** (15.0 mM, with respect to Cp*Rh)

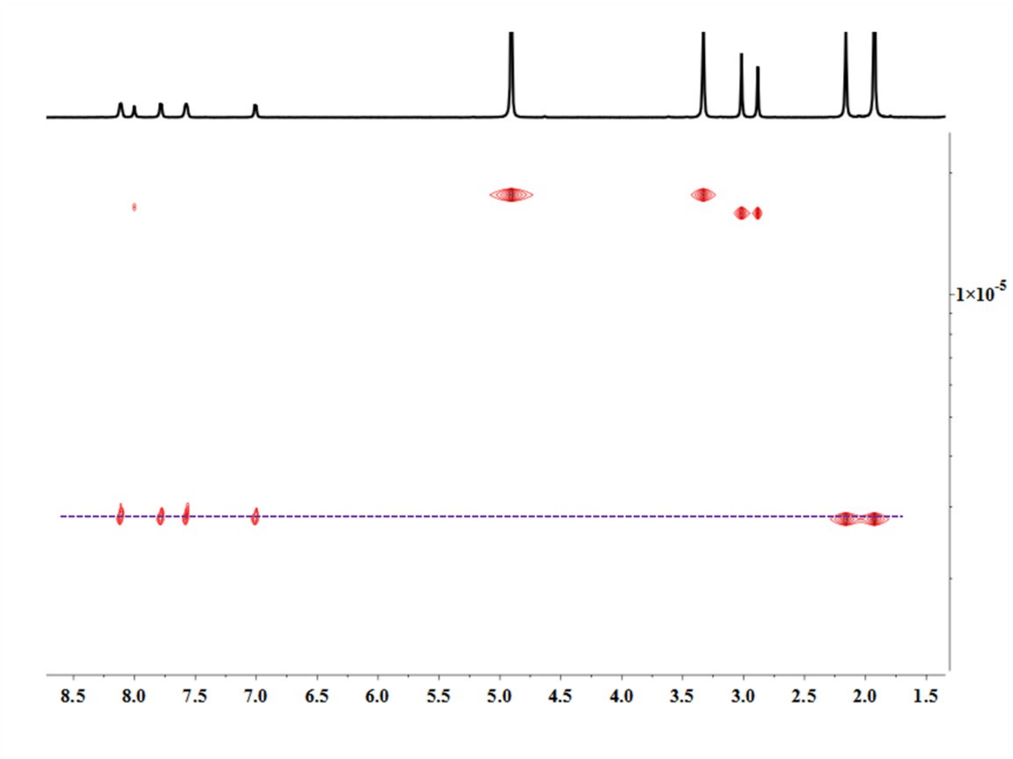

Fig. S5. The ^{1}H-^{1}H DOSY NMR (500 MHz, CD_3OD, ppm) for **1** (2.81×10^{-10} m^2s^{-1}) (15.0 mM, with respect to Cp*Rh)

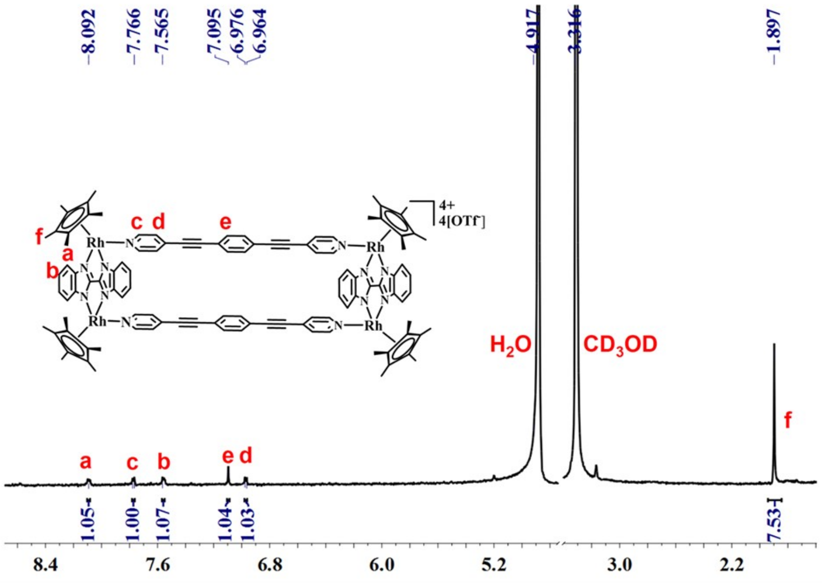

Fig. S6. The ^{1}H NMR (500 MHz, CD_3OD, ppm) for **2** (0.40 mM, with respect to Cp*Rh)

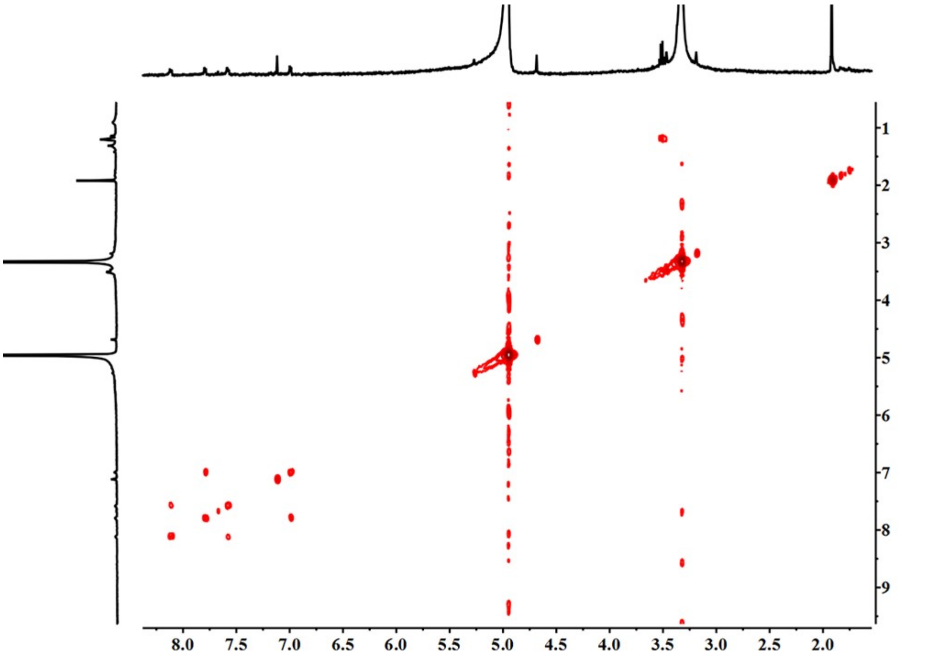

Fig. S7. The ^{1}H-^{1}H COSY NMR (500 MHz, CD_3OD, ppm) for **2** (0.40 mM, with respect to Cp*Rh)

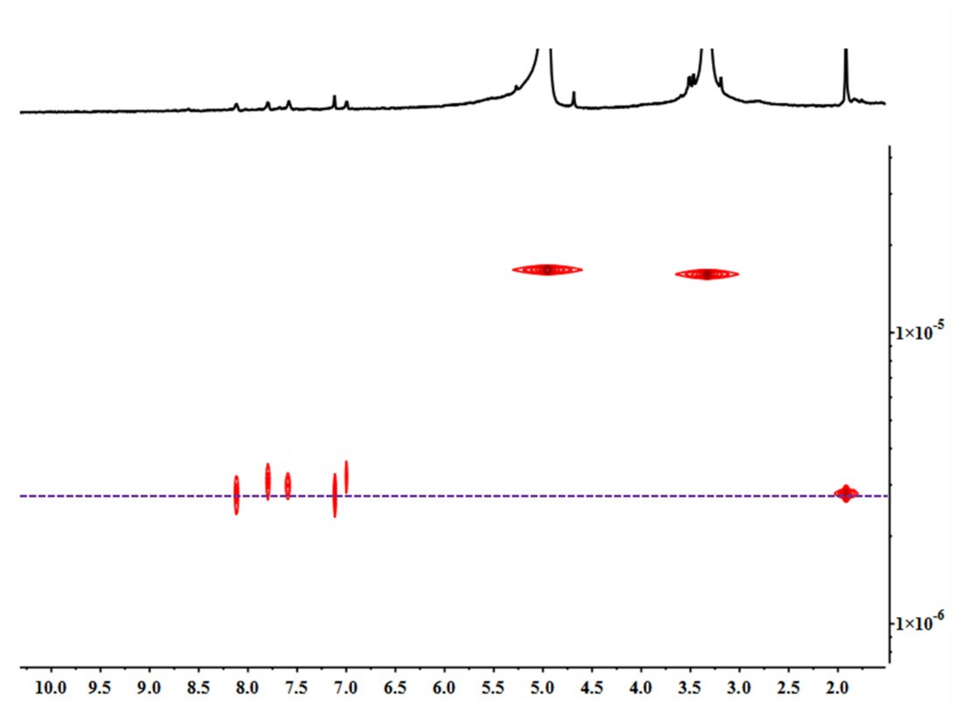

Fig. S8. The ^{1}H-^{1}H DOSY NMR (500 MHz, CD_3OD, ppm) for **2** (2.49×10^{-10} m^2s^{-1}) (0.40 mM, with respect to Cp*Rh)

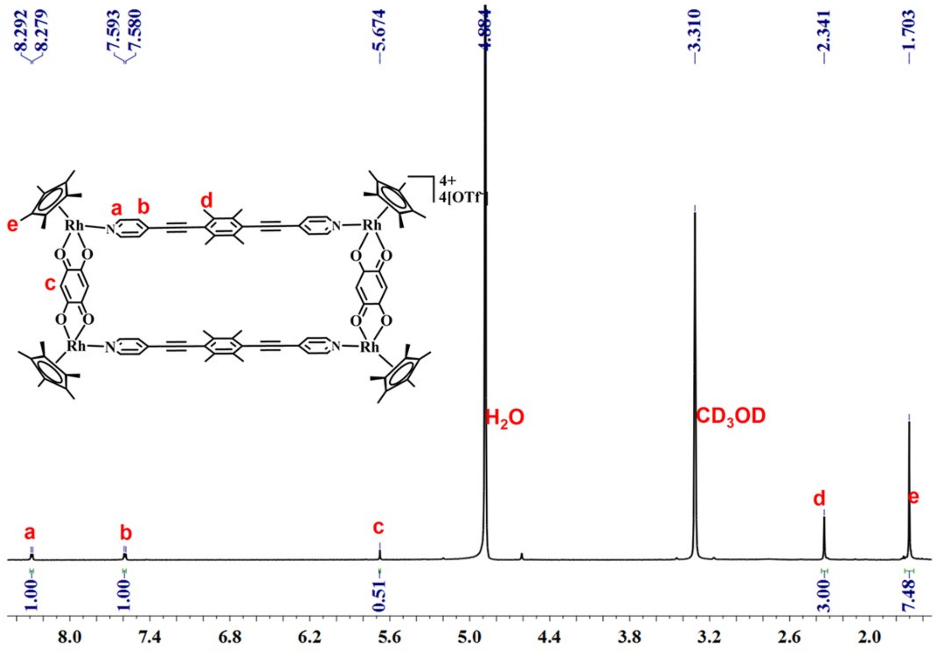

Fig. S9. The ^{1}H NMR (500 MHz, CD_3OD, ppm) for **3a** (1.0 mM, with respect to Cp*Rh)

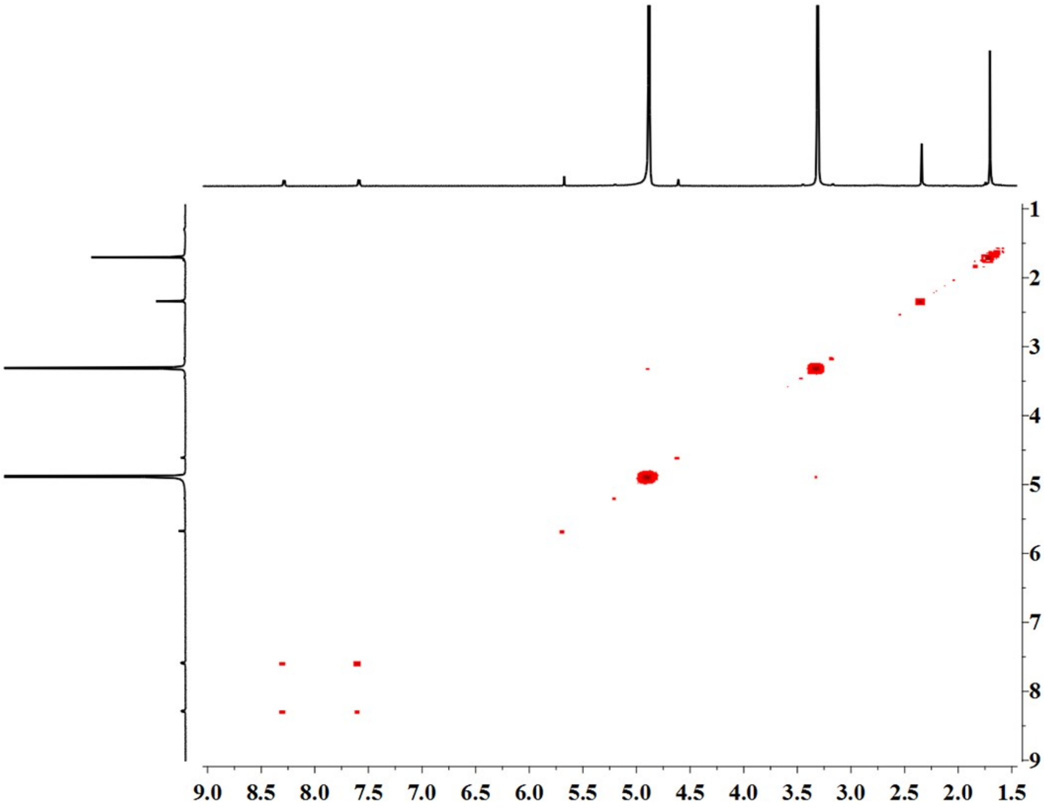

Fig. S10. The ^{1}H-^{1}H COSY NMR (500 MHz, CD_3OD, ppm) for **3a** (1 mM, with respect to Cp*Rh)

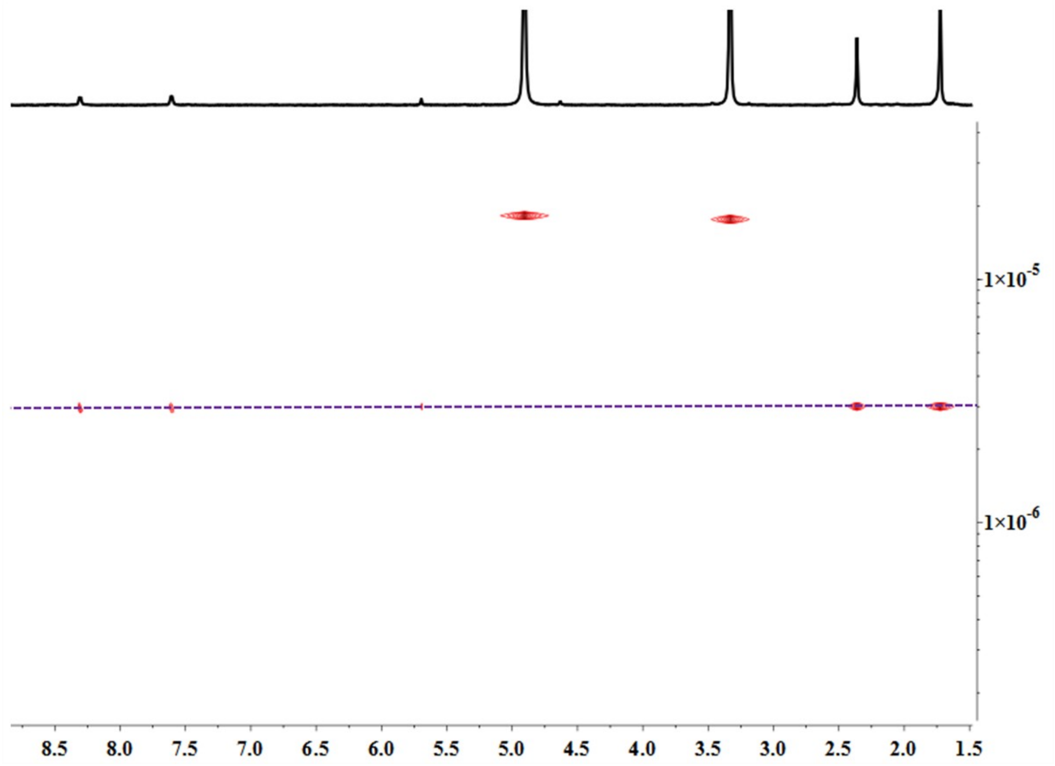

Fig. S11. The ^{1}H-^{1}H DOSY NMR (500 MHz, CD_3OD, ppm) for **3a** (2.94×10^{-10} m^2s^{-1}) (1.0 mM, with respect to Cp*Rh)

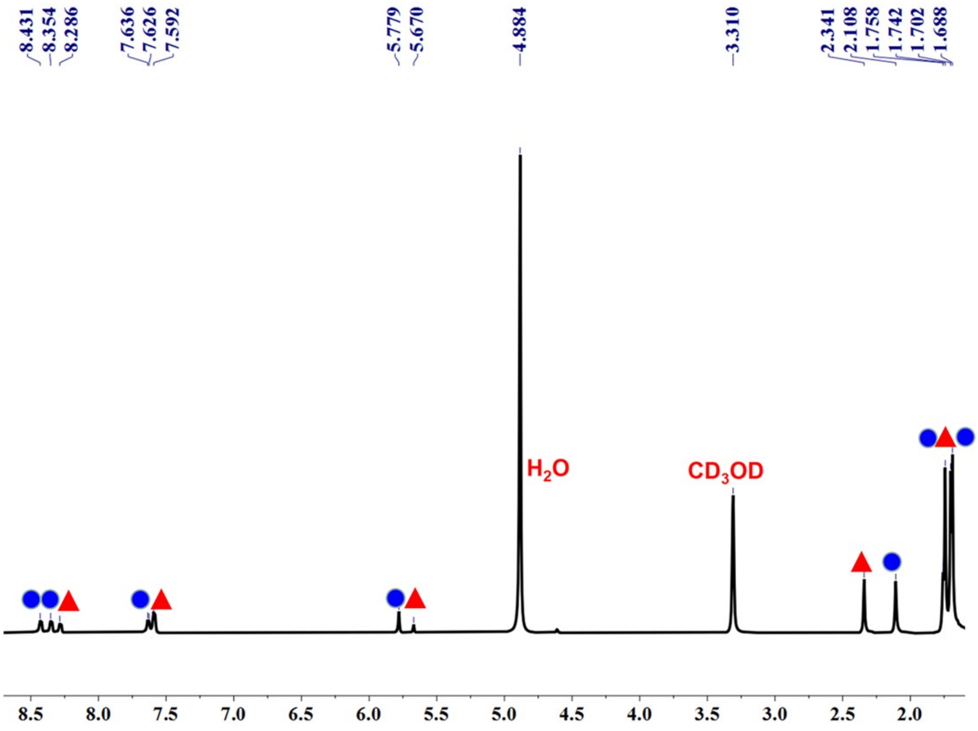

Fig. S12. The ^{1}H NMR (500 MHz, CD_3OD, ppm) for **3a** and **3b** (7.0 mM, with respect to Cp*Rh)

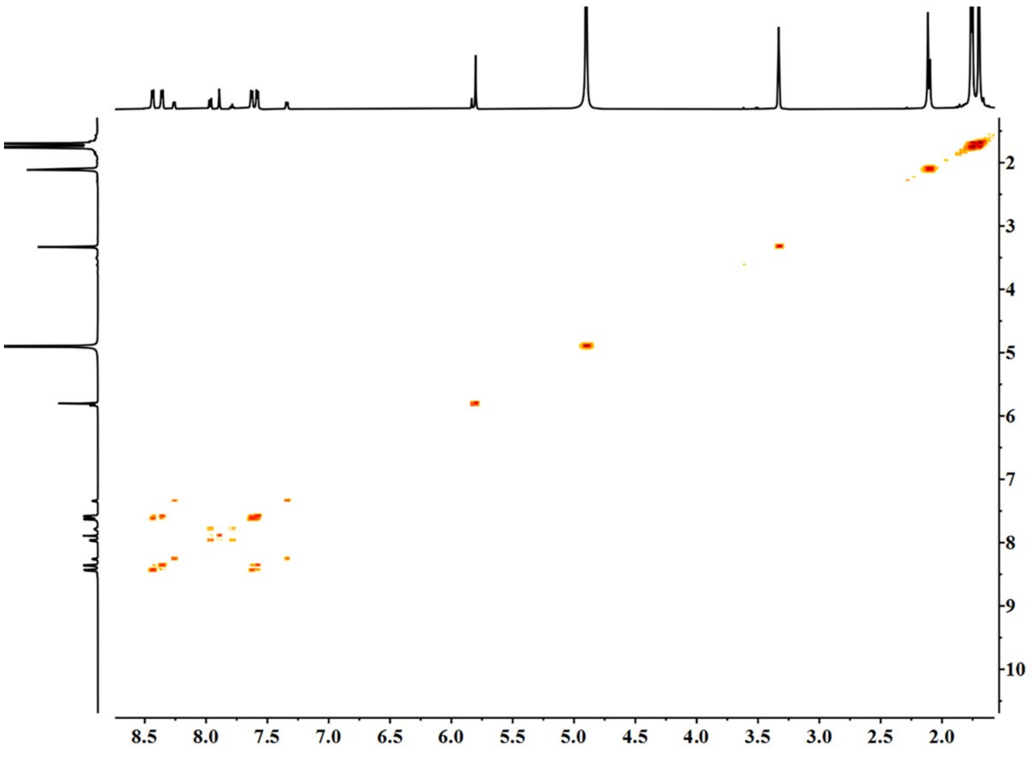

Fig. S13. The ^{1}H-^{1}H COSY NMR (500 MHz, CD_3OD, ppm) for **3a** and **3b** (7.0 mM, with respect to Cp*Rh)

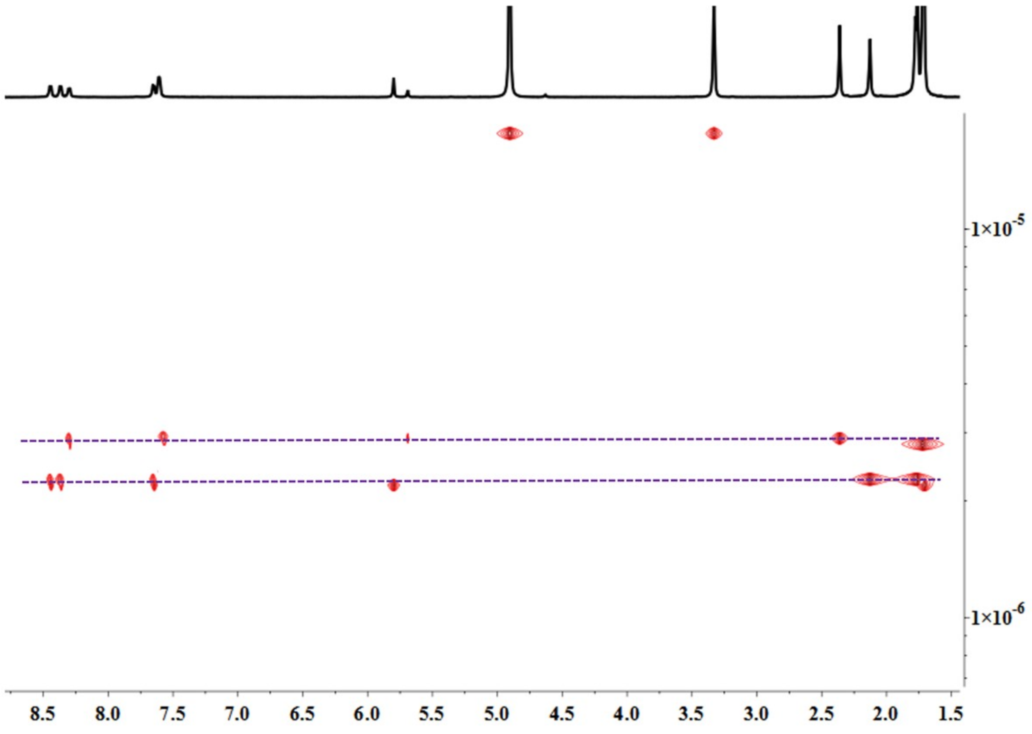

Fig. S14. The ^{1}H-^{1}H DOSY NMR (500 MHz, CD_3OD, ppm) for **3a** (2.73×10^{-10} m^2 s^{-1}) and **3b** (2.42×10^{-10} m^2s^{-1}) (7.0 mM, with respect to Cp*Rh)

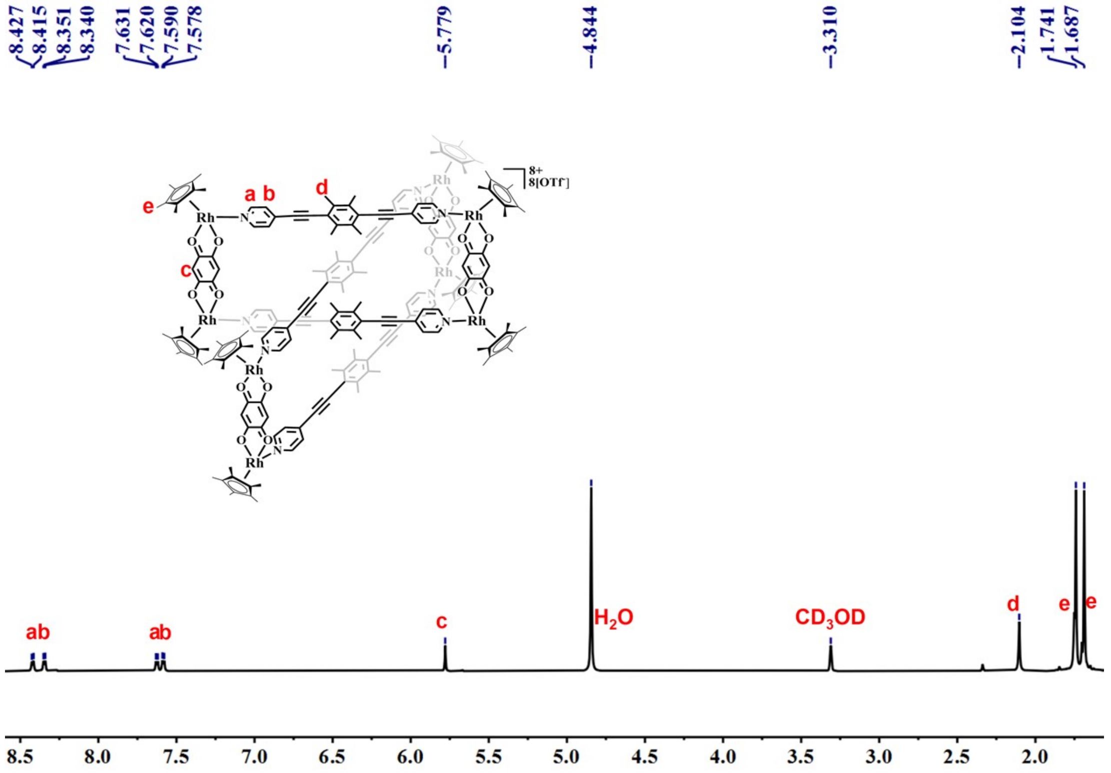

Fig. S15. The ^{1}H NMR (500 MHz, CD_3OD, ppm) for **3b** (20.0 mM, with respect to Cp*Rh)

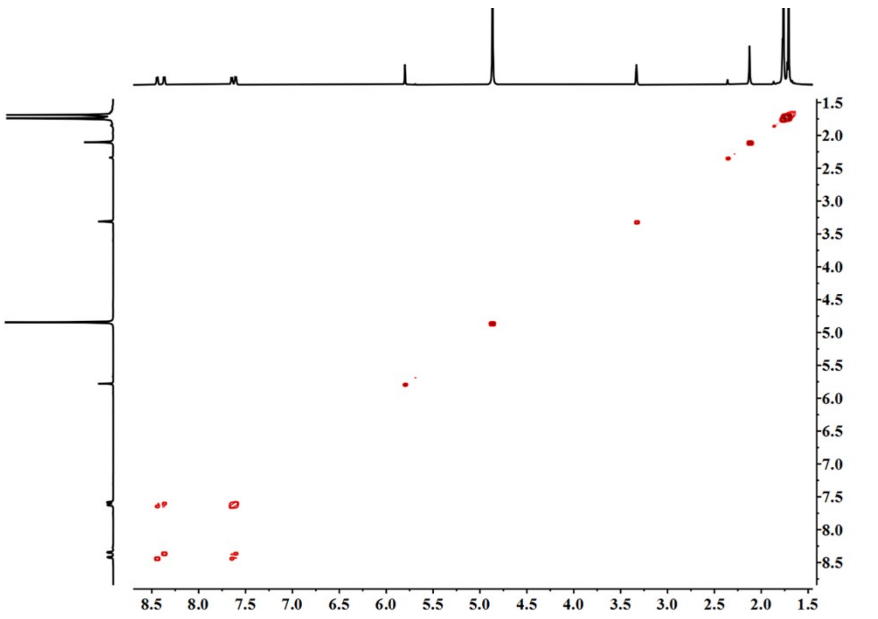

Fig. S16. The ^{1}H-^{1}H COSY NMR (500 MHz, CD_3OD, ppm) for **3b** (20.0 mM, with respect to Cp*Rh)

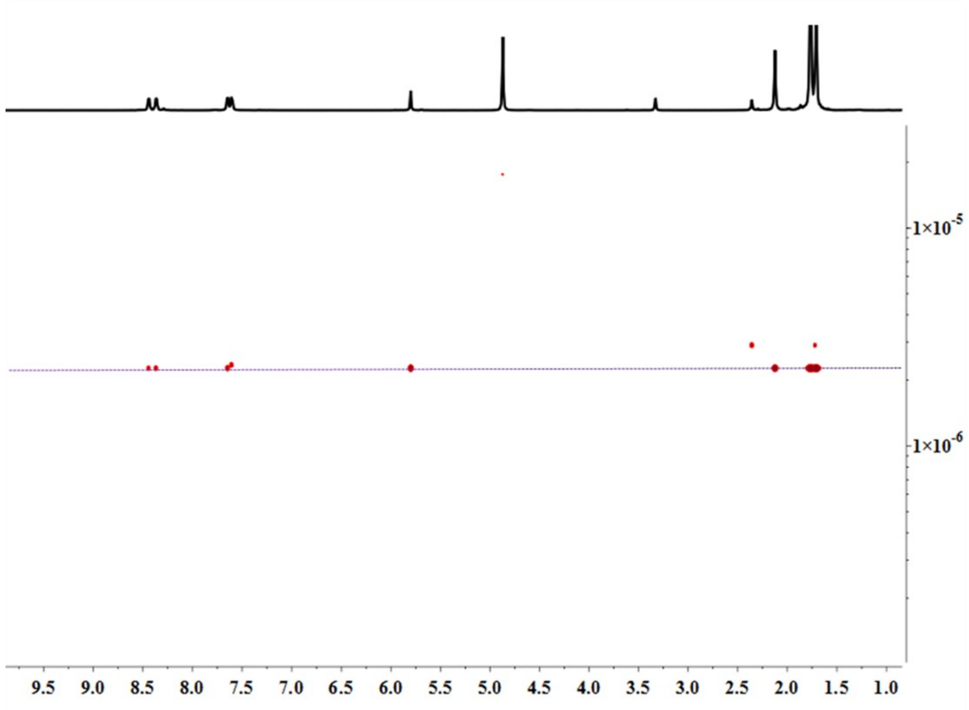

Fig. S17. ^{1}H-^{1}H DOSY NMR (500 MHz, CD_3OD, ppm) for **3b** (2.32×10^{-10} m^2s^{-1}) (15.0 mM, with respect to Cp*Rh)

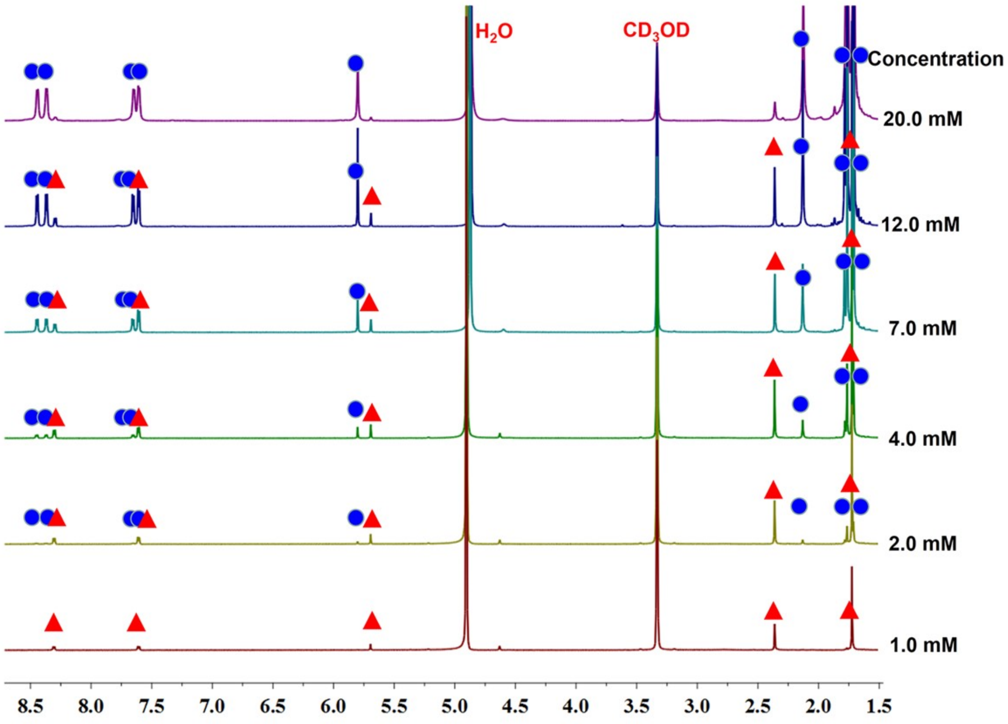

Fig. S18. The ^{1}H NMR (500 MHz, CD_3OD, ppm) for **3a** and **3b**, showing that an increase in concentration induced the transformation of tetranuclear macrocycle **3a** into the [2] catenane **3b** (1.0-20.0 mM, with respect to Cp*Rh).

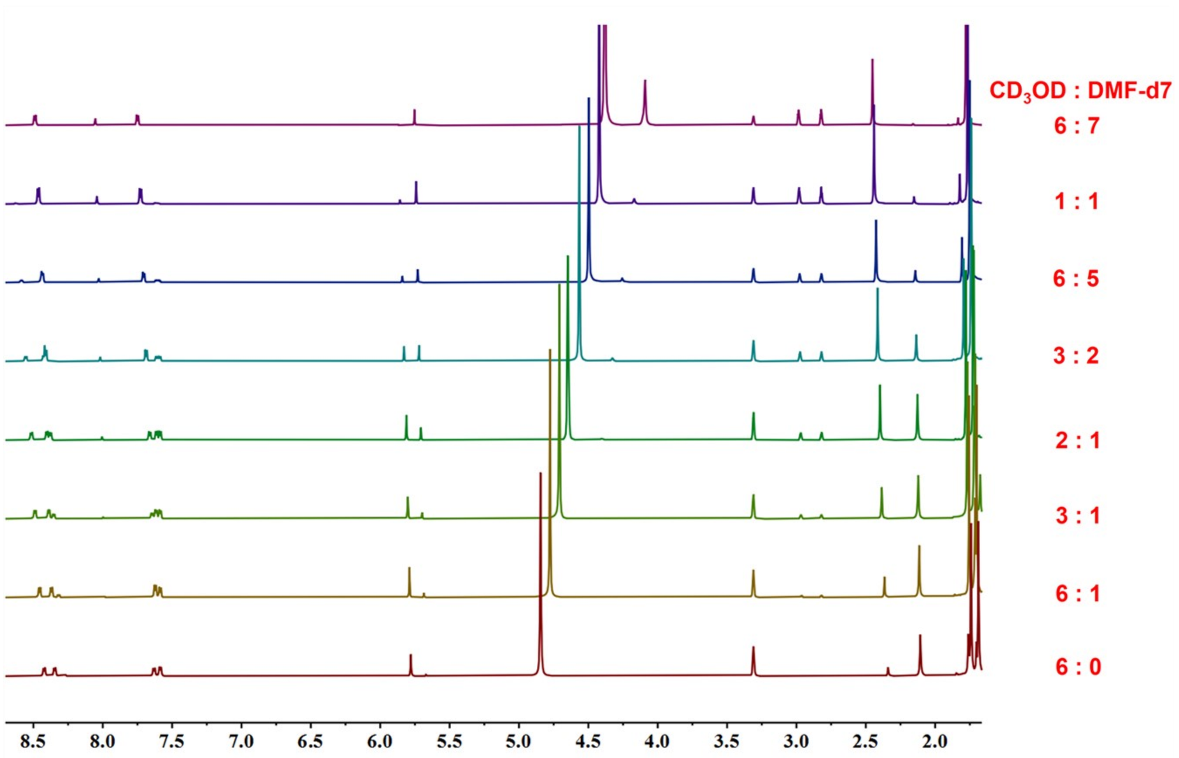

Fig. S19. The full ^{1}H NMR spectra showing the interconversion between [2] catenane **3b** and tetranuclear macrocycle **3a** upon changing solvent ratio (CD_3OD/DMF-d7 [20.0 mM, with respect to Cp*Rh], 500 MHz).

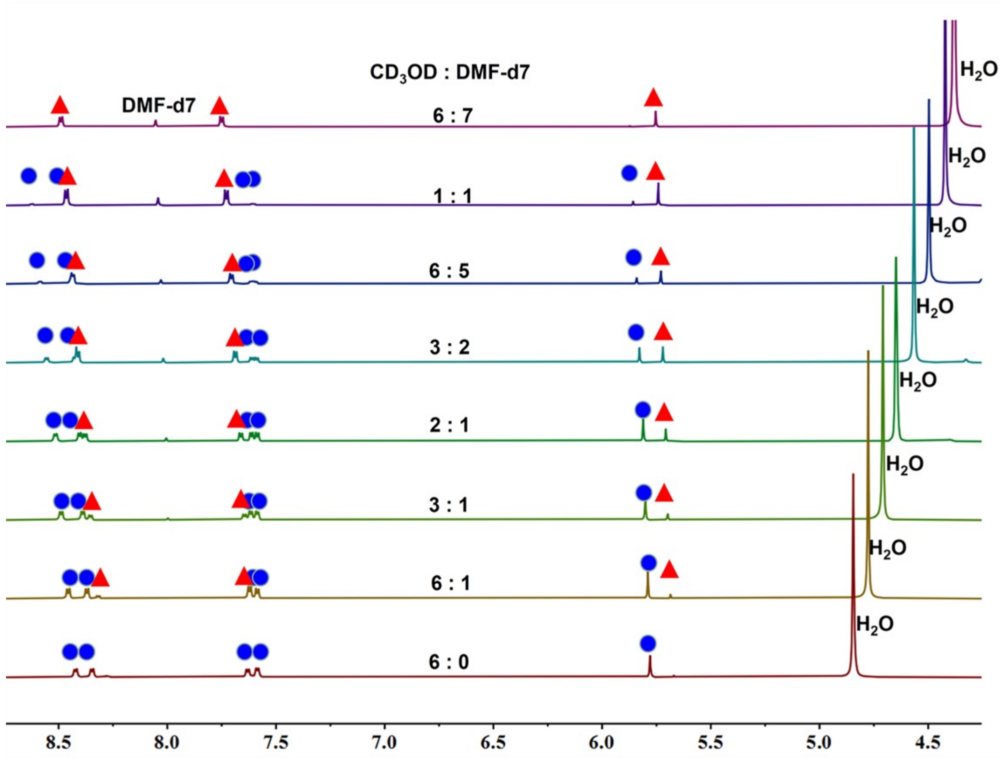

Fig. S20. The partial ^{1}H NMR spectra showing the interconversion between [2] catenane **3b** and tetranuclear macrocycle **3a** upon changing solvent ratio (CD_3OD/DMF-d7 [20.0 mM, with respect to Cp*Rh], 500 MHz).

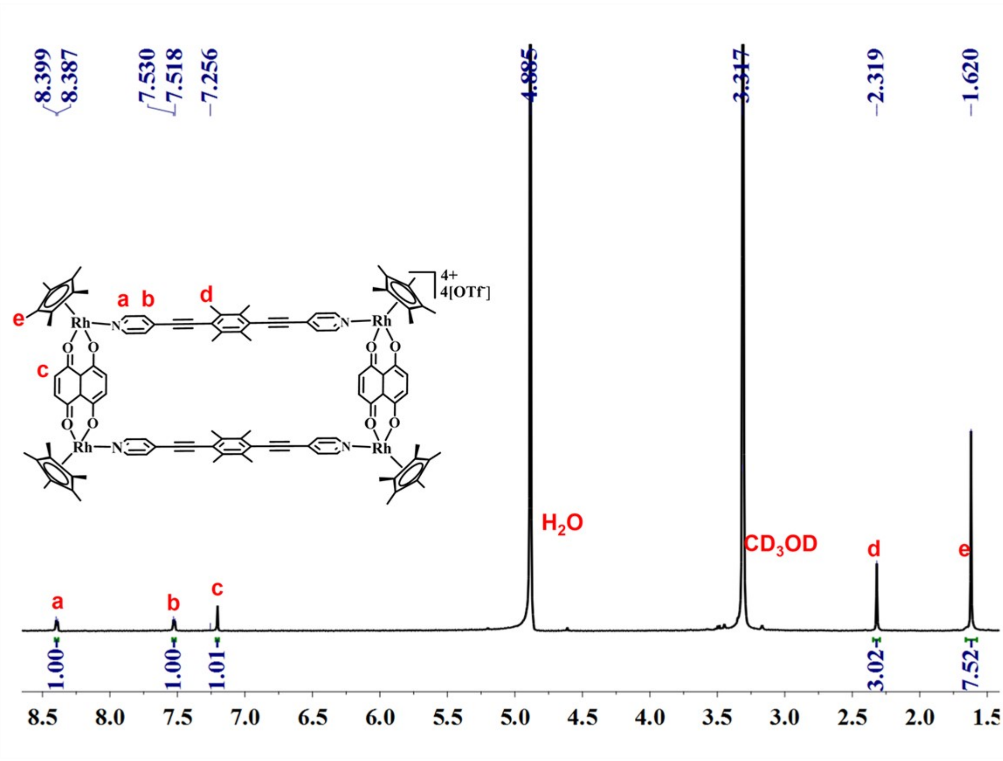

Fig. S21. The ^{1}H NMR (500 MHz, CD_3OD, ppm) for **4a** (0.8 mM, with respect to Cp*Rh)

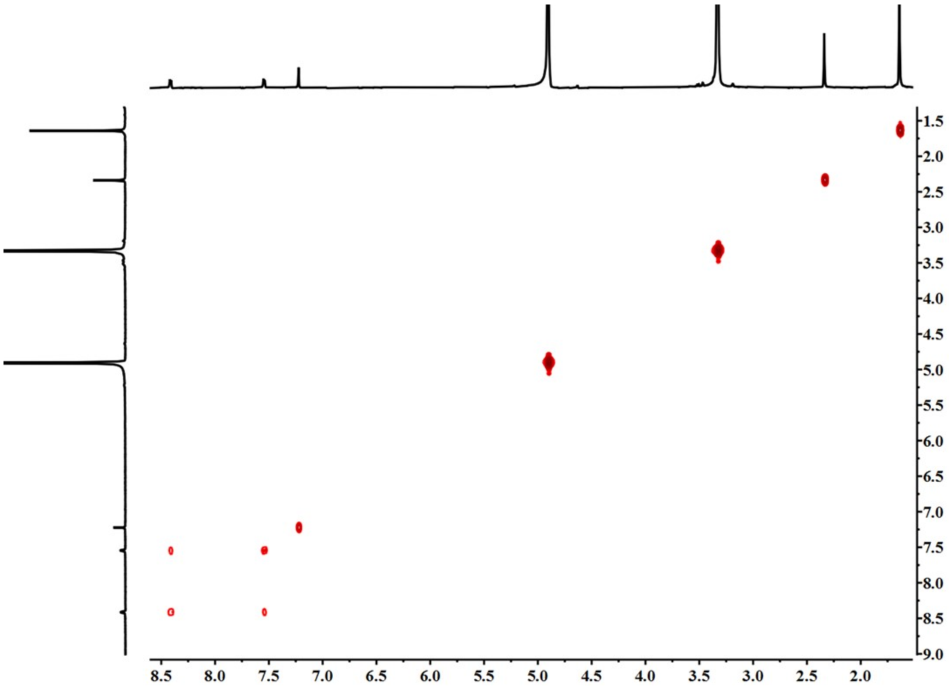

Fig. S22. The ^{1}H-^{1}H COSY NMR (500 MHz, CD_3OD, ppm) for **4a** (0.8 mM, with respect to Cp*Rh)

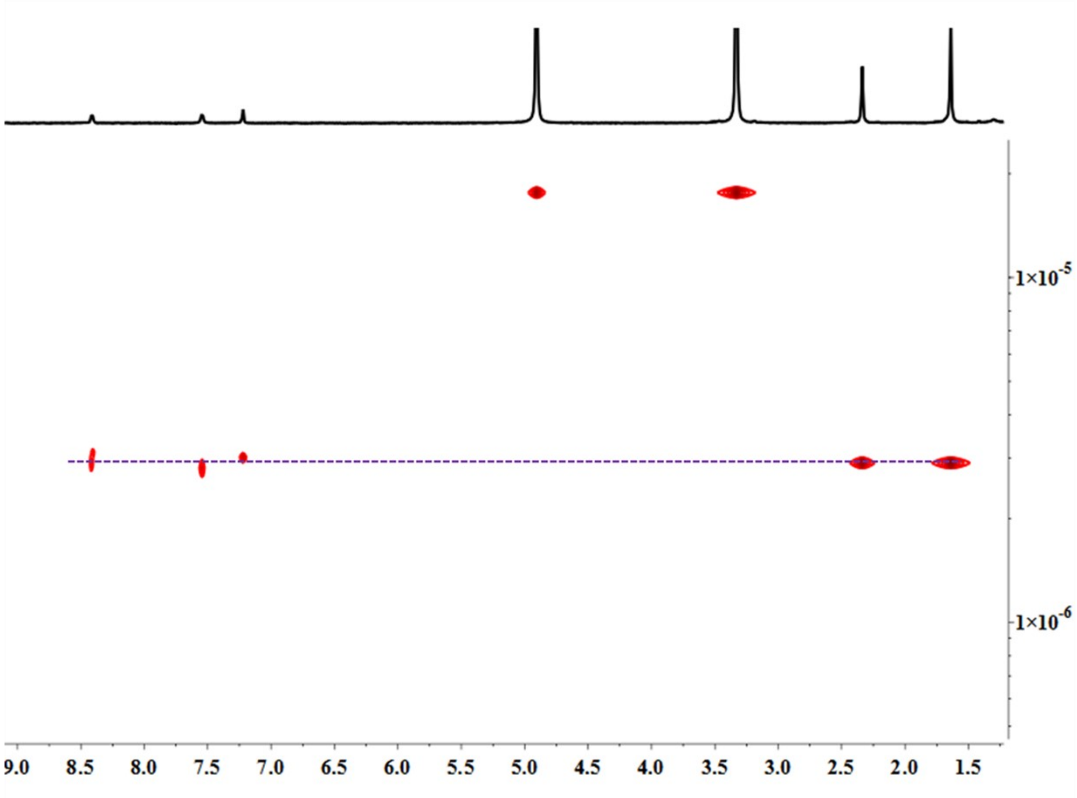

Fig. S23. The ^{1}H-^{1}H DOSY NMR (500 MHz, CD_3OD, ppm) for **4a** (2.86×10^{-10} m^2s^{-1}) (0.8 mM, with respect to Cp*Rh)

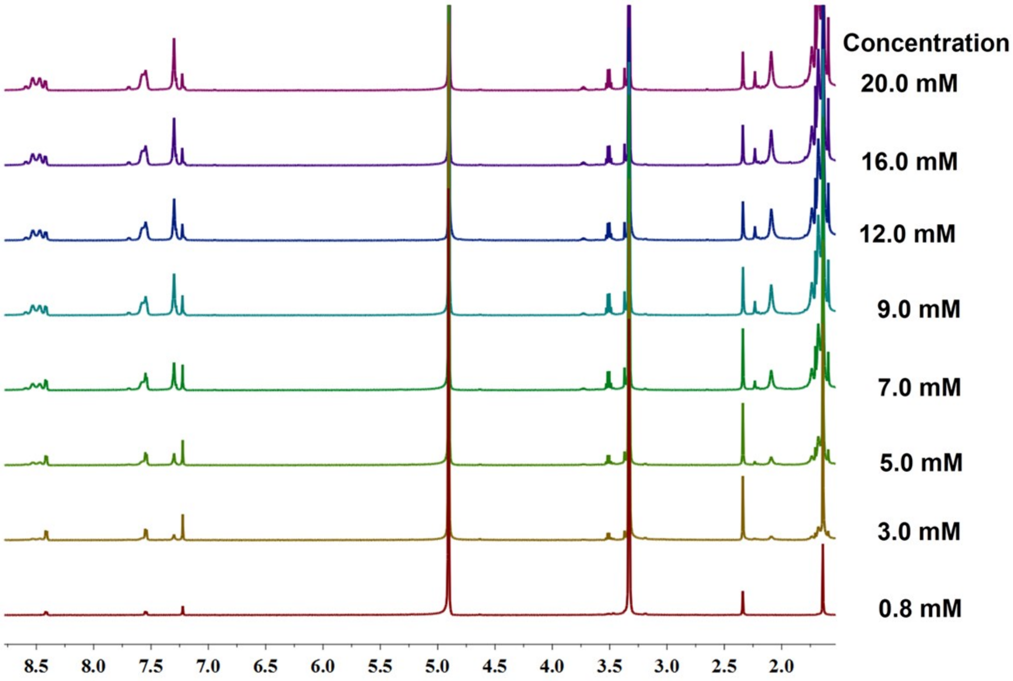

Fig. S24. The ^{1}H NMR (500 MHz, CD_3OD, ppm) for **4a** and **4b**, showing that an increase in concentration induced the transformation of tetranuclear macrocycle **4a** into the [2] catenane **4b** (0.8-20.5 mM, with respect to Cp*Rh).

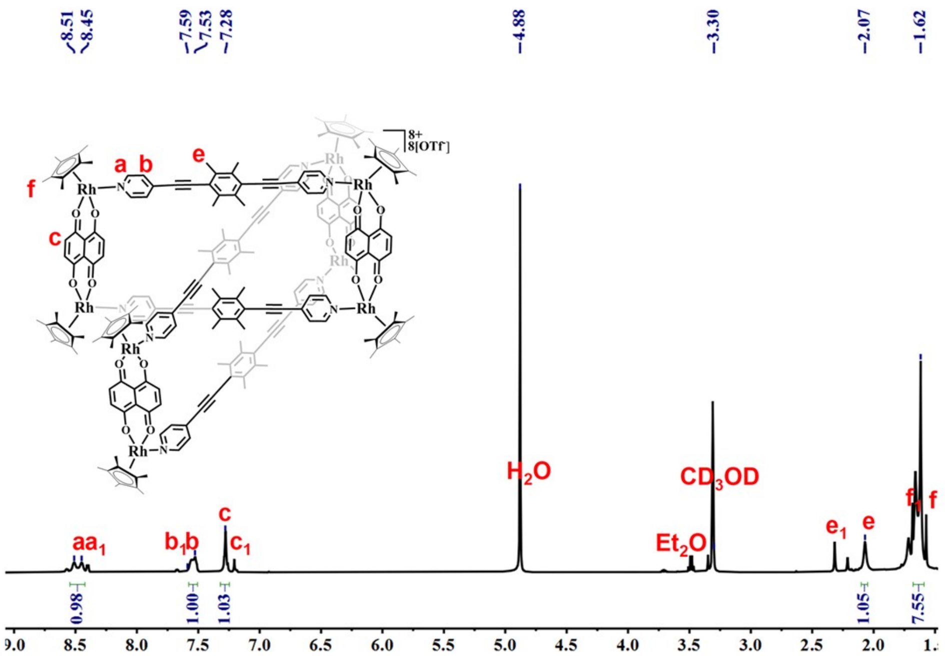

Fig. S25. The ^{1}H NMR (500 MHz, CD_3OD, ppm) for **4b**, these weak small peaks (a_1, b_1, c_1, e_1, f_1) are attributed to the single ring signals (20.5 mM, with respect to Cp*Rh)

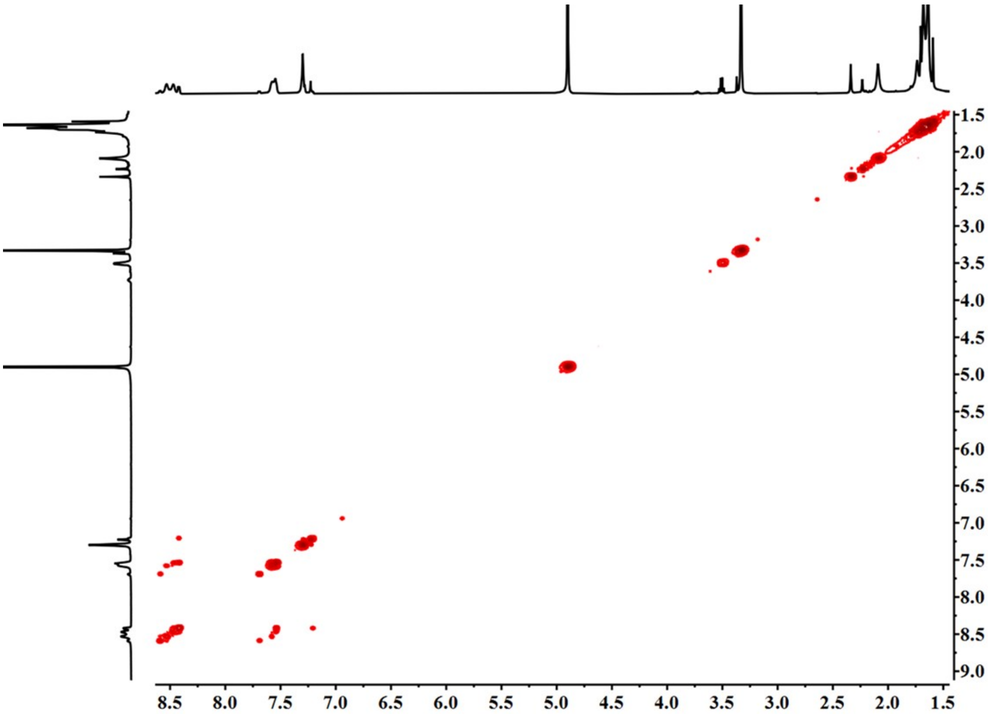

Fig. S26. The ^{1}H-^{1}H COSY NMR (500 MHz, CD_3OD, ppm) for **4b** (0.5 mM, with respect to Cp*Rh)

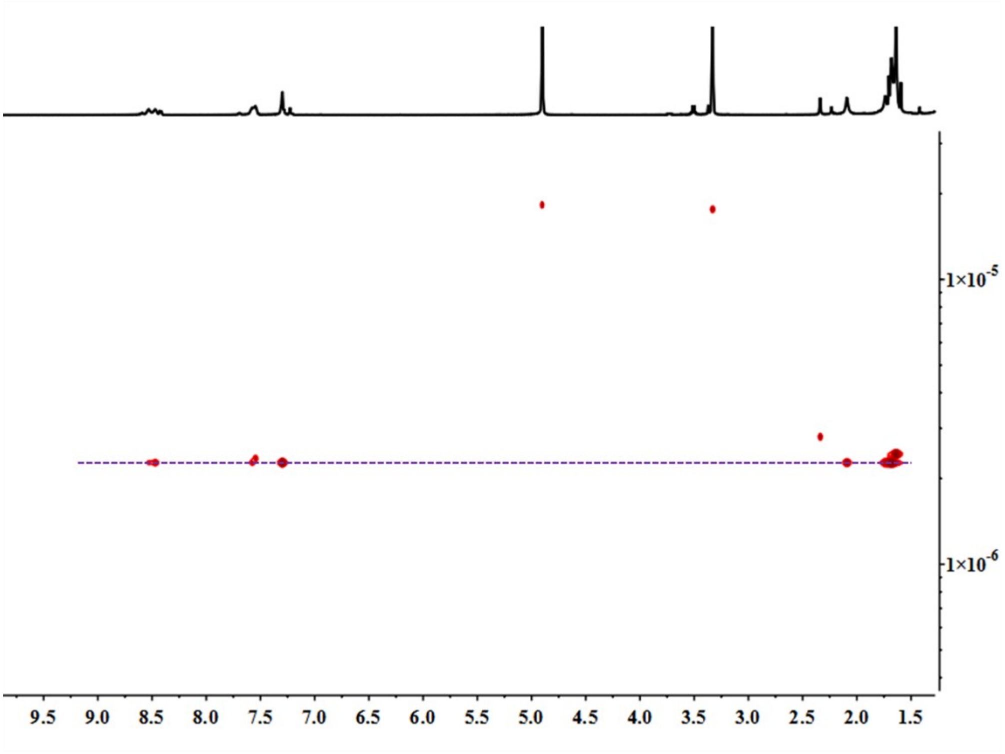

Fig. S27. The ^{1}H-^{1}H DOSY NMR (500 MHz, CD_3OD, ppm) for **4b** (2.22×10^{-10} m^2s^{-1}) (20.5 mM, with respect to Cp*Rh)

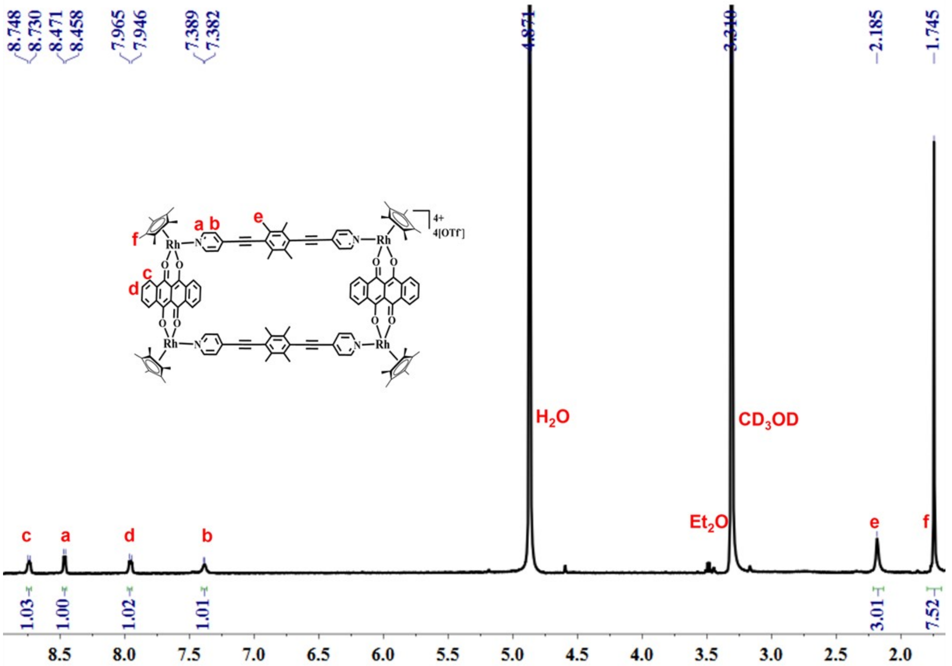

Fig. S28. The ^{1}H NMR (500 MHz, CD_3OD, ppm) for **5a** (0.5 mM, with respect to Cp*Rh)

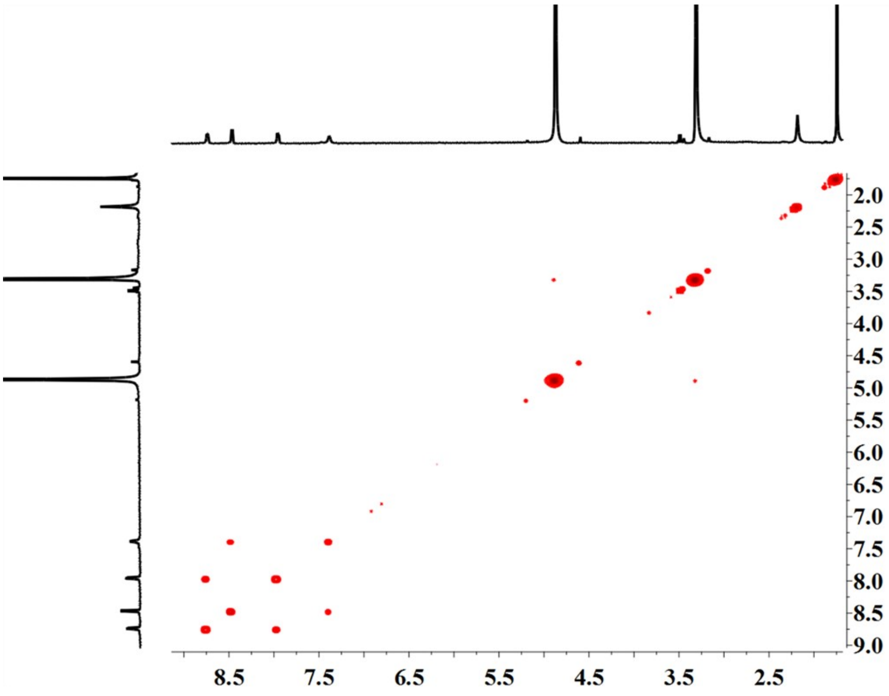

Fig. S29. The ^1H-^1H COSY NMR (500 MHz, CD_3OD, ppm) for **5a** (0.5 mM, with respect to Cp*Rh)

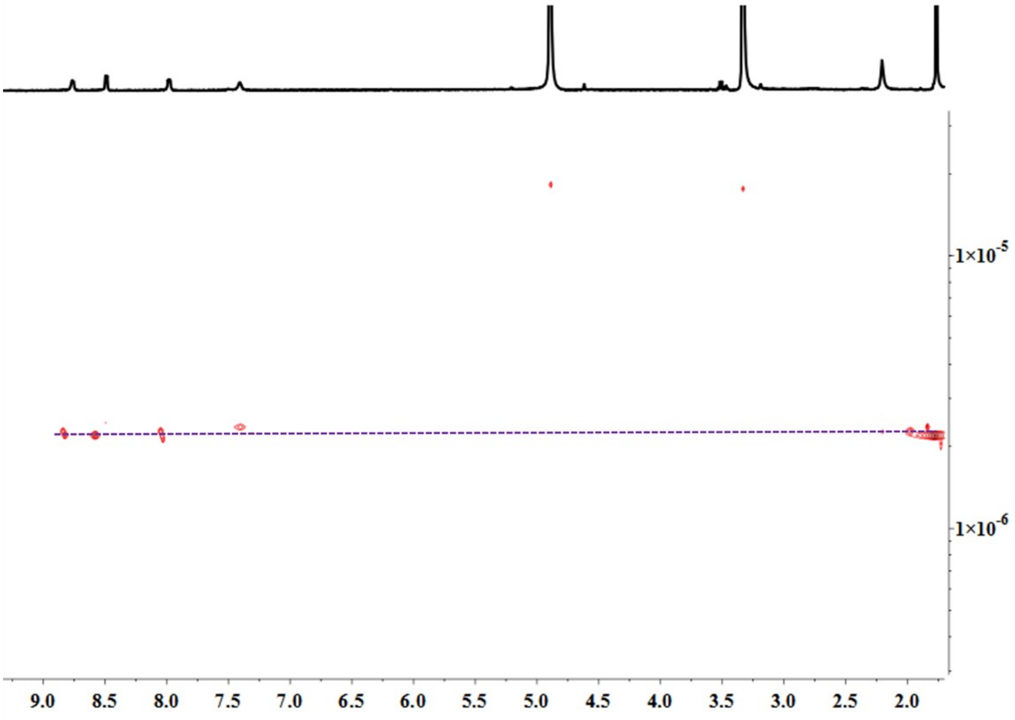

Fig. S30. The ^1H-^1H DOSY NMR (500 MHz, CD_3OD, ppm) for **5a** (2.24×10^{-10} m^2s^{-1}) (0.5 mM, with respect to Cp*Rh)

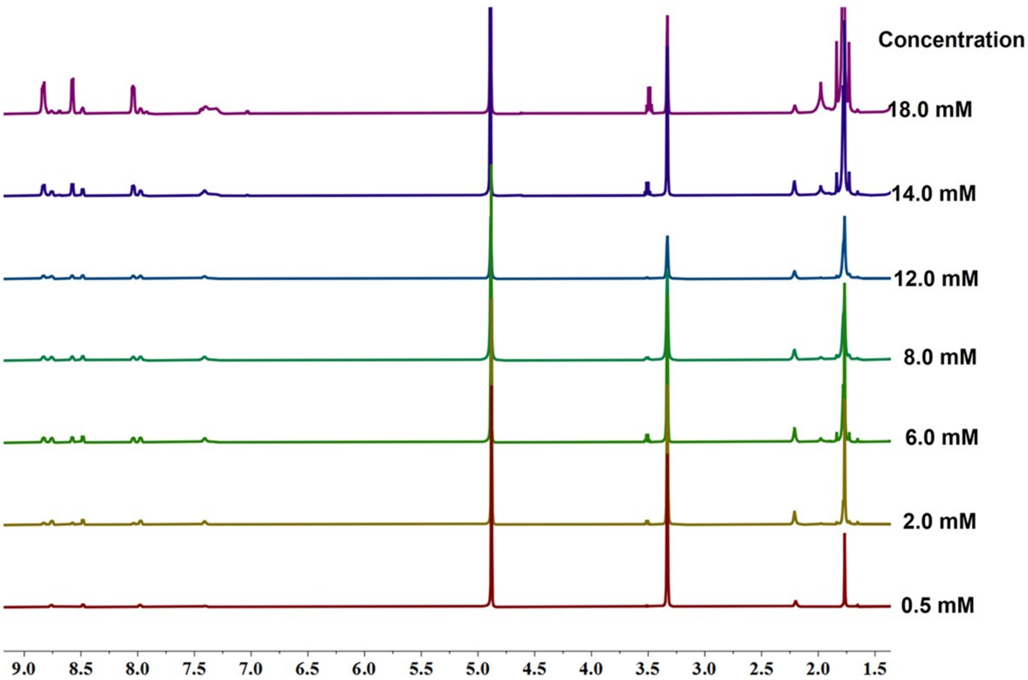

Fig. S31. The ^{1}H NMR (500 MHz, CD_3OD, ppm) for **5a** and **5b**, showing that an increase in concentration induced the transformation of tetranuclear macrocycle **5a** into the [2] catenane **5b** (0.5-18.0 mM, with respect to Cp*Rh).

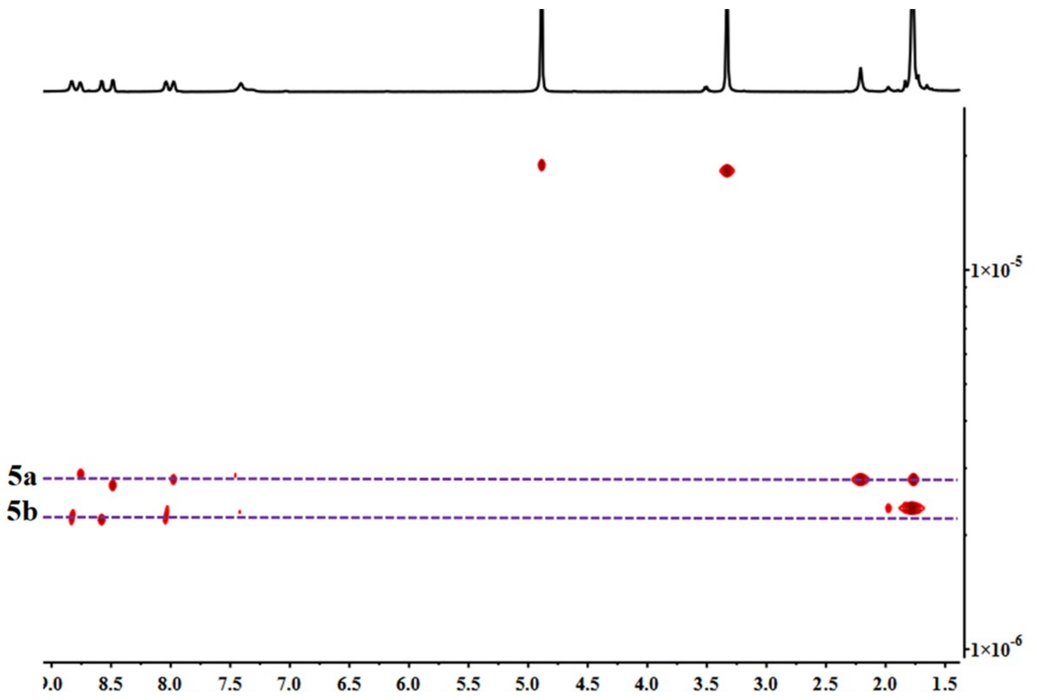

Fig. S32. The ^{1}H-^{1}H DOSY NMR (500 MHz, CD_3OD, ppm) for **5a** (2.92×10^{-10} m^2 s^{-1}) and **5b** (2.44×10^{-10} m^2s^{-1}) (7.0 mM, with respect to Cp*Rh)

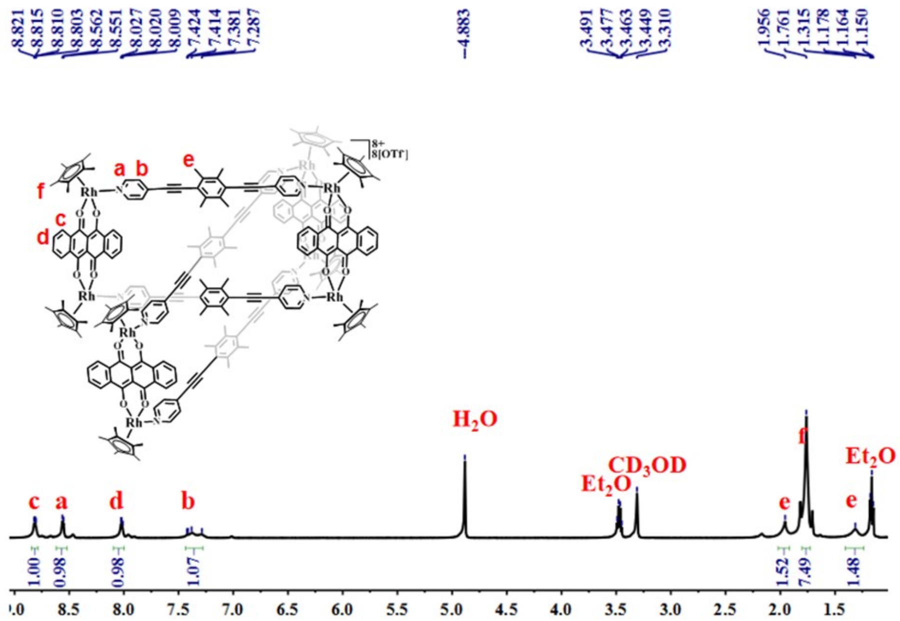

Fig. S33. The ^{1}H NMR (500 MHz, CD_3OD, ppm) for **5b** (18.0 mM, with respect to Cp*Rh)

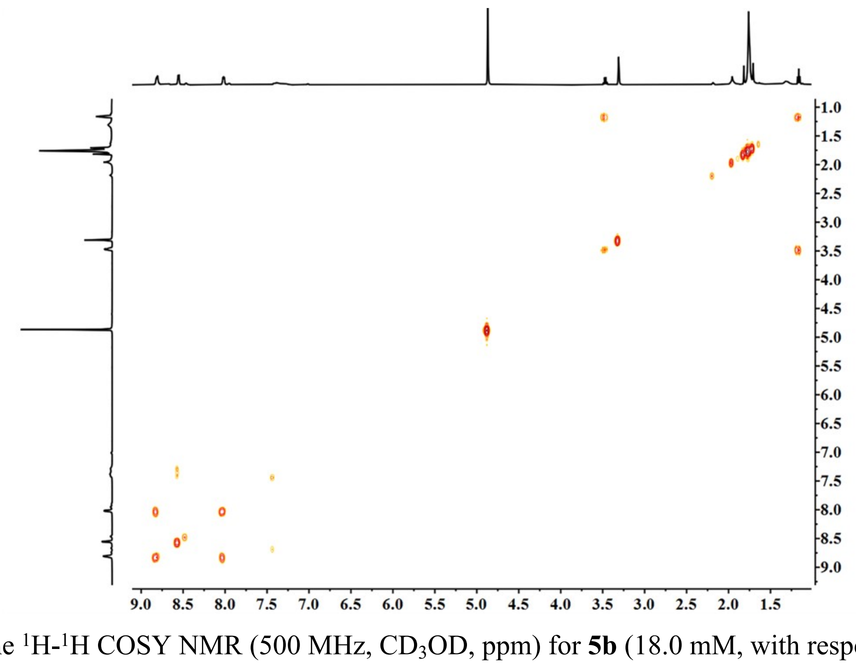

Fig. S34. The ^{1}H-^{1}H COSY NMR (500 MHz, CD_3OD, ppm) for **5b** (18.0 mM, with respect to Cp*Rh)

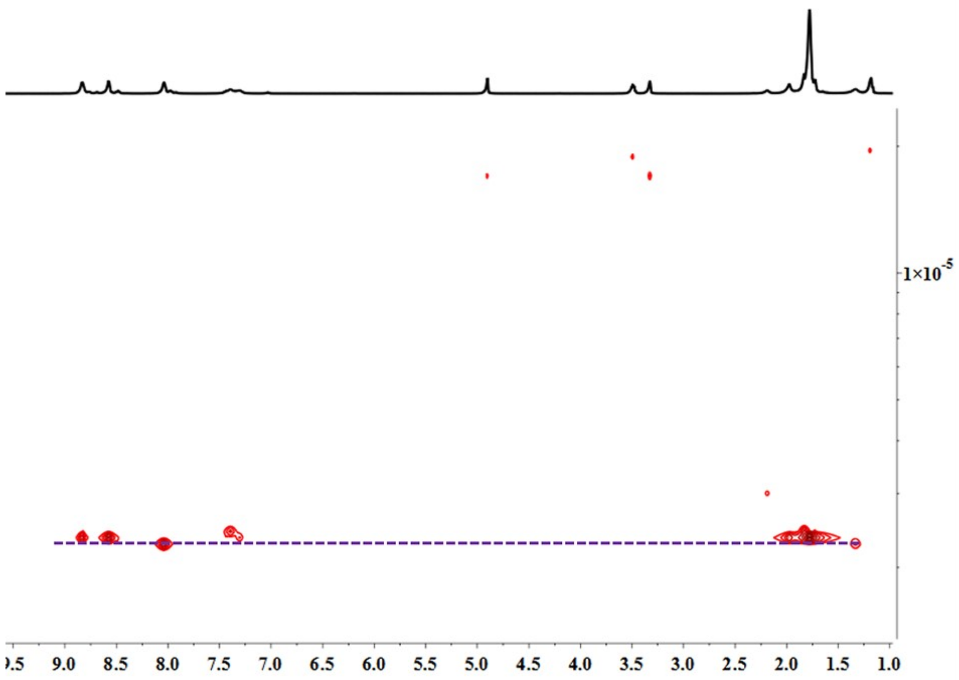

Fig. S35. The ^{1}H-^{1}H DOSY NMR (500 MHz, CD$_3$OD, ppm) for **5b** (2.44×10^{-10} m^{2}s^{-1}) (18.0 mM, with respect to Cp*Rh)

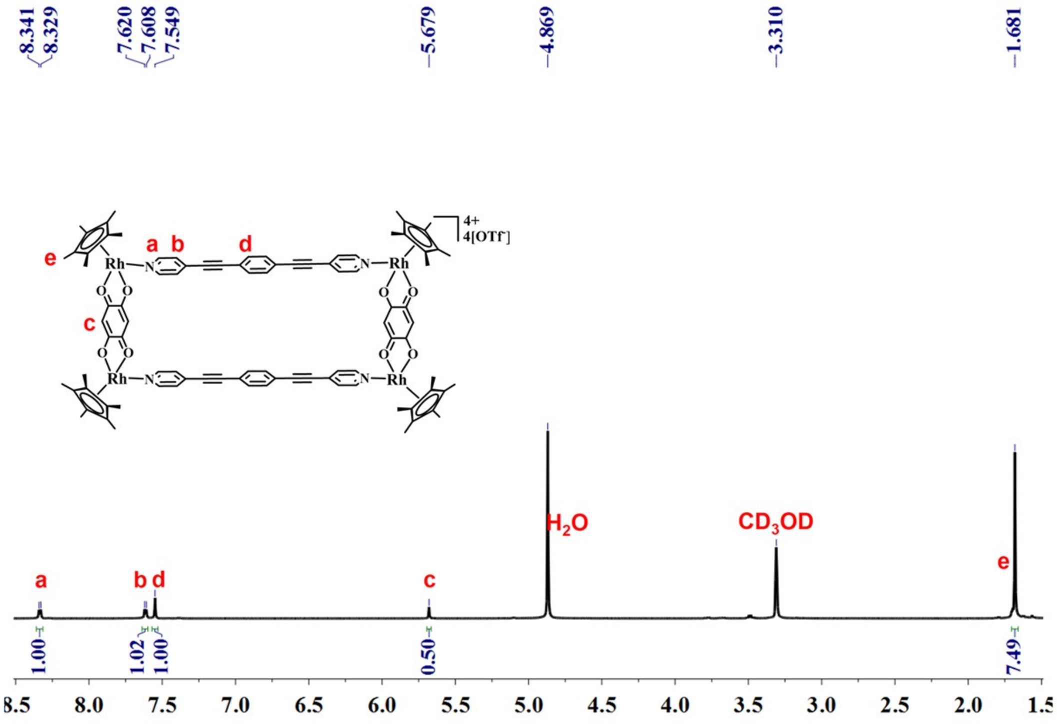

Fig. S36. The ^{1}H NMR (500 MHz, CD$_3$OD, ppm) for **6** (14.0 mM, with respect to Cp*Rh)

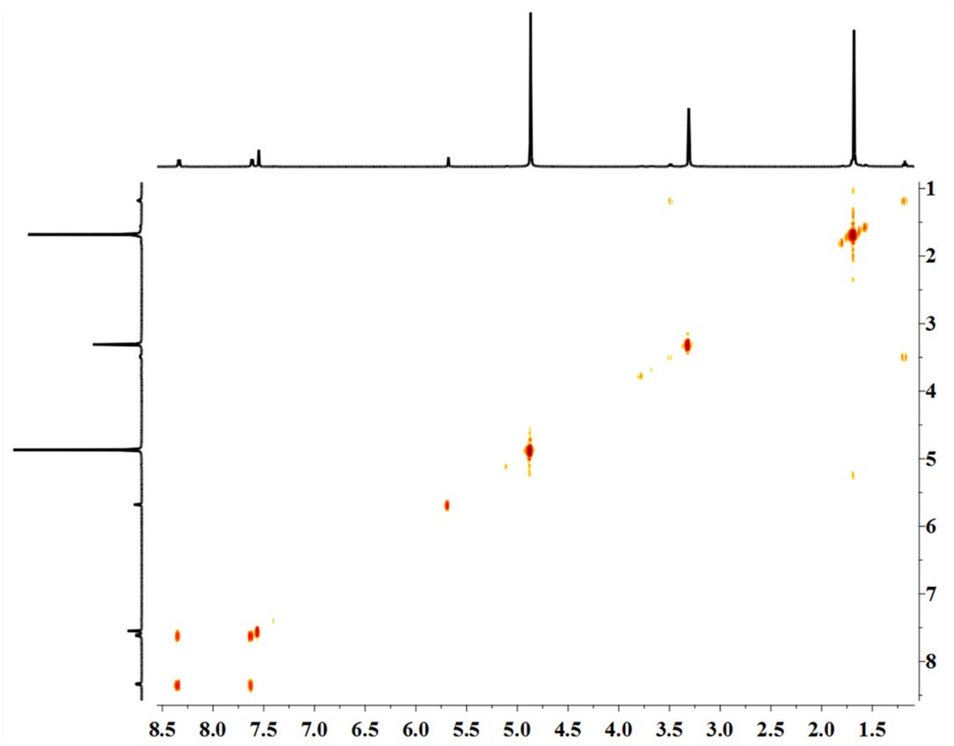

Fig. S37. The ^{1}H-^{1}H COSY NMR (500 MHz, CD_3OD, ppm) for **6** (14 mM, with respect to Cp*Rh)

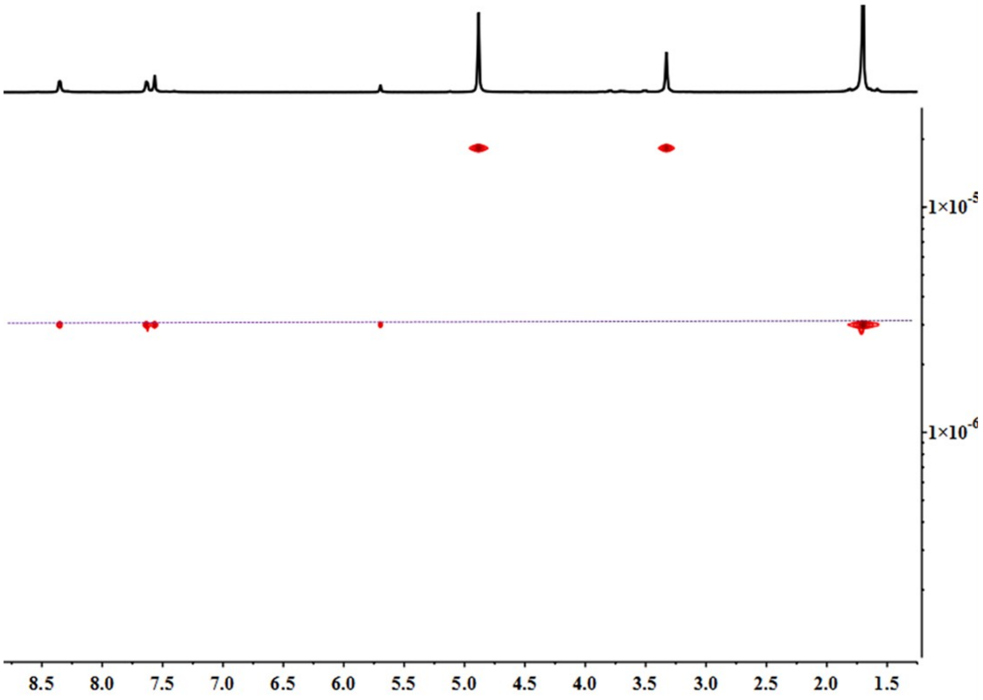

Fig. S38. The ^{1}H-^{1}H DOSY NMR (500 MHz, CD_3OD, ppm) for **6** (3.03×10^{-10} m^2s^{-1}) (14.0 mM, with respect to Cp*Rh)

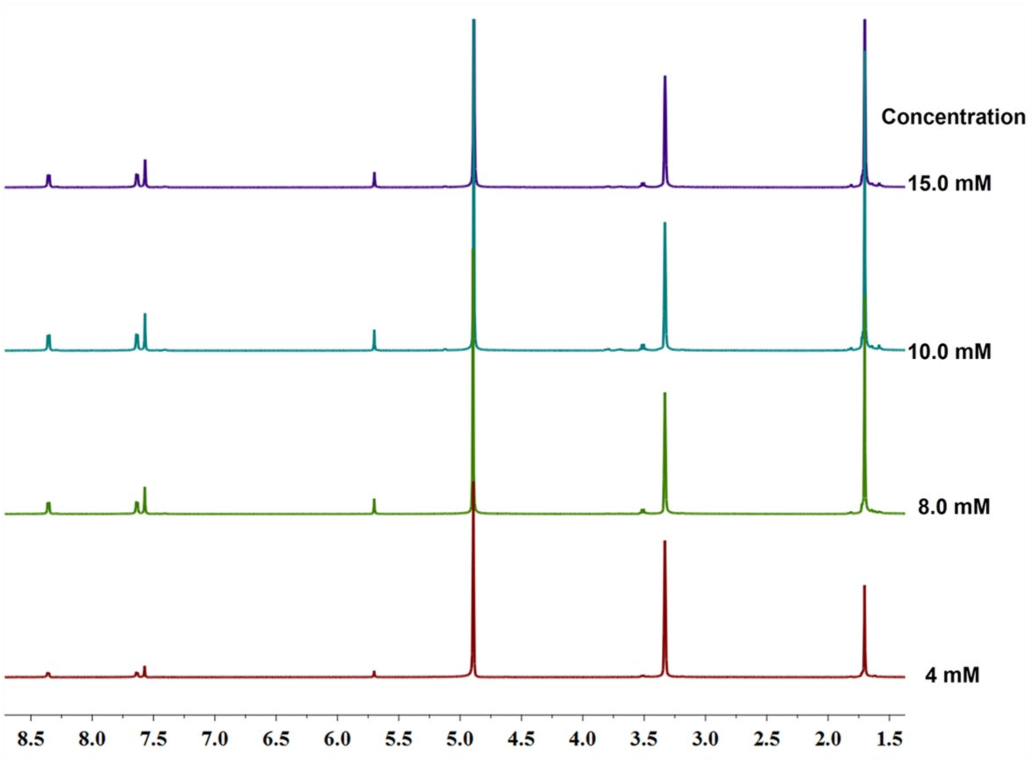

Fig. S39. The ^{1}H NMR (500 MHz, CD_3OD, ppm) for **6**, showing no appearance of new signals and disappearance of initial signals of **6** with an increase of concentration in CD_3OD. (4.0-15.0 mM, with respect to Cp*Rh).

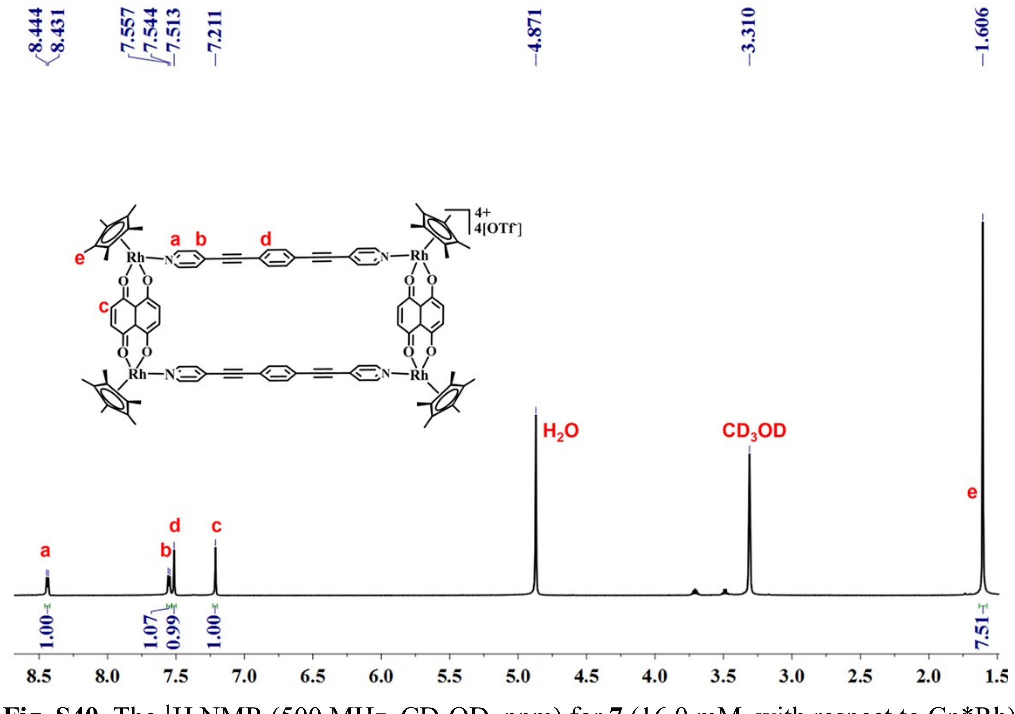

Fig. S40. The ^{1}H NMR (500 MHz, CD_3OD, ppm) for **7** (16.0 mM, with respect to Cp*Rh)

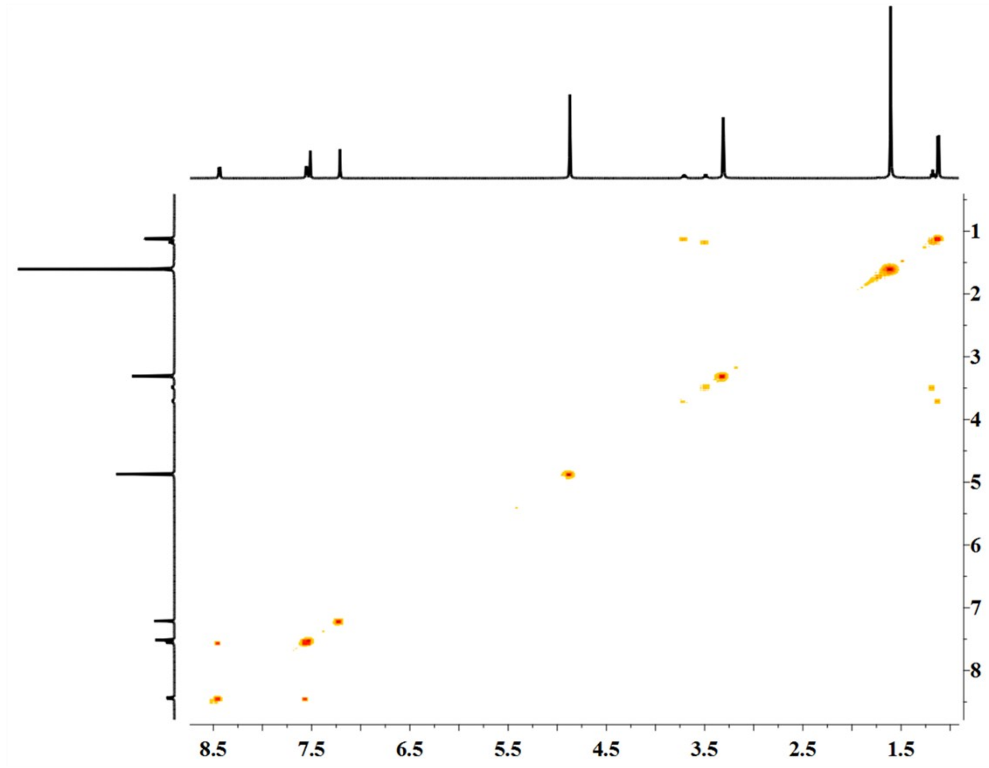

Fig. S41. The ^{1}H-^{1}H COSY NMR (500 MHz, CD_3OD, ppm) for **7** (16 mM, with respect to Cp*Rh)

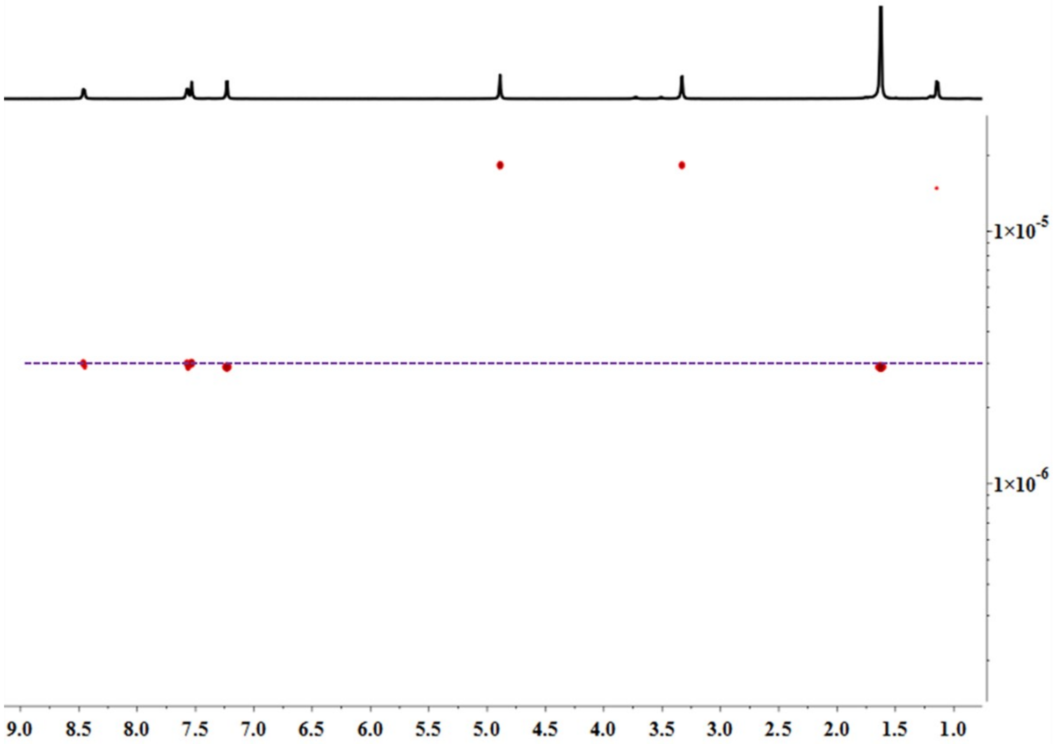

Fig. S42. The ^{1}H-^{1}H DOSY NMR (500 MHz, CD_3OD, ppm) for **7** (3.00×10^{-10} m^2s^{-1}) (16.0 mM, with respect to Cp*Rh)

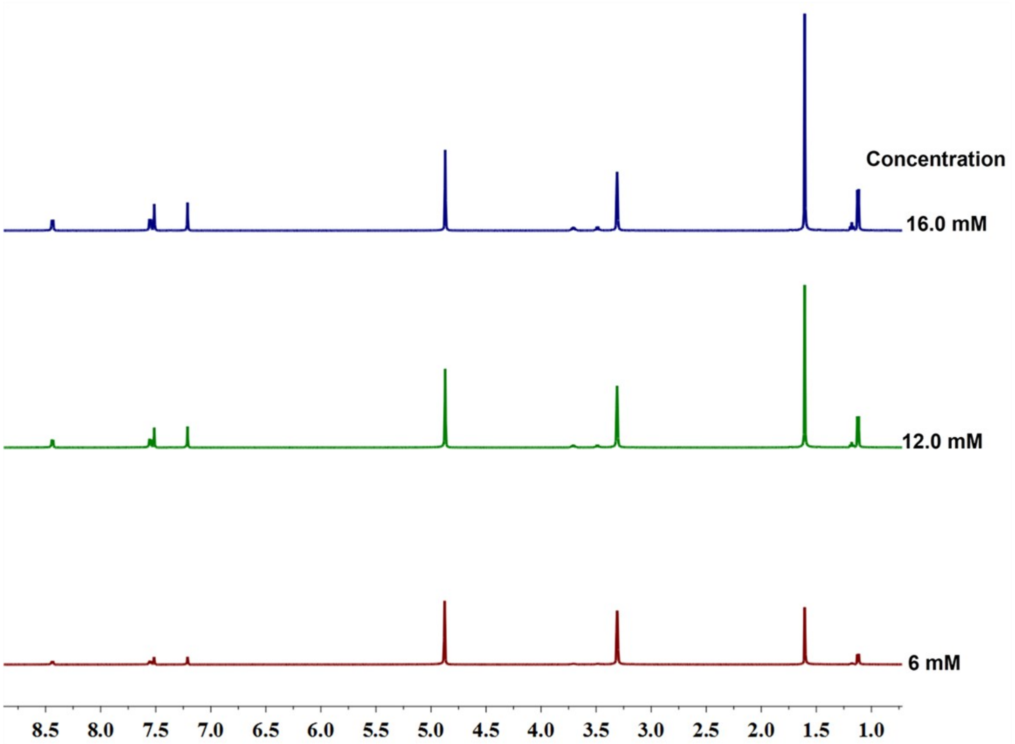

Fig. S43. The ^{1}H NMR (500 MHz, CD_3OD, ppm) for **7**, showing no appearance of new signals and disappearance of initial signals of **6** with an increase of concentration in CD_3OD. (6.0-16.0 mM, with respect to Cp*Rh).

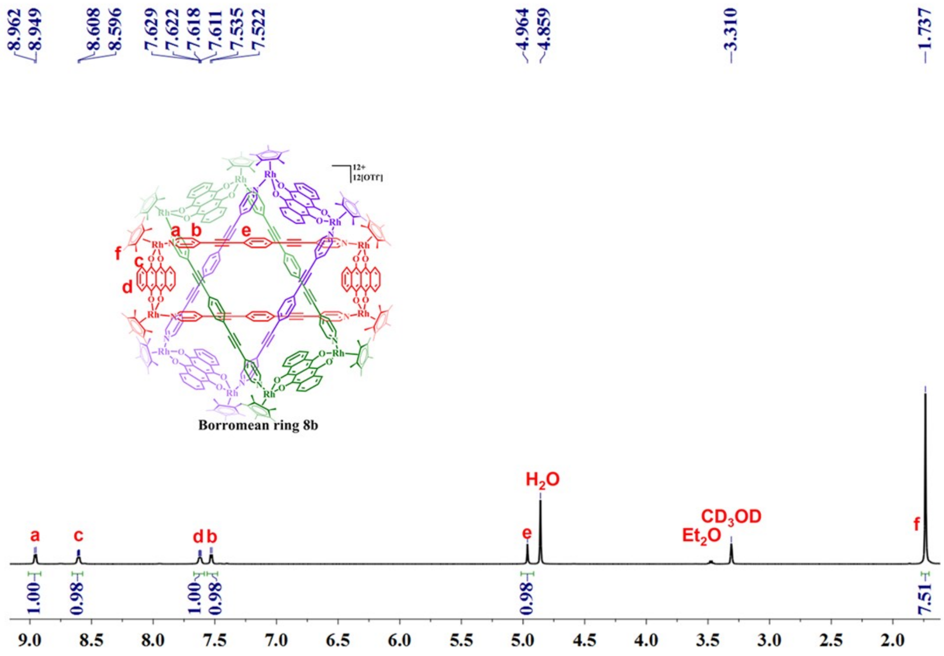

Fig. S44. The ^{1}H NMR (500 MHz, CD_3OD, ppm) for Borromean ring **8** (25.0 mM, with respect to Cp*Rh)

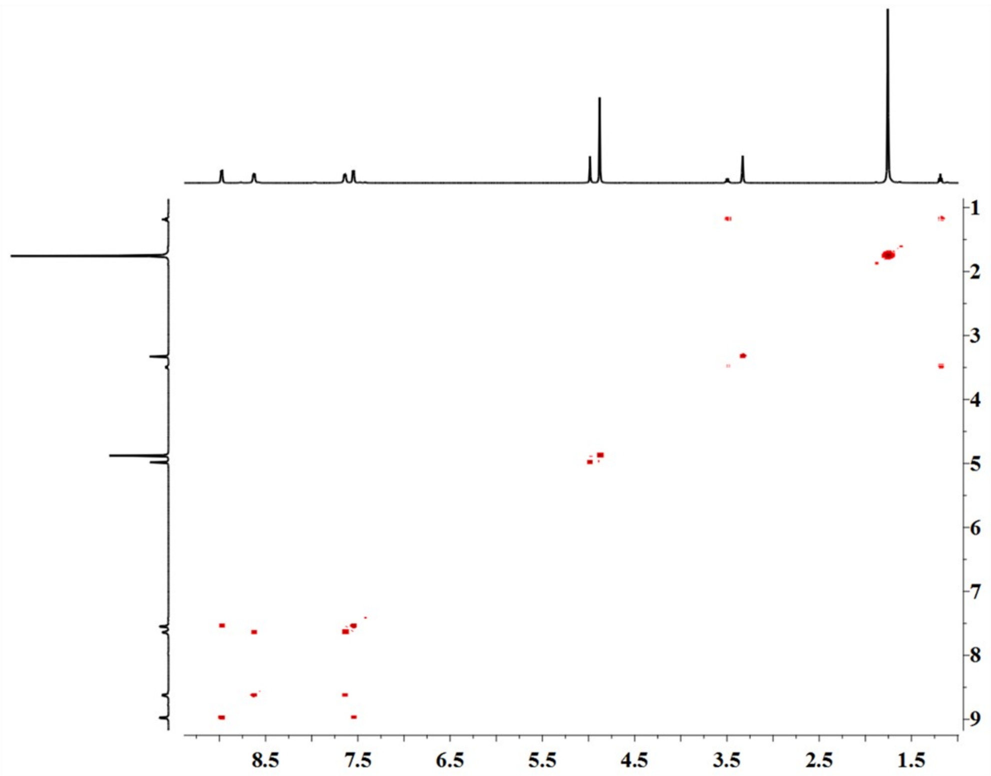

Fig. S45. The ^{1}H-^{1}H COSY NMR (500 MHz, CD_3OD, ppm) for Borromean ring **8** (25 mM, with respect to Cp*Rh)

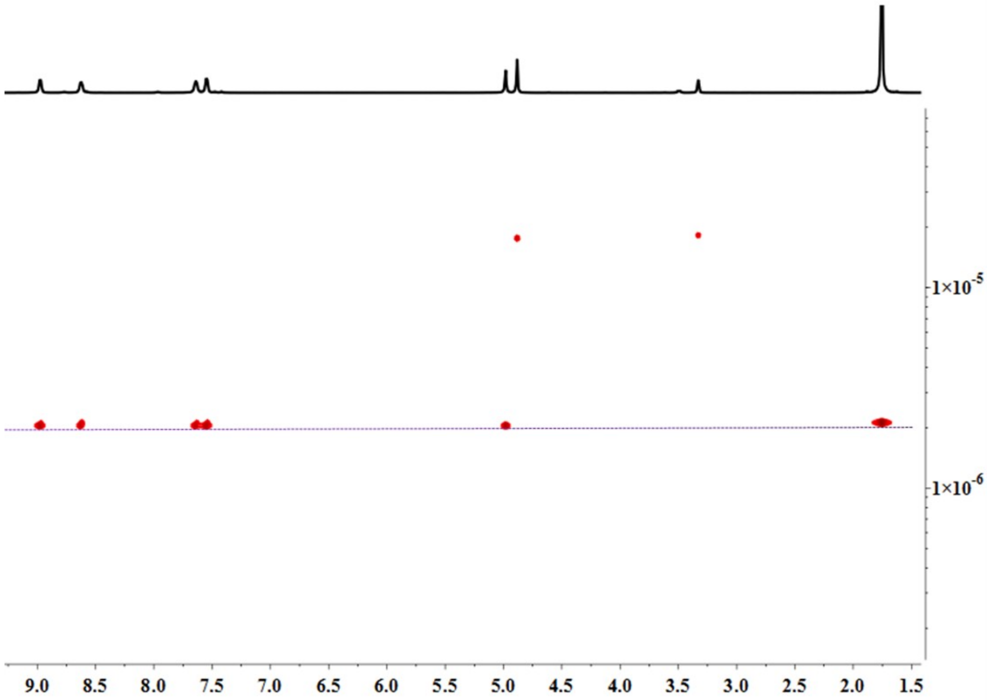

Fig. S46. The ^{1}H-^{1}H DOSY NMR (500 MHz, CD_3OD, ppm) for **8** (2.07×10^{-10} m^2s^{-1}) (16.0 mM, with respect to Cp*Rh)

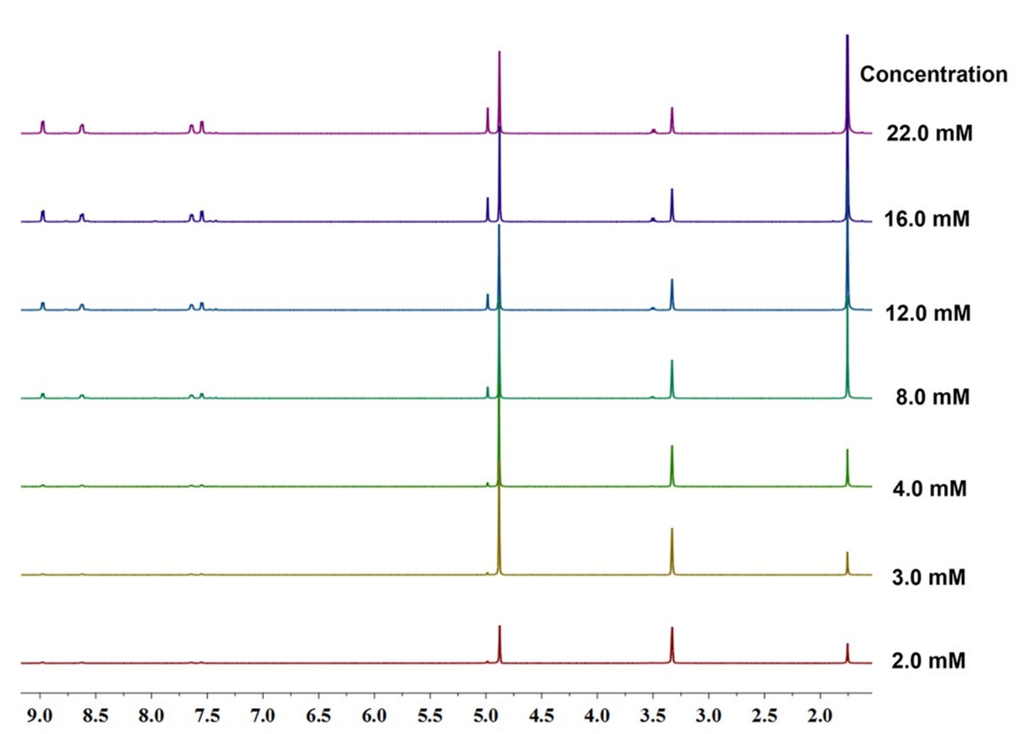

Fig. S47. The ^{1}H NMR (500 MHz, CD_3OD, ppm) for Borromean ring **8**, showing no appearance of new signals and disappearance of initial signals of **8** with an increase of concentration in CD_3OD. (2.0-22.0 mM, with respect to Cp*Rh).

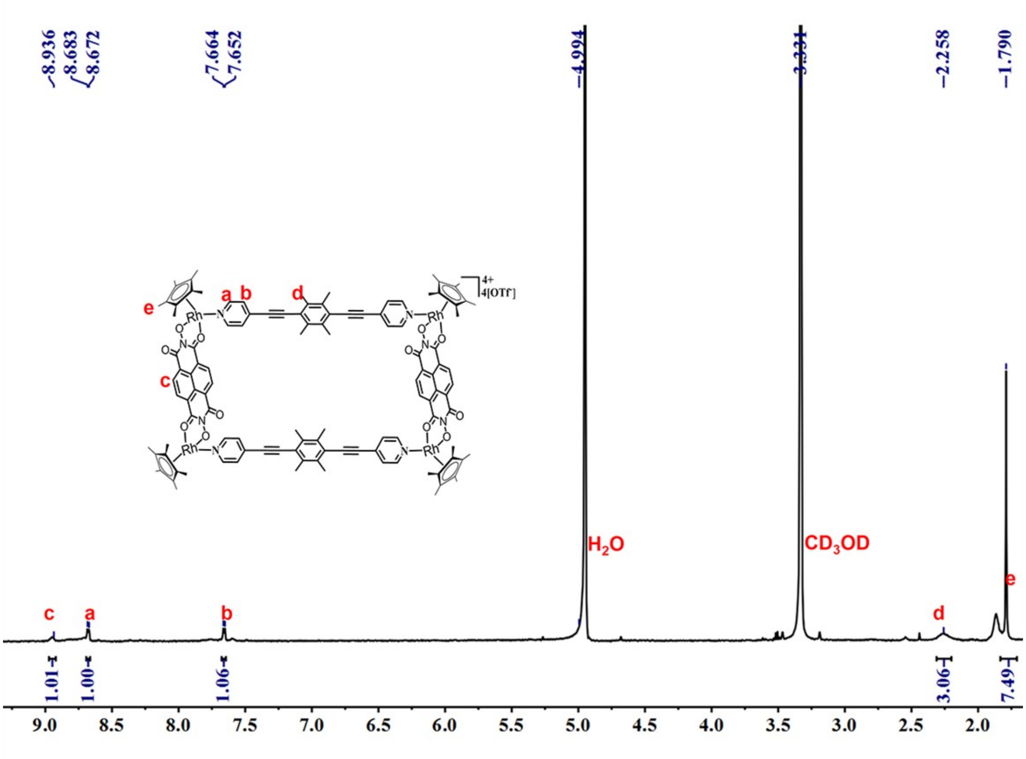

Fig. S48. The ^{1}H NMR (500 MHz, CD_3OD, ppm) for **9a** (0.5 mM, with respect to Cp*Rh)

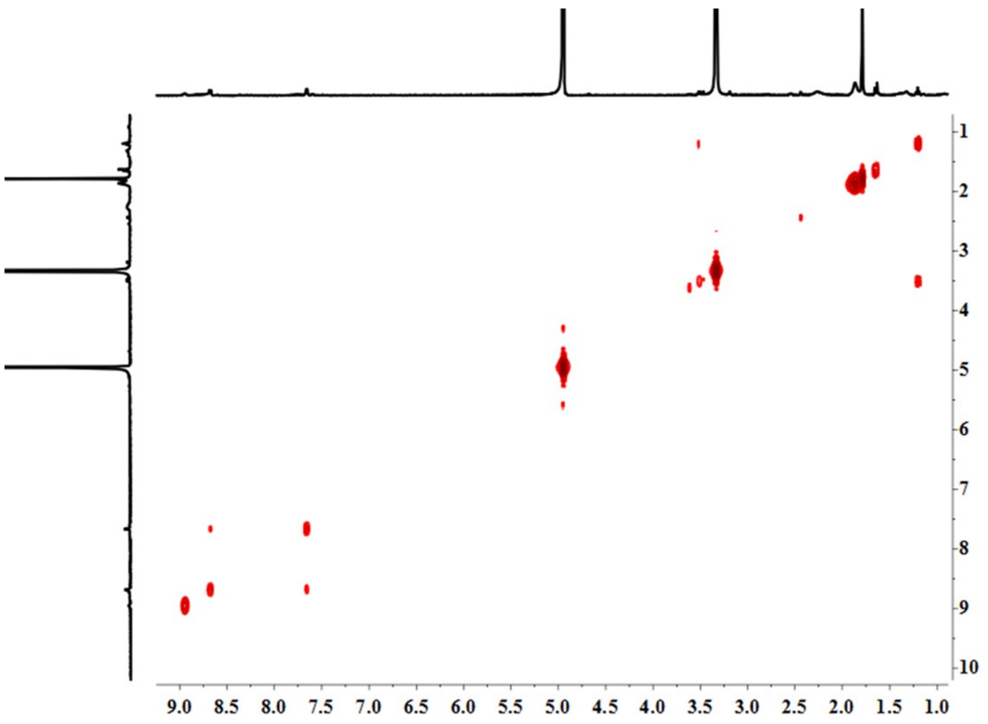

Fig. S49. The ^{1}H-^{1}H COSY NMR (500 MHz, CD_3OD, ppm) for **9a** (0.5 mM, with respect to Cp*Rh)

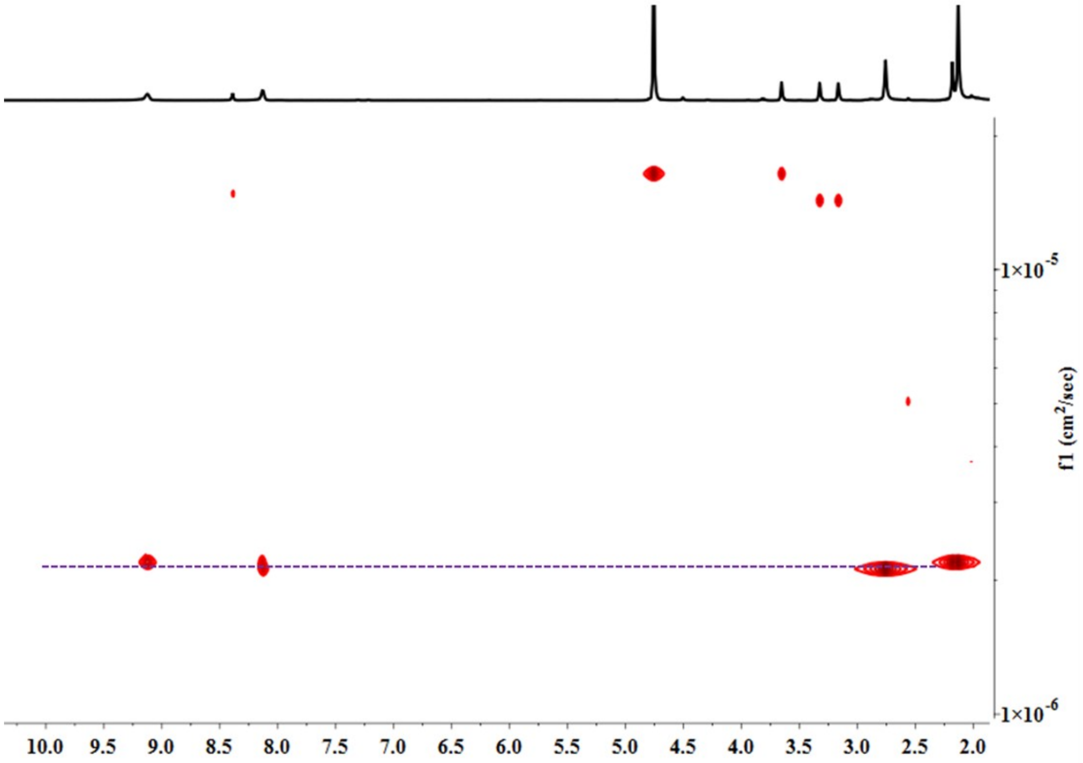

Fig. S50. The ^{1}H-^{1}H DOSY NMR (500 MHz, CD_3OD and DMF-d7, ppm) for **9a** (2.17×10^{-10} m^2s^{-1}) (16.0 mM, with respect to Cp*Rh)

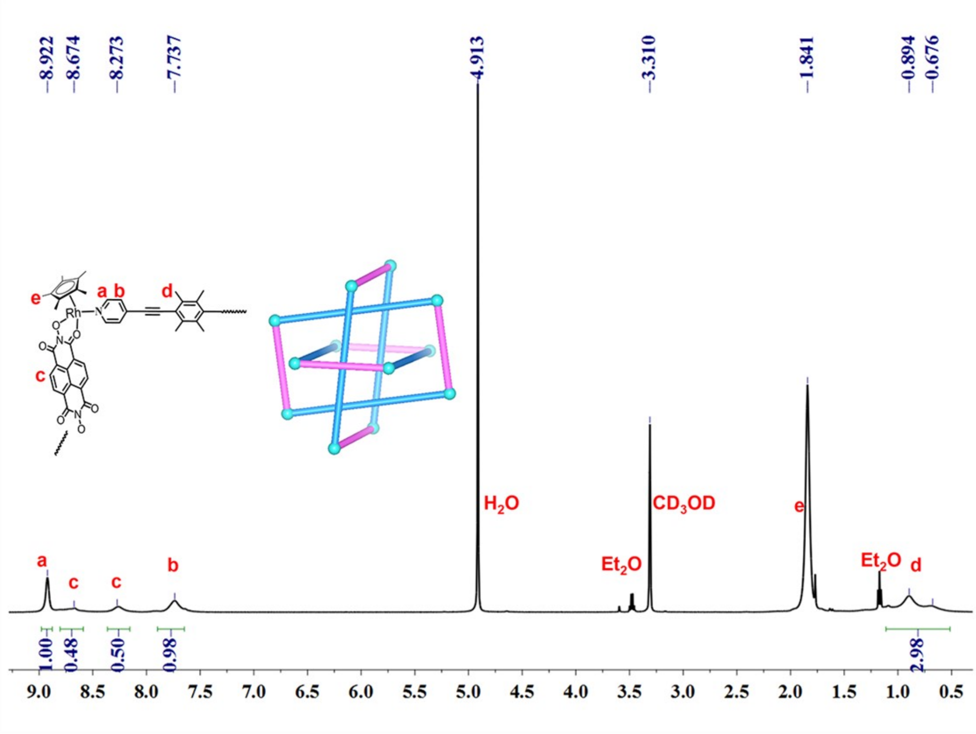

Fig. S51. The ^{1}H NMR (500 MHz, CD_3OD, ppm) for borromean ring **9b** (15.0 mM, with respect to Cp*Rh)

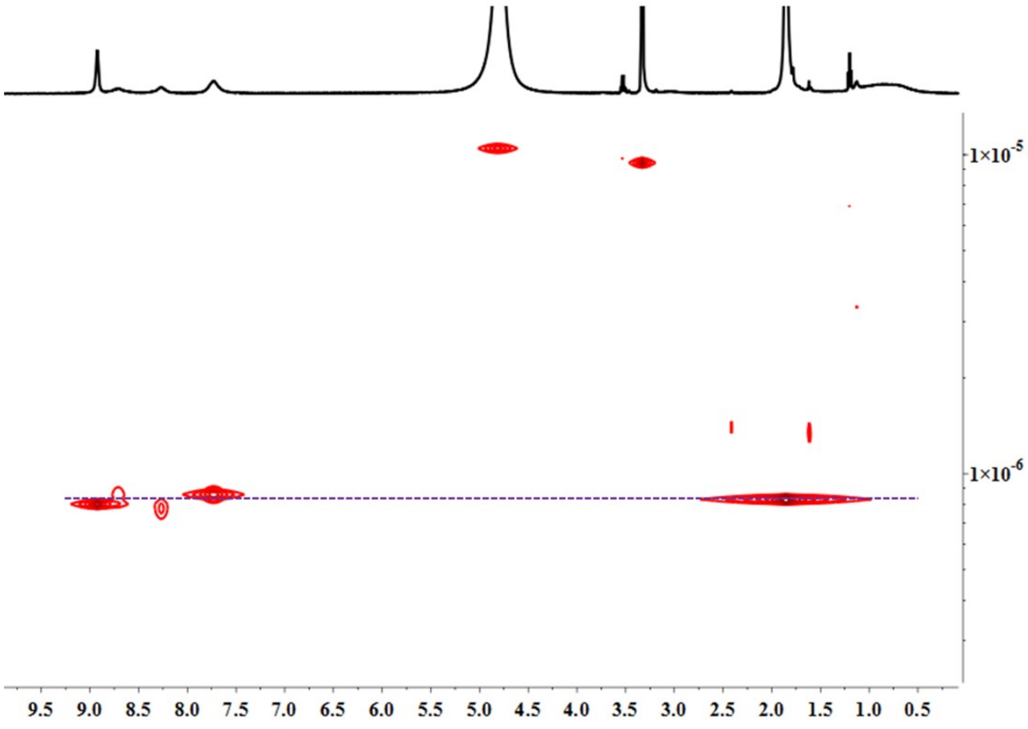

Fig. S52. The ^{1}H-^{1}H DOSY NMR (500 MHz, CD_3OD, ppm) for **9b** (7.94×10^{-11} m^2s^{-1}) (16.0 mM, with respect to Cp*Rh)

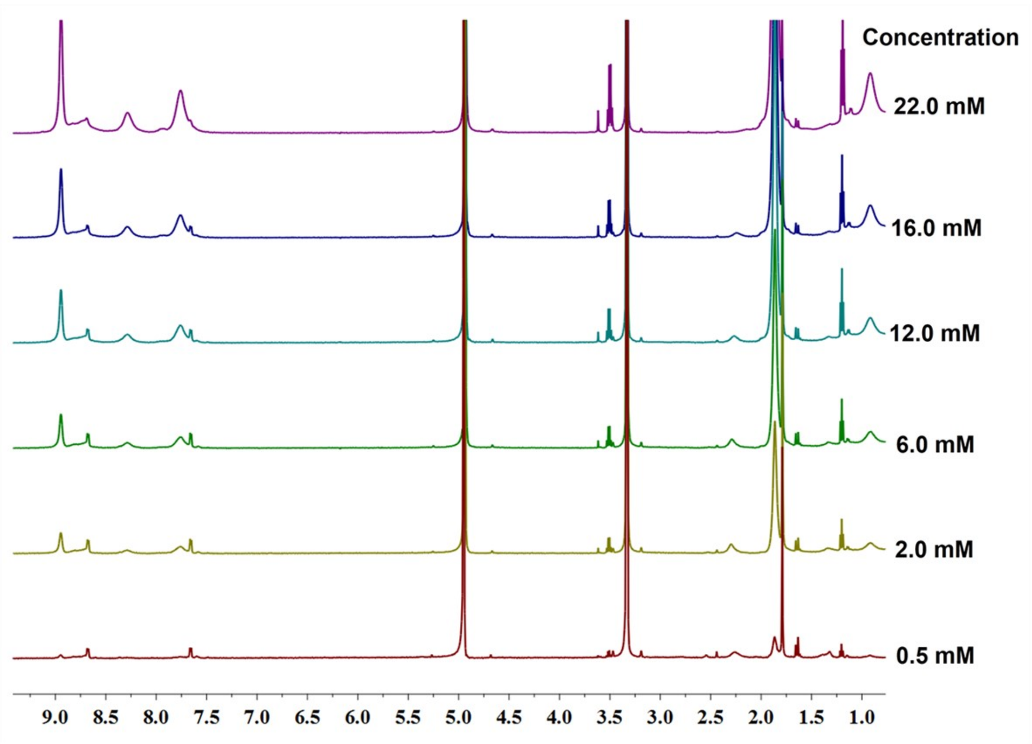

Fig. S53. The ^{1}H NMR (500 MHz, CD_3OD, ppm) for **9a** and **9b**, showing that an increase in concentration induced the transformation of tetranuclear macrocycle **9a** into the borromean ring **9b** (0.5-22.0 mM, with respect to Cp*Rh).

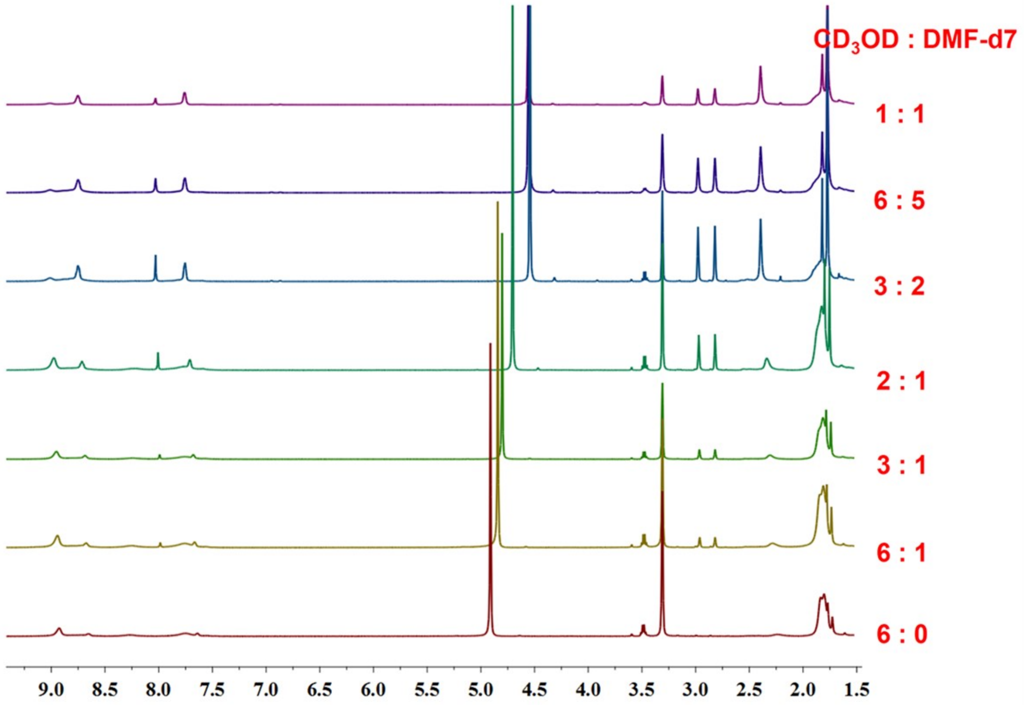

Fig. S54. The full ^{1}H NMR spectra showing the interconversion between [2] borromean ring **9b** and tetranuclear macrocycle **9a** upon changing solvent ratio (CD_3OD/DMF-d7 [20.0 mM, with respect to Cp*Rh], 500 MHz).

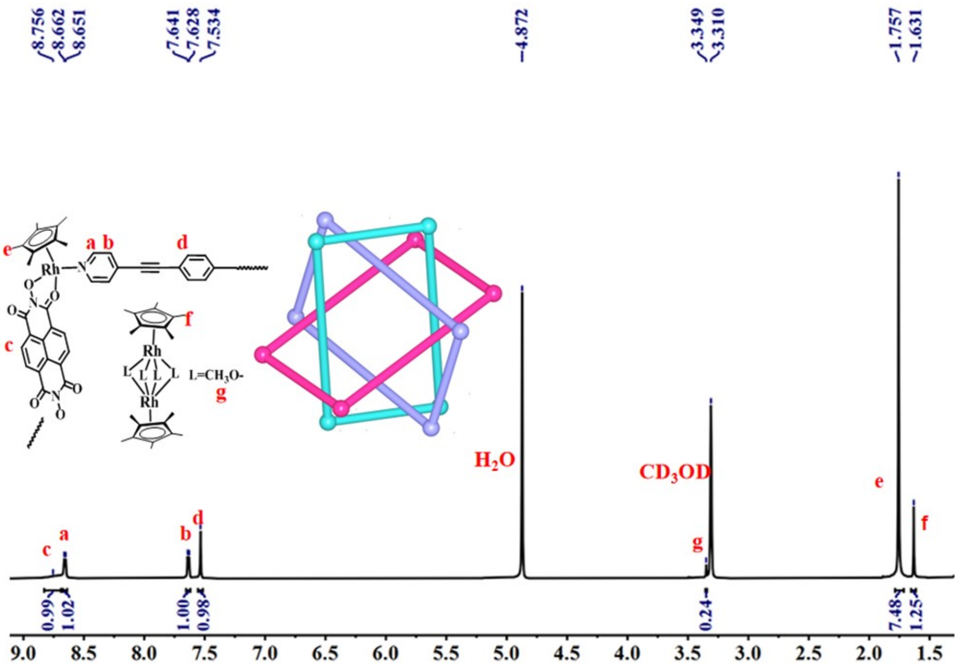

Fig. S55. The ^{1}H NMR (500 MHz, CD_3OD, ppm) for Borromean ring **10** (30.0 mM, with respect to Cp*Rh)

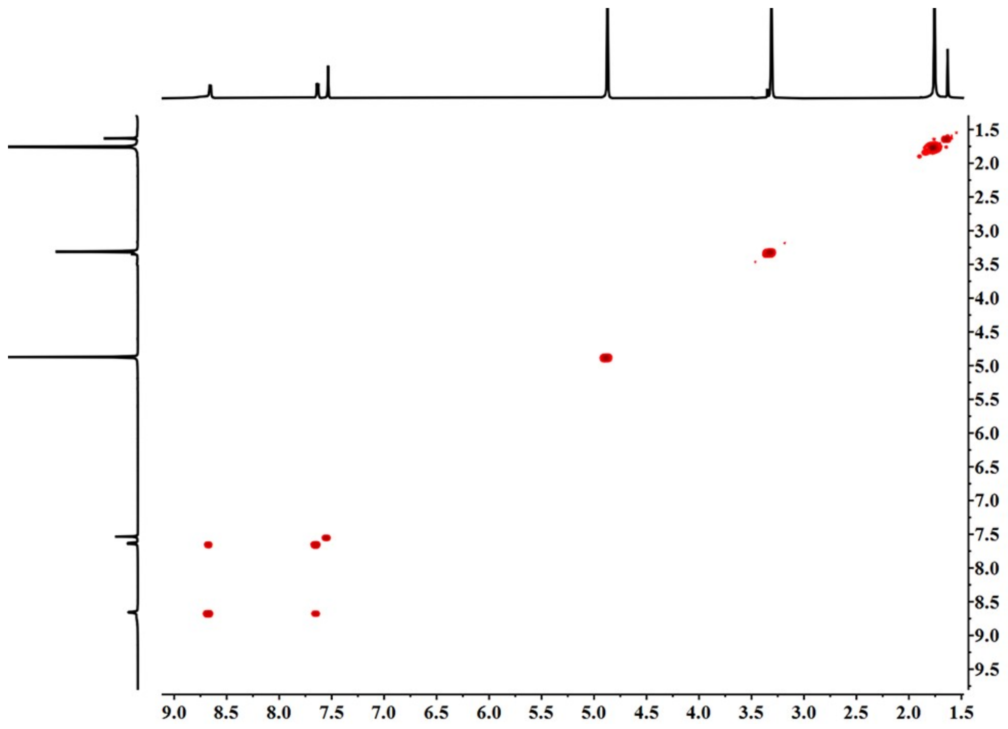

Fig. S56. The ^{1}H-^{1}H COSY NMR (500 MHz, CD_3OD, ppm) for **10** (30.0 mM, with respect to Cp*Rh)

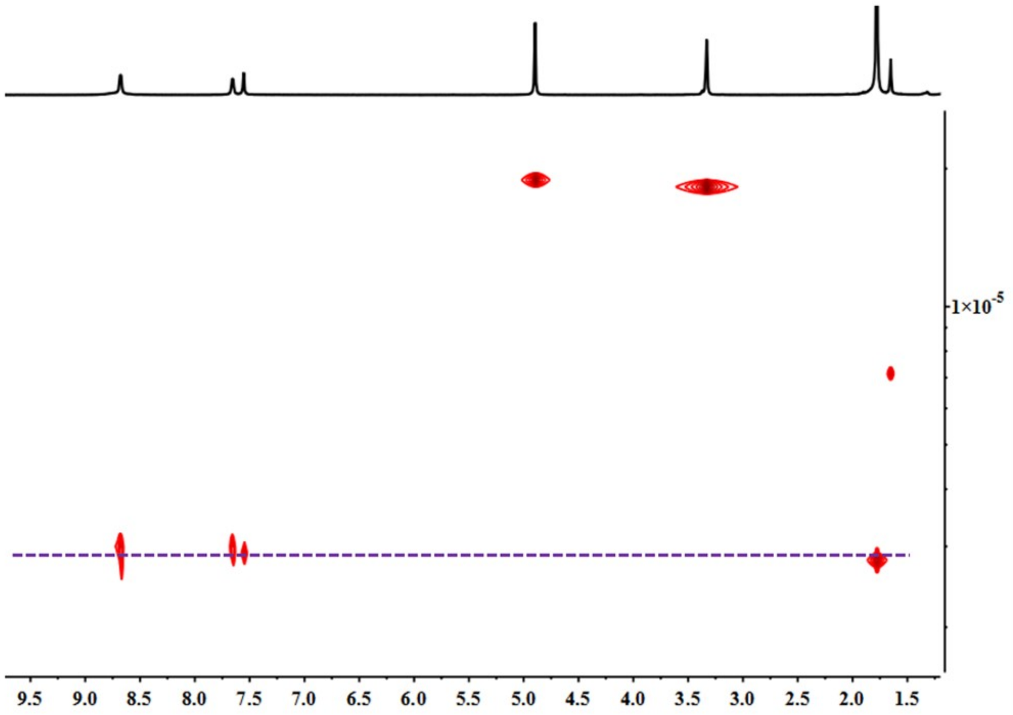

Fig. S57. The ^{1}H-^{1}H DOSY NMR (500 MHz, CD_3OD, ppm) for **10** (2.69×10^{-10} m^2s^{-1}) (30.0 mM, with respect to Cp*Rh)

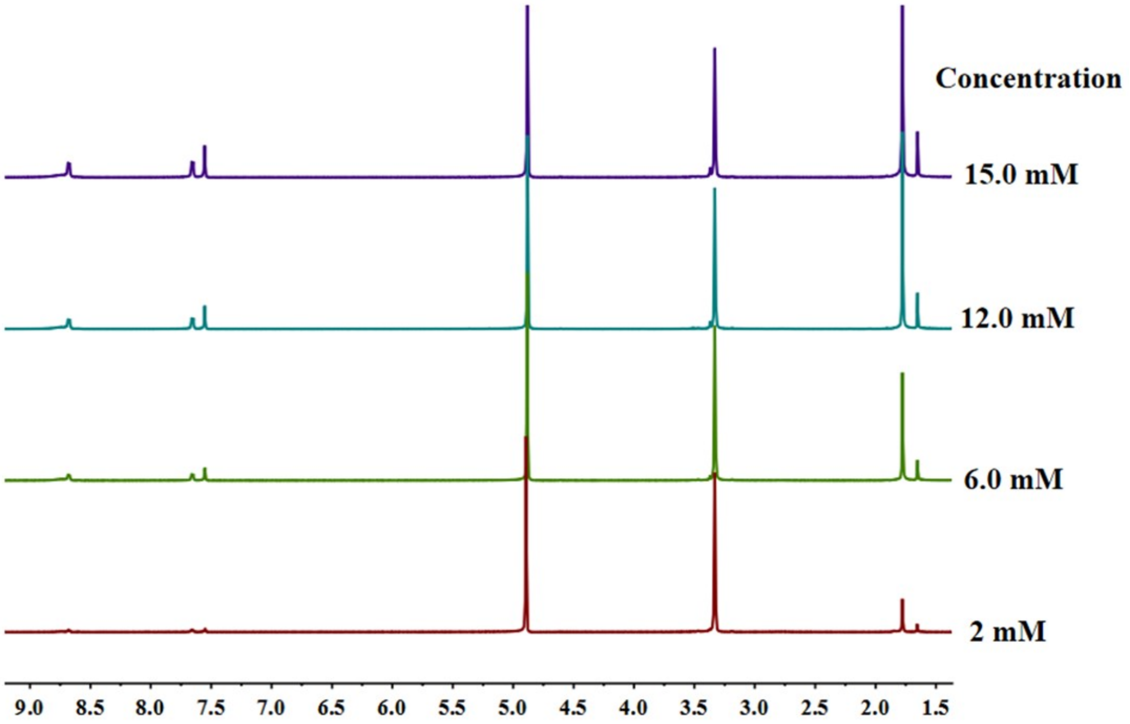

Fig. S58. The ^{1}H NMR (500 MHz, CD_3OD, ppm) for Borromean ring **10**, showing no appearance of new signals and disappearance of initial signals of **10** with an increase of concentration in CD_3OD. (2.0-15.0 mM, with respect to Cp*Rh).

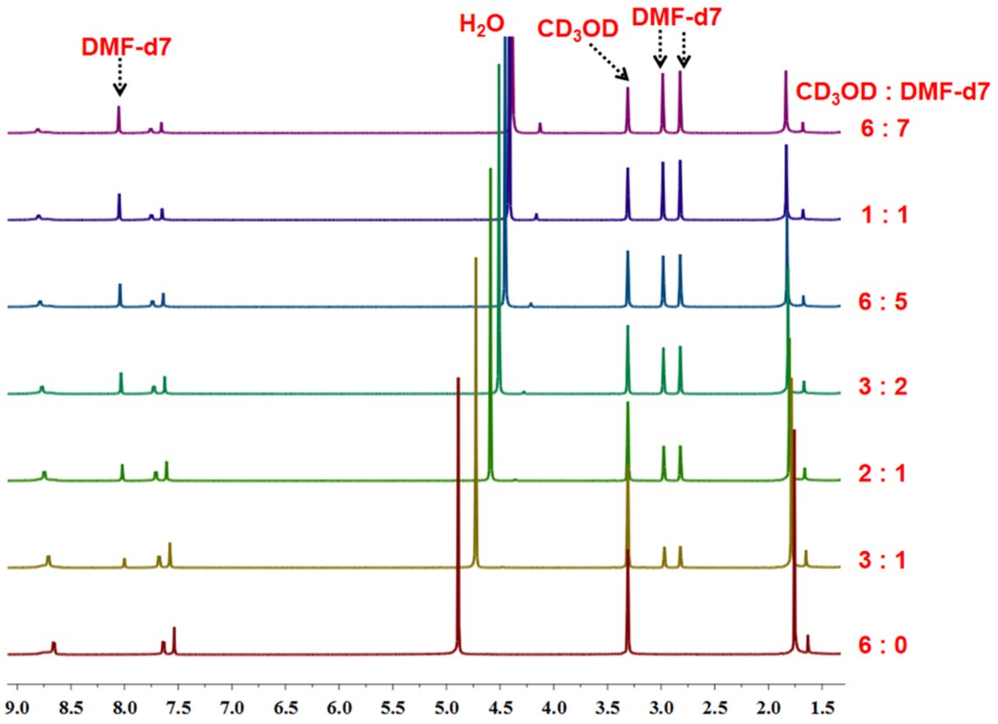

Fig. S59. The ^{1}H NMR (500 MHz, CD_3OD, ppm) for Borromean ring **10**, showing no appearance of new signals and disappearance of initial signals of **10** with an addition of DMF-d_7 in CD_3OD. (6:0-6:7).

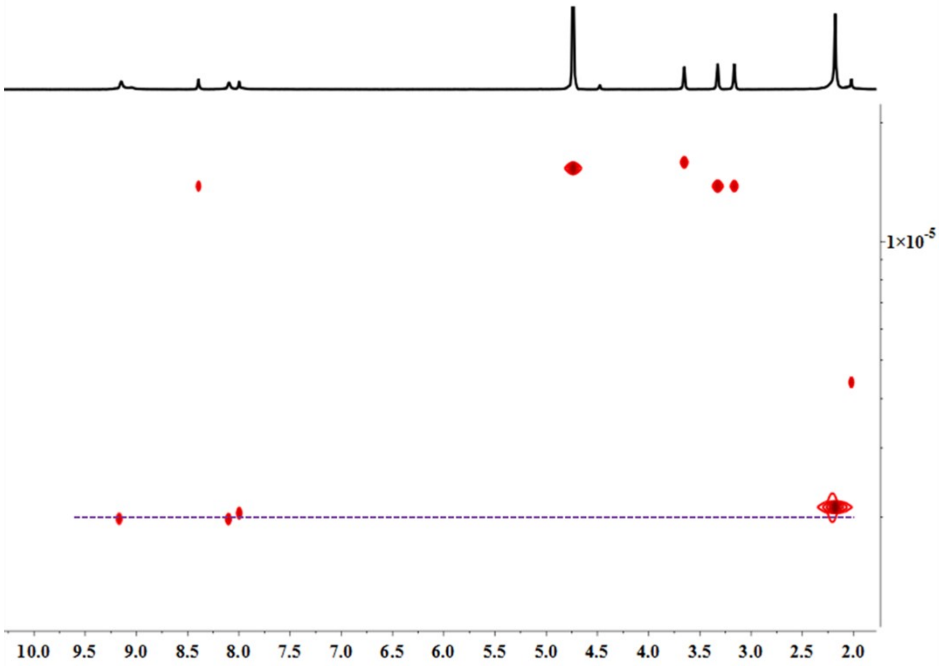

Fig. S60. The ^{1}H-^{1}H DOSY NMR (500 MHz, CD_3OD, ppm) for **10** in a 1:1 mixture of CD_3OD and DMF-d_7 (2.19×10^{-10} m^2s^{-1}) (25.0 mM, with respect to Cp*Rh).

5. ESI-MS spectra

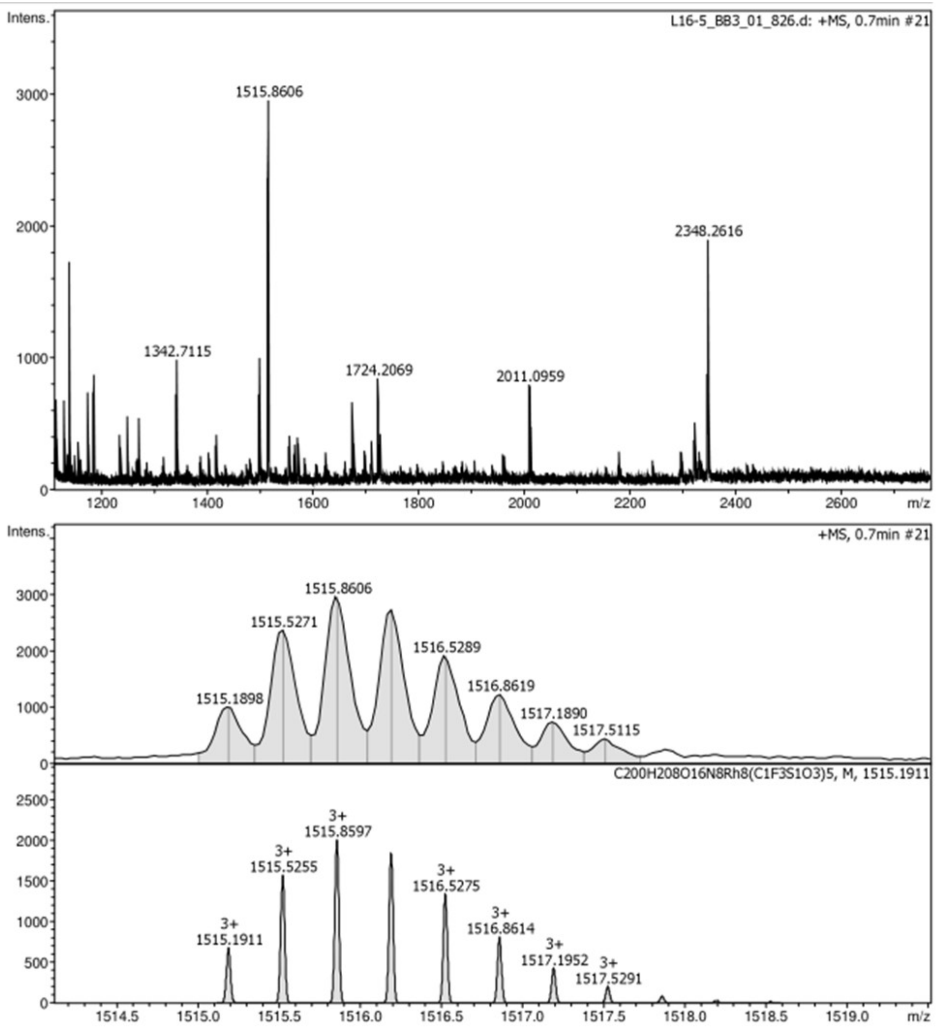

Fig. S61. Full ESI-MS spectra (**a**) of complex **3b**, experimental ESI-MS spectra of [**3b-3OTf⁻**]$^{3+}$ in CH_3OD solvent.

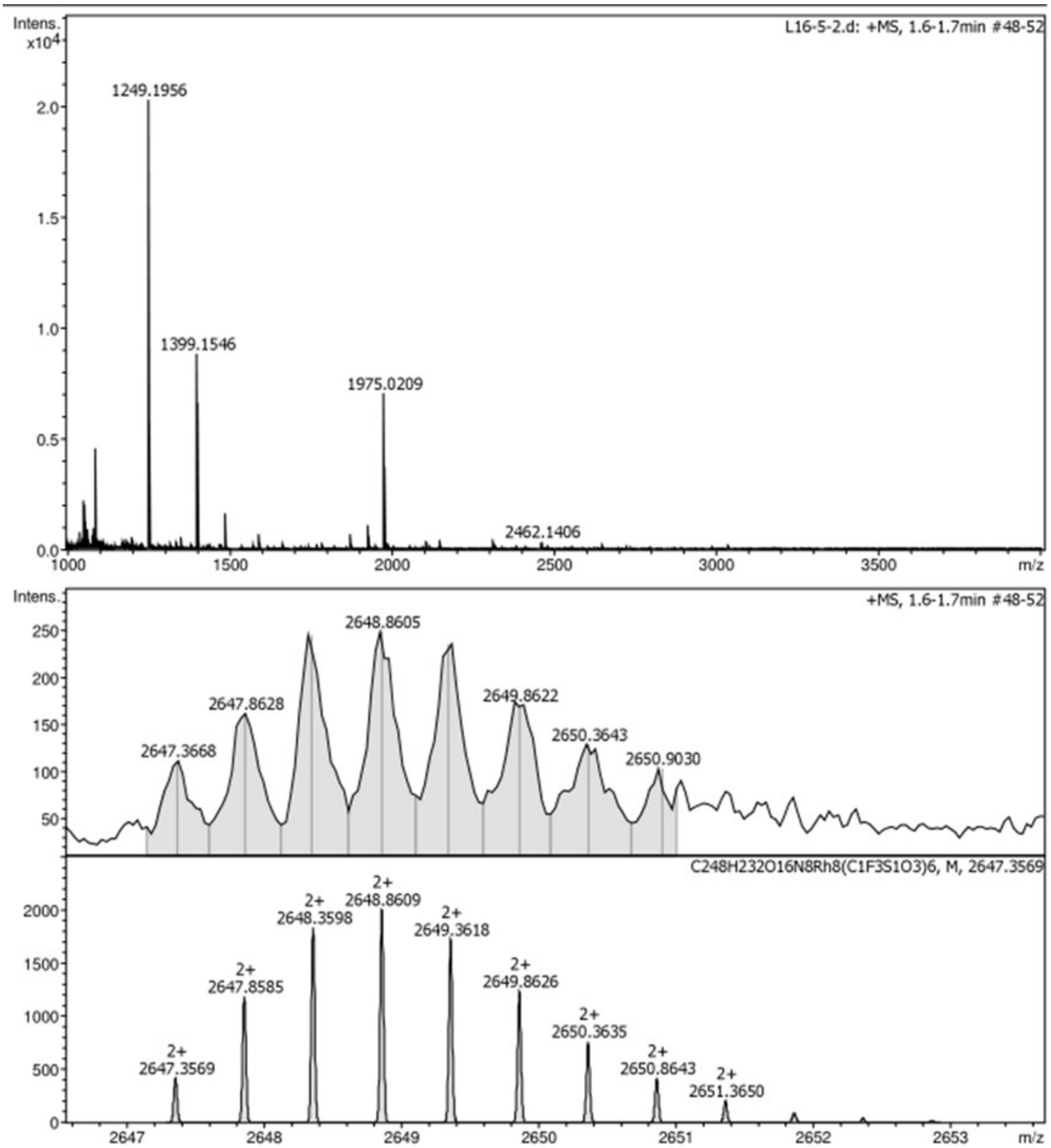

Fig. S62. Full ESI-MS spectra (**a**) of complex **5b**, experimental ESI-MS spectra of [**5b-2OTf⁻**]$^{3+}$ in CH_3OD solvent.

6. Near-infrared photothermal conversion research

1. Experimental details.

(a) Details in solution.

To guarantee same amount of conjugated-π area, the applied molar ratio of the four topologies **1/3b/4b/5b** was 2:1:1:1. Compound **1** (14.50 mg, 0.0054mmol) was added into a solvent of CH_3OH (0.5 ml). After the solid dissolved absolutely, 1.0 ml of this solution was taken into quartz spectrophotometer cell (1×1×5 cm) and put into the bright spot of a laser with 660 nm wavelength at 0.6 W/cm^2. Temperature variation of the solution was detected by an infrared camera. Compound **3b** (15.12 mg, 0.0027 mmol), compound **4b** (14.02 mg, 0.0027 mmol) and compound **5b** (13.52 mg, 0.0027 mmol), Compound **6** (12.88 mg, 0.0054 mmol), compound **7** (13.42 mg, 0.0054 mmol) and compound **8** (13.50 mg, 0.0027 mmol), were detected with the same procedure as compound 1.

(b) Details in solid state.

The crystalline compounds **1**, **3b**, **4b**, **5b** were grinded well. After that, same weight (30.0 mg) were taken in crystalline state. All these were put a sample cell. And then, they were put under a laser and temperature variation were detected by a same infrared camera as solution compounds.

Equations used to calculate near-infrared photothermal conversion efficiency were exhibited as follows:

$\eta = hS(\Delta T_{sample} – \Delta T_{solvent}) / I(1\text{-}10^{-A})$ **(1)**

$hS = \sum mC_p/\tau_s$ **(2)**

$\tau_s = -t/\ln\theta$ **(3)**

$\theta = (T_{amb} – T)/(T_{amb} – T_{max})$**(4)**

Thereinto, a solvent containing 1.0 ml methanol was used in all samples. Thus, $\sum mCp = m_{(methanol)}\, Cp_{(methanol)} = \rho_{(methanol)}\, V_{(methanol)}\, Cp_{(methanol)} = 0.791 \times 0.7 \times 2.51 = 1.3898\ J \bullet K^{-1}$. $\Delta T_{solvent} = 2.2$℃. I = 0.6 W/cm^2

Strictly speaking, volume of mixed solvent was not simple addition of two component. However, the deviation was small enough to ignore it.

2. [2]catenane 3b (with treble π−π stacking interactions).

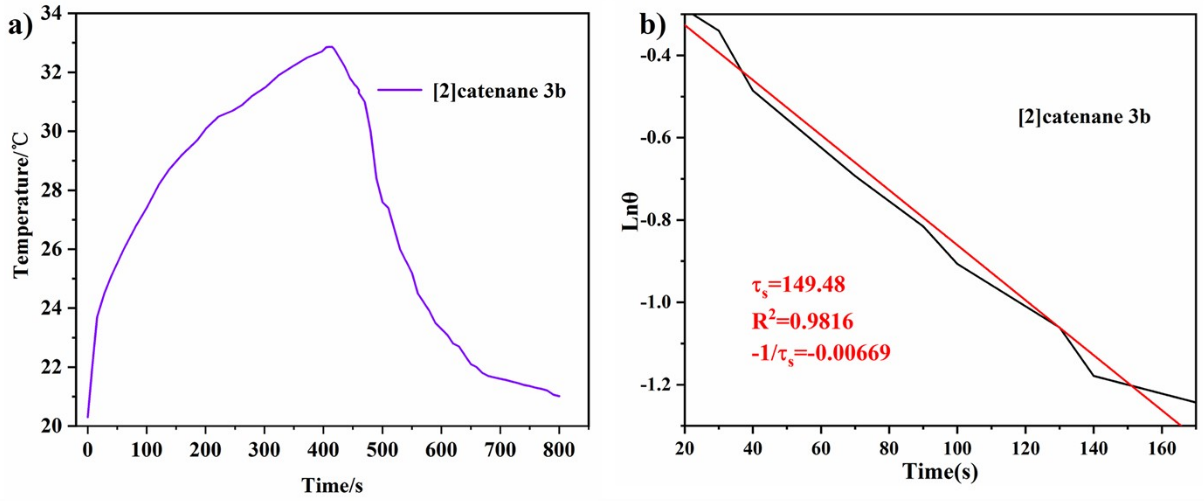

Fig. S63. (a) Heating and cooling curve of [2]catenane **3b** (with treble π−π stacking interactions); (b) Fitting linear of lnθ-t.

Near-infrared photothermal conversion efficiency η of **3b** was calculated by equations above. A fitting linear of lnθ-t was obtained by Eqs (3) and (4), by which τ_s was calculated as 149.48 s. Thus, hS = 1.3898/149.48 = 9.298×10^{-3}J·K^{-1}·S^{-1}. ΔT_{sample} = 11.1 ℃ (31.5-20.4, Fig. S56(a)). A1 = 0.1669 (Fig. 12(b) in main text). Eventually, $\eta 1$ = 9.297×10^{-2} × (11.1 – 2.2)/[0.6×(1–$10^{-0.1669}$) = 48.28 %.

3. [2]catenane 4b (with treble π−π stacking interactions).

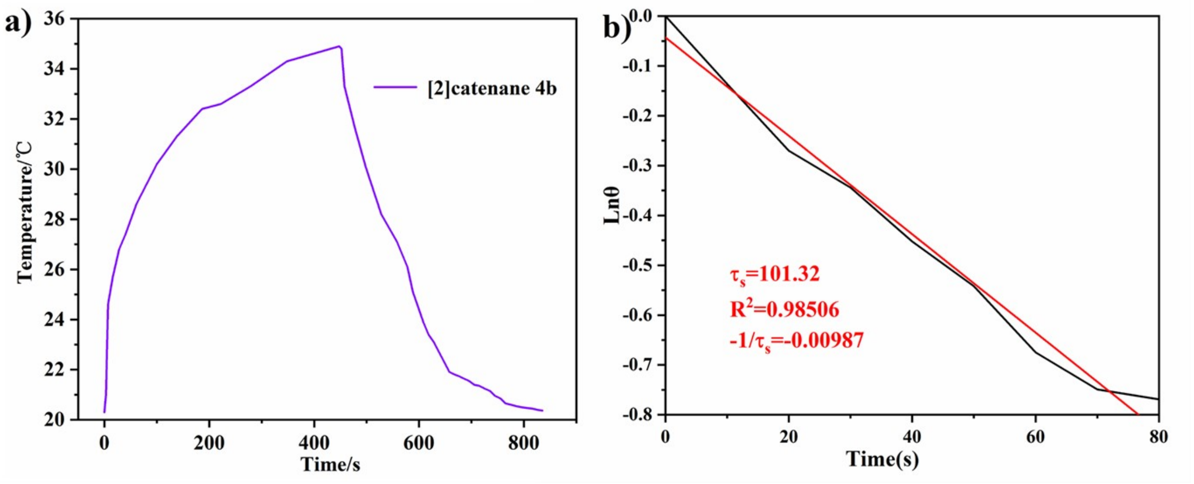

Fig. S64. (a) Heating and cooling curve of [2]catenane **4b** (with treble π−π stacking interactions); (b) Fitting linear of lnθ-t.

Near-infrared photothermal conversion efficiency η of **4b** was calculated by equations above. A fitting linear of lnθ-t was obtained by Eqs (3) and (4), by which τ_s was calculated as 101.32 s. Thus, hS = 1.38985/101.32 = 1.3717×10^{-2}J·K^{-1}·S^{-1}. ΔT_{sample} = 13.7 ℃ (35.2-20.5, Fig. S30(a)). A1 = 0.24684 (Fig. 12(b) in main text). Eventually, $\boldsymbol{\eta 1}$ = 1.3717 × 10^{-2} × (13.7 – 2.2)/[0.6×(1–$10^{-0.24684}$) = 60.62%.

4. [2]catenane 5b (with treble π−π stacking interactions).

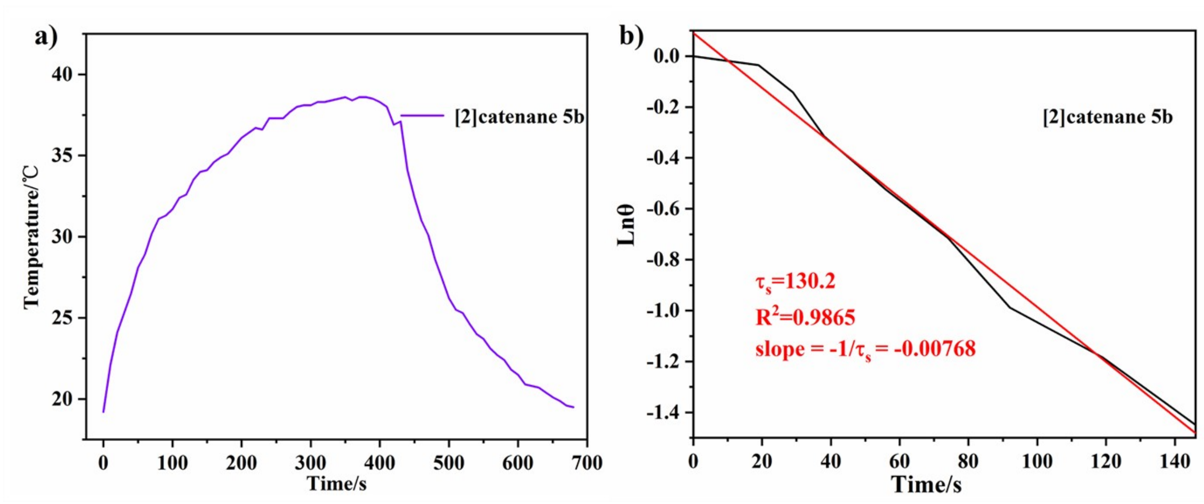

Fig. S65. (a) Heating and cooling curve of [2]catenane **5b** (with treble π−π stacking interactions); (b) Fitting linear of lnθ-t.

Near-infrared photothermal conversion efficiency η of **5b** was calculated by equations above. A fitting linear of lnθ-t was obtained by Eqs (3) and (4), by which τ_s was calculated as 130.2 s. Thus, hS = 1.3898/130.2 = 1.067×10^{-2}J·K^{-1}·S^{-1}. ΔT_{sample} = 19.8 ℃ (40.3-20.5, Fig. S57(a)). A1 = 0.24684 (Fig. 13(a) in main text). $\boldsymbol{\eta 1}$ =1.067×10^{-2}×(19.8–2.2)/[0.6×(1–10$^{-0.24684}$)= 72.21 %.

5. Metallarectangle 7 (with single π–π stacking interaction).

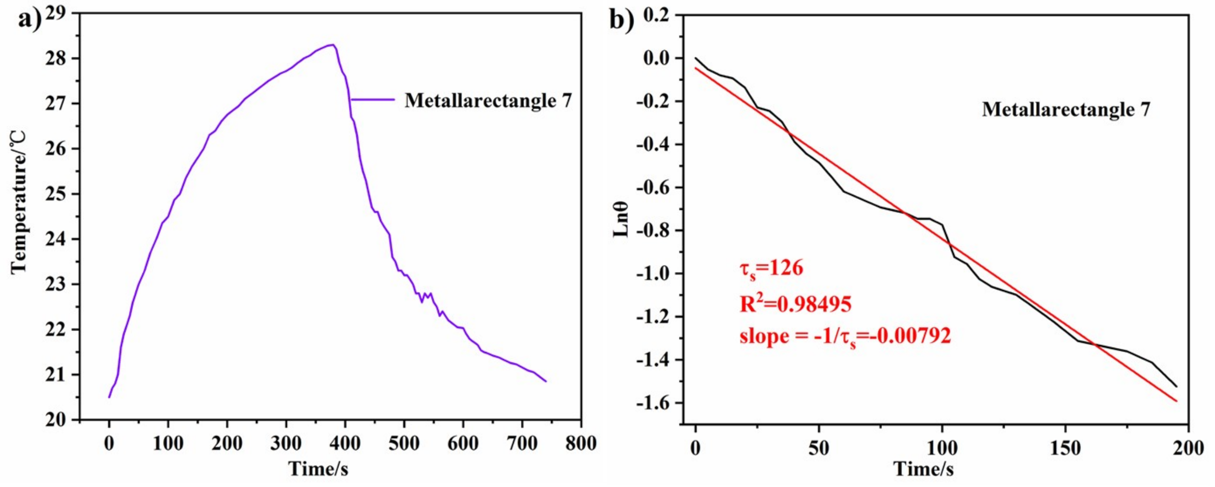

Fig. S66. (a) Heating and cooling curve of Metallarectangle **7** (with a single π-π stacking); (b) Fitting linear of lnθ-t.

Near-infrared photothermal conversion efficiency η of **7** was calculated by equations above. A fitting linear of lnθ-t was obtained by Eqs (3) and (4), by which τ_s was calculated as 126 s. Thus, hS=1.3898/120= 1.1030×10^{-2} J·K^{-1}·S^{-1}. ΔT_{sample} = 7.8 ℃ (28.2-20.4, Fig. S57(a)). A1 = 0.734 (Fig. 13(a) in main text). Eventually, $\boldsymbol{\eta_1}$ = 1.1030×10^{-2}×(7.8–2.2)/[0.6×(1–10$^{-0.734}$) = 12.64 %.

6. Borromean ring 8 (with six sets of π–π stacking interactions).

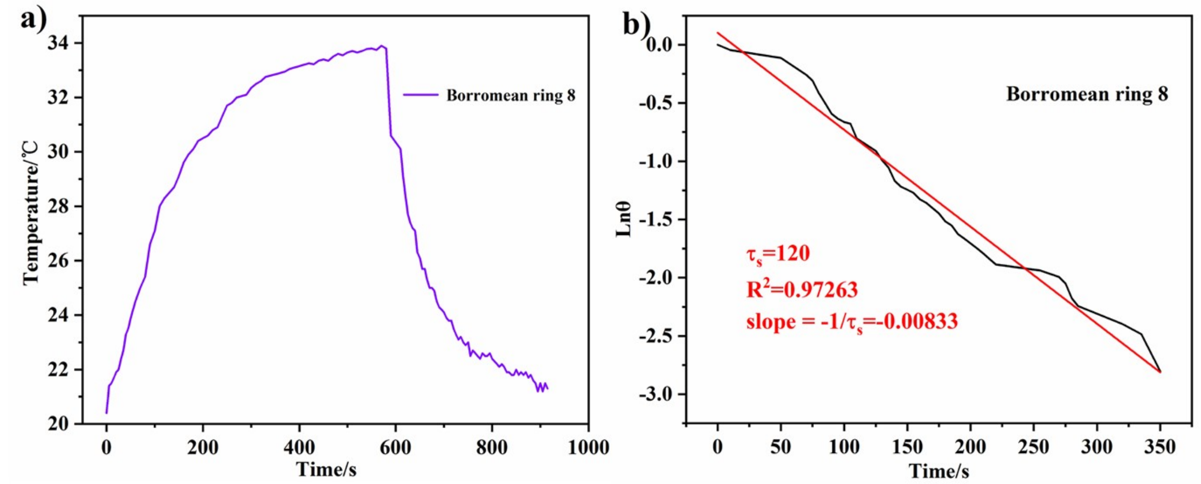

Fig. S67. (a) Heating and cooling curve of Borromean ring **8** (with six sets of π-π stacking); (b) Fitting linear of lnθ-t.

Near-infrared photothermal conversion efficiency η of **8** was calculated by equations above. A fitting linear of lnθ-t was obtained by Eqs (3) and (4), by which τ_s was calculated as 120.0 s. Thus,

hS=1.3898/120= 1.1581×10^{-2} $J\cdot K^{-1}\cdot S^{-1}$. ΔT_{sample} =13.2℃(33.6-20.4, Fig. S57(a)). A1 = 0.517 (Fig. 13(a) in main text). Eventually, η_1 =$1.1581\times10^{-2}\times(13.2–2.2)/[0.6\times(1–10^{-0.517})$=30.53 %.

7. X-ray crystallography details

Single crystals of **1**, **3b**, **5b**, **7**, **8**, **9b** and **10**, suitable for X-ray diffraction study were obtained at room temperature. X-ray intensity data of them were collected at 250, 173, 150, 173, 150, 173 K and 193K on a CCD-Bruker SMART APEX system. In these data, the disordered solvent molecules which could not be restrained properly were removed using the PLATON Squeeze routine.

In asymmetric unit of **1**, a solvent mask was calculated and 722 electrons were found in a volume of 2146\%A^3^ in 1 void per unit cell. This is consistent with the presence of $5[CF_3SO_3]$ per Asymmetric Unit which account for 730 electrons per unit cell.

In asymmetric unit of **3b**, a solvent mask was calculated and 462 electrons were found in a volume of 2665\%A^3^ in 4 voids per unit cell. This is consistent with the presence of $3.5[CH_3OH]$, $1[C_6H_{14}O]$ per Asymmetric Unit which account for 484 electrons per unit cell.

In asymmetric unit of **5b**, a solvent mask was calculated and 5774 electrons were found in a volume of 15988\%A^3^ in 1 void per unit cell. This is consistent with the presence of $20[CF_3SO_3]$ per Asymmetric Unit which account for 5840 electrons per unit cell.

In asymmetric unit of **7**, a solvent mask was calculated and 260 electrons were found in a volume of 1098\%A^3^ in 3 voids per unit cell. This is consistent with the presence of $2.96[CF_3SO_3]$, $1.04[CF_3SO_3]$ per Asymmetric Unit which account for 292 electrons per unit cell.

In asymmetric unit of **8**, a solvent mask was calculated and 1389 electrons were found in a volume of 6341\%A^3^ in 1 void per unit cell. This is consistent with the presence of $5[CF_3SO_3]$ per Asymmetric Unit which account for 1460 electrons per unit cell.

In asymmetric unit of **10**, a solvent mask was calculated and 2548 electrons were found in a volume of 14290\%A^3^ in 1 void per unit cell. This is consistent with the presence of $9[CF_3SO_3]$ per Asymmetric Unit which account for 2628 electrons per unit cell.

Table 1 Crystal data and structure refinement for 1.

Empirical formula	$C_{123}H_{116}F_{21}N_{12}O_{21}Rh_4S_7$
Formula weight	3133.33
Temperature/K	249.99(10)
Crystal system	Monoclinic
Space group	*P2₁/c*
a/Å	*24.6083(8)*
b/Å	*12.7106(5)*
c/Å	*23.5913(8)*
α/°	*90*
β/°	*114.206(4)*
γ/°	*90*

Volume/Å^3	6730.2(5)
Z	2
ρcalcg/cm^3	1.546
μ/mm^{-1}	5.766
F(000)	3174.0
Crystal size/mm^3	0.14 × 0.13 × 0.11
Radiation	Cu Kα (λ = 1.54178)
2Θ range for data collection/°	7.516 to 148.622
Index ranges	-28 ≤ h ≤ 30, -15 ≤ k ≤ 15, -29 ≤ l ≤ 20
Reflections collected	28919
Independent reflections	13295 [Rint = 0.0698, Rsigma = 0.0861]
Data/restraints/parameters	13295/1143/681
Goodness-of-fit on F^2	1.025
Final R indexes [I>=2σ (I)]	R1 = 0.0737, wR2 = 0.2033
Final R indexes [all data]	R1 = 0.0945, wR2 = 0.2326
Largest diff. peak/hole / e Å^{-3}	1.39/-1.16

Table 2 Crystal data and structure refinement for 3b

Empirical formula	$C_{113.5}H_{132}F_{12}N_4O_{24.5}Rh_4S_4$
Formula weight	2712.10
Temperature/K	173(2)
Crystal system	monoclinic
Space group	*P2₁*
a/Å	*15.5383(4)*
b/Å	*19.2201(5)*
c/Å	*41.5739(12)*
α/°	*90*
β/°	*94.273(2)*
γ/°	*90*
Volume/Å^3	12381.4(6)
Z	4
ρcalcg/cm^3	1.455
μ/mm^{-1}	0.677
F(000)	5556.0
Crystal size/mm^3	0.110 × 0.080 × 0.080
Radiation	MoKα (λ = 0.71073)
2Θ range for data collection/°	2.948 to 51.448
Index ranges	-18 ≤ h ≤ 17, -23 ≤ k ≤ 23, -50 ≤ l ≤ 50
Reflections collected	158418
Independent reflections	46905 [Rint = 0.0664, Rsigma = 0.0697]
Data/restraints/parameters	46905/7618/2617
Goodness-of-fit on F^2	1.013
Final R indexes [I>=2σ (I)]	R1 = 0.0834, wR2 = 0.2272
Final R indexes [all data]	R1 = 0.1100, wR2 = 0.2525
Largest diff. peak/hole /eÅ^{-3}	2.62/-0.68

Table 3. Crystal data and structure refinement for 5b

Empirical formula	$C_{268}H_{233}F_{60}N_8O_{76}Rh_8S_{20}$
Formula weight	7386.09
Temperature/K	150.00(10)
Crystal system	orthorhombic
Space group	*Pna21*
a/Å	*29.3957(12)*
b/Å	*27.2297(11)*
c/Å	*40.625(3)*
α/°	*90*
β/°	*90*
γ/°	*90*
Volume/Å^3	32518(3)
Z	4
ρcalcg/cm^3	1.509
μ/mm^{-1}	5.368
F(000)	14900.0
Crystal size/mm^3	0.14 × 0.11 × 0.08
Radiation	Cu Kα (λ = 1.54178)
2Θ range for data collection/°	7.48 to 149.664
Index ranges	$-27 \leq h \leq 36$, $-33 \leq k \leq 17$, $-50 \leq l \leq 39$
Reflections collected	81531
Independent reflections	48239 [Rint = 0.0906, Rsigma = 0.1696]
Data/restraints/parameters	48239/7595/2259
Goodness-of-fit on F^2	1.016
Final R indexes [I>=2σ (I)]	R1 = 0.0973, wR2 = 0.1601
Final R indexes [all data]	R1 = 0.2019, wR2 = 0.2025
Largest diff. peak/hole / e Å^{-3}	0.74/-0.57

Table 4. Crystal data and structure refinement for 7

Empirical formula	$C_{232}H_{240}F_{24}N_8O_{44}Rh_8S_8$
Formula weight	5380.07
Temperature/K	193.00
Crystal system	monoclinic
Space group	$P2_1$/c
a/Å	*21.381(12)*
b/Å	*15.550(6)*
c/Å	*19.181(6)*
α/°	*90*
β/°	*98.216(15)*
γ/°	*90*
Volume/Å^3	6312(5)
Z	1
ρcalcg/cm^3	1.415
μ/mm^{-1}	3.681
F(000)	2744.0
Crystal size/mm^3	0.24 × 0.22 × 0.2
Radiation	GaKα (λ = 1.34139)
2Θ range for data collection/°	6.178 to 107.802
Index ranges	$-25 \leq h \leq 25$, $-18 \leq k \leq 12$, $-22 \leq l \leq 23$
Reflections collected	44523
Independent reflections	11489 [R_{int} = 0.0479, R_{sigma} = 0.0424]
Data/restraints/parameters	11489/1514/841
Goodness-of-fit on F^2	1.089
Final R indexes [I>=2σ (I)]	R1 = 0.0705, wR2 = 0.2208
Final R indexes [all data]	R1 = 0.0890, wR2 = 0.2402
Largest diff. peak/hole /eÅ^{-3}	1.61/-0.98

Table 5. Crystal data and structure refinement for 8

Empirical formula	$C_{366}H_{300}F_{54}N_{12}O_{78}Rh_{12}S_{18}$
Formula weight	8952.16
Temperature/K	173.00
Crystal system	monoclinic
Space group	*P2₁/n*
a/Å	*23.670(2)*
b/Å	*36.843(3)*
c/Å	*25.731(2)*
α/°	*90*
β/°	*112.251(4)*
γ/°	*90*
Volume/Å^3	20768(3)
Z	2
ρcalcg/cm^3	1.432
μ/mm^{-1}	3.586
F(000)	9036.0
Crystal size/mm^3	0.24 × 0.22 × 0.20
Radiation	GaKα (λ = 1.34138)
2Θ range for data collection/°	5.974 to 107.278
Index ranges	-27 ≤ h ≤ 28, -29 ≤ k ≤ 43, -30 ≤ l ≤ 23
Reflections collected	125192
Independent reflections	36699 [Rint = 0.1129, Rsigma = 0.1269]
Data/restraints/parameters	36699/4485/2086
Goodness-of-fit on F^2	1.093
Final R indexes [I>=2σ (I)]	R1 = 0.1207, wR2 = 0.2638
Final R indexes [all data]	R1 = 0.1800, wR2 = 0.2901
Largest diff. peak/hole/eÅ^{-3}	2.04/-1.65

Table 6. Crystal data and structure refinement for 10

Empirical formula	$C_{366}H_{318}F_{54}N_{24}O_{94}Rh_{14}S_{18}$
Formula weight	9600.25
Temperature/K	193.00
Crystal system	monoclinic
Space group	*P2₁/c*
a/Å	*20.474(5)*
b/Å	*34.090(9)*
c/Å	*38.801(10)*
α/°	*90*
β/°	*105.086(12)*
γ/°	*90*
Volume/Å^3	26149(11)
Z	2
ρcalcg/cm^3	1.219
μ/mm^{-1}	3.256
F(000)	9676.0
Crystal size/mm^3	0.23 × 0.22 × 0.2
Radiation	Ga Kα (λ = 1.34139)
2Θ range for data collection/°	4.496 to 108.818
Index ranges	-24 ≤ h ≤ 24, -41 ≤ k ≤ 41, -46 ≤ l ≤ 45
Reflections collected	137431
Independent reflections	47779 [Rint = 0.0699, R_{sigma} = 0.0776]
Data/restraints/parameters	47779/5532/1793
Goodness-of-fit on F^2	1.146
Final R indexes [I>=2σ (I)]	R1 = 0.1024, wR2 = 0.2124
Final R indexes [all data]	R1 = 0.1552, wR2 = 0.2336
Largest diff. peak/hole/eÅ^{-3}	1.38/-1.37

8. References

1. C. White, A. Yates and P. M. Maitlis, *η*5 -Pentamethylcyclopentadienyl) rhodium and -iridium compounds. *Inorg. Synth*., **1992**, *29*, 228–234.
2. T. Wu, L. H. Weng and G. X. Jin, Sunlight induced cycloaddition and host–guest property of self-assembled organometallic macrocycles based on a versatile building block. *Chem. Comm*., **2012**, *48*, 4435–4437.
